# Climate change adaptation measures conflicted with the recreational demands on city forests during COVID-19 pandemic

**DOI:** 10.1038/s42949-023-00096-y

**Published:** 2023-03-15

**Authors:** Angela Beckmann-Wübbelt, Lynn Türk, Iulia Almeida, Annika Fricke, Metodi Sotirov, Somidh Saha

**Affiliations:** 1 Institute for Technology Assessment and Systems Analysis (ITAS), Karlsruhe Institute of Technology, Karlstr. 11, 76133 Karlsruhe, Germany; 2 Chair of Forest and Environmental Policy, University of Freiburg, Tennenbacherstr. 4, 79106 Freiburg im Breisgau, Germany; 3 Institute for Geography and Geoecology (IfGG), Karlsruhe Institute of Technology, Kaiserstr. 12, 76131 Karlsruhe, Germany

**Keywords:** Forestry, Psychology and behaviour

## Abstract

Recurrent droughts in southwest Germany threaten the city and community-owned forests (CCF). At the same time, the COVID-19 pandemic has increased the demand for recreation in CCF of southwest Germany. We interviewed stakeholders from different interest groups to critically analyze their opinion on how the high recreation demand on CCF due to the pandemic can be ensured along with implementing climate change adaptation measures in CCF in Karlsruhe, Germany. We found that stakeholders particularly highlighted the importance of the recreational function of the CCF during the pandemic. However, the behavior of visitors was criticized by the stakeholders. We showed that demand for the recreational use of CCF conflicted with climate change adaptation measures such as sanitary and forest restoration actions, creating a dilemma among stakeholders. Therefore, enhancing citizens’ knowledge of forests’ recreation functions and the need for climate change adaptation through communication and education should be prioritized.

## Introduction

Cities worldwide are expanding, and urban green infrastructure in cities is suffering from the ever-increasing urbanization and associated land-use changes^1,2^. This threat of urbanization is frequently occurring in Europe^3^. Further, climate change impacts such as increasing drought events and heat stress^4^ and vigor-related diseases caused by environmental stresses and human activities^5^ lead to a decrease in the benefits that urban green infrastructure can provide.

Urban forests form part of the green infrastructure. These include clumps of trees in both large (>0.5 ha) and small (<0.5 ha) parks, gardens, and street trees or public spaces within a city^1^. City and community-owned forest (CCF) include parts of the urban forest owned and managed by the city itself^6^. Breuste^7^ stated that urban forest functions can be summarized in three main aspects: a recreational function, an ecological protective function, and an economic utility function for the urban population. CCF, however, plays an assorted and important role as its management is mainly dedicated to the urban population’s well-being^8^. Thereby, CCF offers a wide range of services that improve urban residents’ physical and mental recreation and well-being^9,10^. CCF contributes to the esthetics of the urban landscape^11^, provides space for sporting activities^12^, and serves as a meeting place for the urban population^13^.

Especially during restriction times of the COVID-19 pandemic, visitor numbers to the CCF increased. The COVID-19 pandemic pointed attention towards increased recreational use and the high importance of the urban forest for the physical and mental health of the urban population^14,15^. Numerous authors such as Erdönmez and Atmiş^16^ highlight the lessons learned from the pandemic. They call for strengthening the recreational function of CCF in the future.

However, CCFs’ provision of recreational values for the urban population is under threat. The increased danger for visitors when visiting the forest due to falling trees and branches after recent droughts were reported all over Germany in news articles and local administrations’ warnings^17–24^. For example, due to drought, the roots of many trees in the Odenwald near Darmstadt, a town in the state of Hessen, Germany, no longer reach the groundwater^25^. As a result, branches, treetops, and entire trees die. This brings along an increasing danger to safety for a growing number of forest visitors who could be hit by falling branches or even falling trees. Therefore, the city of Darmstadt has already had to close parts of the forest for the safety of forest visitors^25^. The important recreational function of the forest for the urban population can no longer be achieved this way. In particular, the extreme drought of 2018 significantly reduced the vitality of trees in Germany^26^.

At the same time, it has been highlighted in recent studies that an increased number of visitors to CCF due to the COVID-19 pandemic may lead to conflicts. Derks et al.^27^ pointed out potential trade-offs between the recreational function and forest management operations. Further, an increased number of visitors may lead to a decrease in the habitat function of animals of the CCF. Conflicts between different functions of CCF could potentially also lead to the disturbance of climate change adaptation measures due to more visitors. Such trade-offs occurring during the COVID-19 pandemic and how they are perceived by different stakeholders who influence the CCF management are yet rarely studied. However, it is expected that the comparison of perspectives from different stakeholder groups can lead to improved reflection and strengthen the sustainable management of the CCF through knowledge-based and participative decision-making^28^.

In the past, climate change adaptation has been recognized in several studies focusing on different stakeholder perceptions and has been on the agenda of urban forest planners. Živojinović and Wolfslehner^29^ assessed the perceptions of urban forestry stakeholders on climate change adaptation in Serbia. Tran et al.^30^ conducted a similar study in the United States. Both authors highlighted the importance of the urban forests for climate adaptation and the need to adapt the forest itself.

However, different groups and individuals in the urban population have different needs. Thus, they have different expectations of urban green spaces and forests and their numerous functions^31^. One of the most significant challenges in urban forest management is to meet all needs and, at the same time, maximize the provision of various services and avoid trade-offs^32^. Different preferences of different interests and user groups of urban forests can lead to conflicts in planning natural resources in cities^33^. Therefore, it is critical how the multitude of local and national stakeholders are involved in decision-making regarding the urban forest^34,35^. Since management decisions^36^, as well as other human interventions in nature^37^, can lead to forest function trade-offs, the importance of investigating and incorporating the expectations of the urban forest of different stakeholders is highlighted to ensure the quality of life in cities successfully. Gaining a better understanding of the way in which urban forests are being prioritized, planned, and managed by stakeholders and how these stakeholders work together can inform strategies for improving urban forest governance and management, urban biodiversity conservation, urban green equity, and the quality of life of urban dwellers^38^.

The World Health Organization (WHO)^39^ recommends that cities take action by providing safe open spaces, such as urban forests, accessible to the public, thereby promoting mental well-being. However, it is not yet known how such promotion of the recreational function of urban forests may conflict with safeguarding other functions such as climate change adaptation of the urban forest.

In this study, we aim to evaluate how different stakeholder groups involved in urban forest management evaluate newly emerging conflict fields between climate change adaptation and increased recreational use of the urban forest. Specifically, three research questions were formulated for this study:

1. How do different stakeholder groups perceive the challenges of climate change adaptation for CCF, and which solutions do they highlight as important?
2. What new conflicts and fields of tension between the implications of the COVID-19 pandemic and climate change adaptation in CCF do different stakeholders identify?
3. What solutions do different stakeholder groups provide to address these challenges and maximize the increased demand for recreation in CCF during the COVID-19 pandemic while simultaneously adapting to the changing climate?

## Results

### The divergence between Stakeholder groups

The mean of the occurrences of the code segments in the performed qualitative interviews was calculated to compare the stakeholders’ arguments within and between groups. In terms of average similarity per stakeholder group, the NGOs and Associations group is most similar, with 74% of accordance in the occurrence of coded segments. The coded segments of the Scientists and Professionals group are also relatively homogenous with 72% accordance. The Administration stakeholder group is the least similar, with a mean value of ~67%.

Between the groups, most divergences occurred between Scientists and Professionals and the administration (63% accordance), followed by the Scientists, Professionals, and the NGOs and Associations (65% accordance). In the interviews, one administration member also stated that cooperation with experts from science should be increased. All results are shown in Table 1.

**Table 1:** Accordance of the occurrence of coded segments between different stakeholders.

	NA	A	SP
NA	74%	69%	65%
A		67%	63%
SP			72%

*NA* NGOs and Associations, *A* Administration, *SP* Scientists and Professionals.

### Stakeholders’ perceptions on climate change adaptation of CCF

Before examining the stakeholders’ opinions on climate change adaptation and its challenges for the urban forest, the knowledge of the current climate change adaptation plan (CAP) in Karlsruhe was evaluated. It was discovered that all stakeholders from Non-governmental organiztions (NGOs) and associations were aware and knew the CAP of Karlsruhe. This is unique as other stakeholder groups and individuals were unfamiliar with it. Of those who are aware of the CAP and its suggestions for future planning, the group of NGOs and associations evaluated it as solely negative, while stakeholders working in the administration rated the plan and its implementation as predominantly positive. The scientists and professionals highlighted the positive aspects of the CAP, even though they criticized the negligent action against neophytes.

**Fig. 1:**
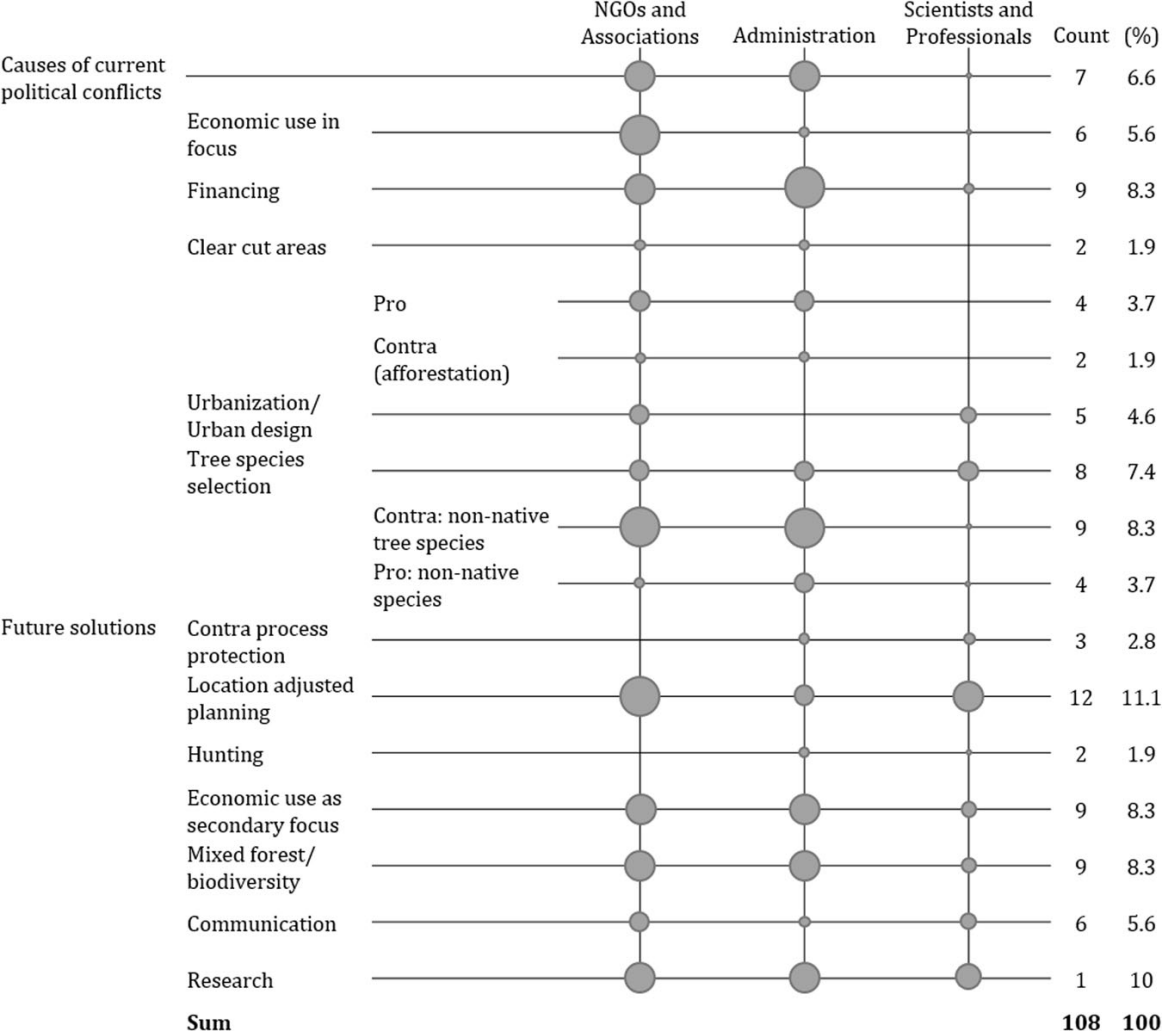
Political conflicts and future solutions for the climate change adaptation of CCF. Boxes representing the average quantity of segmented codes per interviewee per stakeholder group. Additionally, the total number of codes identified is indicated.

Even though most stakeholders rated the CAP and its implementation as favorable, numerous political conflicts in climate adaptation policies have been raised during the interviews. Figure 1 shows the result of the analysis regarding the coded segments of the interviews on climate change adaptation in the CCF in Karlsruhe. These include both current challenges and future solutions highlighted by the stakeholders. The most often raised aspect of challenges in climate change adaptation of the urban forest was financing, followed by an extensive debate on the use of non-native species.

Members of the NGOs and associations as well as from the administration in particular, often speak out against planting non-native tree species. According to those stakeholders, non-native trees have a low ecological value and offer inadequate habitat for the local fauna.

“And, as I have already said, they should be native tree species because they are much more valuable as a food source for our native insects, especially butterfly caterpillars, than any introduced tree species to which the native fauna is not actually adapted” (NA3).

At the same time, opinions on this topic differ within the administration group. Although arguments for non-native tree species are mentioned less frequently, some interviewees also advocate their plantings, especially in locations exposed to extreme weather such as droughts and heat.

“My professional opinion is that we should of course keep the basic structure - if possible from native tree species as long as possible - but that we also need a certain proportion of non-native tree species that have proven themselves in our country. […] Especially in difficult locations with extremely dry, sandy soil, I think that we need a certain proportion of non-native tree species” (A3).

The scientists and professionals also partly supported arguments supporting the use of non-native species.

“I think under the current climate change conditions we will not be able to fully abstain from non-native species. And, if one orients himself with the nature-near forest economy of the FSC guidelines this would also not be a problem. Since 30% of non-native species could be integrated” (SP1).

The administration group and NGOs highlight funding as a particular problem, while the academics say little on the subject. Arguments include suggestions and opinions on how measures for financing the climate change adaptation strategy should be carried out. It is argued that politics need to draw more attention to the importance of the urban forest’s climate change adaptation and budgets need to be allocated accordingly.

“There would have to be a political decision to establish more urban greenery, so that would be the point I think. […] The second point is of course always the deadly argument of financing: who should pay for all that stuff? Yes, of course you have to think about it, but I would also say that politics is also responsible for that, I mean they have this post to think about it. There is a certain budget that could perhaps also be restructured“ (SP5).

Another frequently highlighted problem in adapting to climate change, particularly by NGOs and associations, is that the focus is too often on the economic benefits of the forest. Such arguments highlight that politics often prefer the economic use of the forest over its function for climate change adaptation. Scientists and professionals highlight increasing urbanization as one of the most threatening reasons for conflicts in urban climate adaptation policy. It is highlighted that one special feature of the urban forest is its proximity and importance to city dwellers.

The most often raised future solution for the climate adaptation of the urban forest that the stakeholders have raised is location-adjusted planning, which has been highlighted 12 times during the interviews. Arguments coded in this category highlight the importance of site-specific conditions and functions of the forest in the field of climate change adaptation. Further, increased research, which was mentioned 11 times, is highlighted as a possible future solution for increasing the urban forests’ climate change adaptation. Stakeholders argue that the solution to the identified conflict areas in climate change adaptation, especially regarding tree species selection, lies in research and years of experience, as well as the incorporation of scientific findings on climate change consequences and scenarios. Both issues are raised frequently by all stakeholder groups and have been highlighted above other future solutions, especially in the group of scientists and professionals.

“After all, I don’t want the land use decisions on the outskirts of Karlsruhe-Hardtwald to be made somewhere centrally in Berlin or Brussels or perhaps internationally in New York. That’s nonsense. Ultimately, the decision must be taken locally” (SP2).

“The administration employees are certainly not the only ones who have an idea; there are many other people, experts, and scientists, who have special knowledge in their fields […]. They have to be exchanged; they also have to be incorporated” (A3).

The administration group mentions increased biodiversity and the deferral of economic benefits more frequently. The group of NGOs emphasizes these issues but they find little attention in the group of scientists and professionals.

### Emerging conflicts between climate change adaptation and recreation in CCF management

**Fig. 2:**
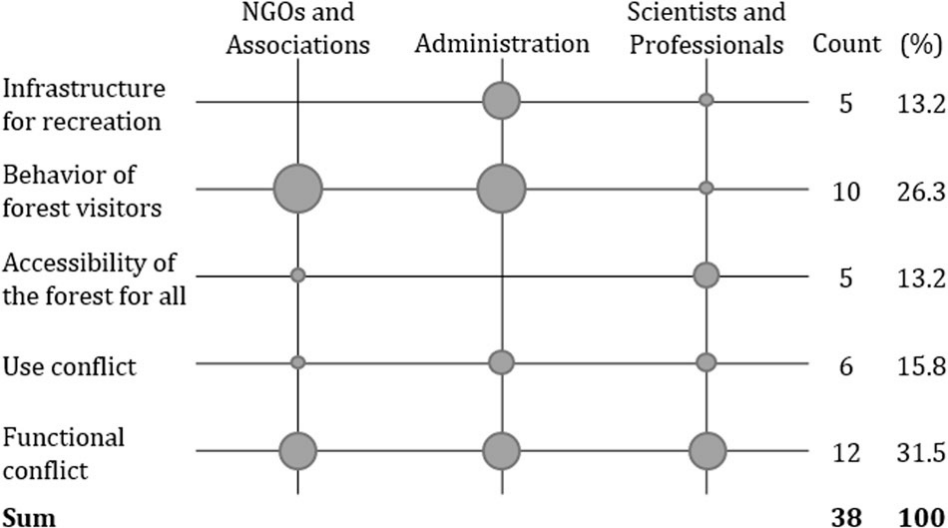
Emerging conflicts between climate change adaptation and the recreational function of CCF. Boxes represent the average quantity of segmented codes per interviewee per stakeholder group. Additionally, the total number of codes identified is indicated.

The second research question focused on the issue of conflicts and fields of tension between the implications of the COVID-19 pandemic and climate change adaptation in the urban forest. Figure 2 gives an overview of the results of the analysis regarding the coded segments of the interviews on emerging conflicts.

The two most frequently mentioned newly arising political conflicts were the behavior of visitors in urban forests and a functional conflict. NGOs and the administration particularly emphasized misconduct of forest visitors that increased during the COVID-19 pandemic.

“What one finds is that parts of the forest are being overrun in a way that is partly out of ignorance, partly out of malice, partly out of sheer negligence.[…] Conservation areas are being entered and rubbish is being left behind” (NA1).

“The conflicts are mainly where people not only walk on the forest paths where it is quite desirable, but leave the paths. Since they disturb the wildlife. Even still at night running through the forest, roaming with artificial lighting. These are simply disturbances for the natural balance, for the animal world there, which have now reached an extent that is critical” (NA3).

Scientists have hardly mentioned this topic.

The functional conflict is emphasized by all stakeholders and perceived as much more serious than a conflict of use. The respondents emphasize that the near-natural forest management applied in the Karlsruhe city forest, i.e., the protective function, conflicts at some points with those seeking relaxation or the increasing pressure of visitors caused by the COVID-19 pandemic.

“If I want to have a near-natural forest and rely on natural dynamics and want to have natural regeneration in it, I need hunting. And if I then have more visitors in the forest and then also have hunting, then the question is: how does that work? The other thing is that we then have increased damage in the crowns of the trees, with older trees, and then of course the danger of falling deadwood, which increases in climate change. There you see the problem again. Cutting thick trees due to safety concerns, […] or leave the old trees standing, keyword habitat trees and old wood groups–that will certainly be an exciting topic” (SP1).

Further, the conflict between the economic and recreational functions in urban forests is highlighted.

“The forest serves not only the recreational function, but it also serves the economic function and many forest visitors, recreation seekers are bothered by this. Many people still have the idea that a recreational forest is only there for those seeking recreation, and that all other functions must be subordinate to it. And that is sometimes difficult. People do show a lot of consideration, but it starts in winter, when the trees are being felled, and the paths are sometimes dirty. The paths are then restored, but many forest visitors are bothered when they walk through the forest with normal shoes and then get dirty shoes. Or they are bothered purely by the sight of machines. So, there is a certain potential for conflict: Recreational use on the one hand, forestry use on the other. So that’s what I would consider a potential conflict of increasing recreational use through corona time” (A1).

In addition to the functional conflict, the scientists see the accessibility of the forest as a newly emerging challenge, particularly with regard to environmental injustice. Furthermore, barriers, such as allergies to certain tree species can decrease the recreational value of the urban forest.

“You have to analyze a city spatially and see: where do people actually have access to the forest? And is the forest evenly distributed? Because if you sometimes look at it in detail, you realize that many cities have a lot of forest when calculated on their urban area […] but if you then look at it at the district or neighborhood level, you often find out: there are certain parts of the city that have very easy access to the forest, so to speak around the corner, and then there are parts of the city where it takes 45 min by bus and train to get to the next forest. This is difficult from a health and environmental justice point of view. There is of unbelievable injustice, because it is simply spatially unfairly distributed” (SP5).

This is hardly or not at all addressed by the other stakeholder groups. However, another critical issue for the administration is the provision of infrastructure for recreation. They emphasize that the current infrastructure for recreation (network of paths, parking lots, benches, garbage cans, signs, information boards, etc.) is not sufficient when there is increased visitor pressure due to the COVID-19 pandemic in the Karlsruhe city forest and leads to conflicts.

### Strengthening climate change adaptation and recreation in CCF during COVID-19

**Fig. 3:**
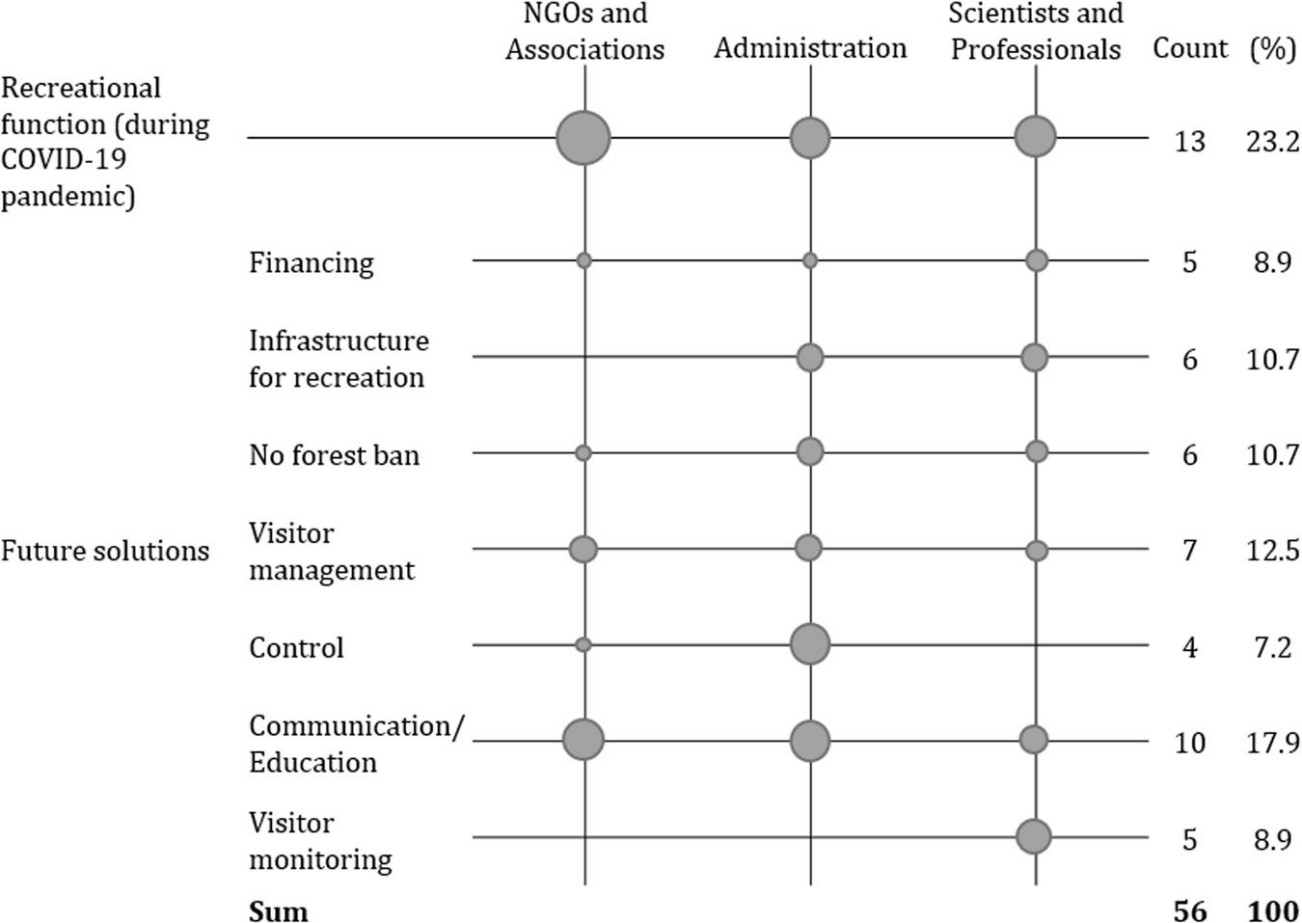
The recreational function of CCF during the COVID-19 pandemic. Boxes represent the average quantity of segmented codes per interviewee per stakeholder group.

The third research question focused on discussing possible future solutions to overcome the before-identified challenges. Figure 3 shows the result of the qualitative analysis regarding the coded segments of the interviews on the recreational function of the urban forest in Karlsruhe during the COVID-19 pandemic. Results in the figure are based on the mentions per code per person within each group of stakeholders. Again, the total number of codes identified is indicated.

In general, no other topic has been raised by the stakeholders as often as the recreational function of the urban forest during the COVID-19 pandemic, counting 13 mentions. All stakeholders throughout the different groups highlighted its importance and acknowledged the new challenges in urban forest management.

When it comes to future solutions to ensure the recreational function of the urban forest, opinions diverge between the different groups of stakeholders. Above all, scientists emphasize the role of visitor monitoring. Stakeholders argue that using participatory concepts to adapt the design of the urban forest to the number of visitors could promote a high level of satisfaction with the urban forest and solves various conflicts.

„One approach would be to implement a kind of social monitoring, so as a city to really think about it continuously, not just how the forest is developing in terms of wood yield on certain tree species, but also to look every few years at how recreational use is developing, identifying where we have to adapt.“ (SP4).

However, the NGOs or the administration do not consider this proposed solution. Scientists and professionals further emphasize the importance of infrastructure for recreation. Arguments include the possibility to reduce littering, conflicts of use, and environmental injustice. The administration supports this idea. NGOs did not mention increased infrastructure as a possible future solution to increase the recreational function of forests.

Members of NGOs and associations emphasize the negative behavior of forest visitors during the COVID-19 pandemic and name the control and sanctions by the public order office, rangers, or forest workers in the Karlsruhe city forest as a possible solution.

“Certain paths are also closed, i.e. where it goes into sensitive areas. However, some people then just climb over them fences and signs. […] One would wish that the city of Karlsruhe, I’ll say it now specifically, with increased controls, draw people’s attention to it, and then do so emphatically, under certain circumstances actually sanction violations, which is practical these days not taking place” (NA1).

However, such sanctions are as well criticized e.g. by members of the administration. According to them, sanctions have low effects and more sustainable ways to raise awareness of the need for forest protection need to be identified.

„In my opinion, further regulations, ordinances and laws, yes you can do that, legislate compulsory leashes and things like that, but then I need someone to control it, and then I’ll come up with bans and fines. I think you can maybe make a little difference with that, but ultimately, it’s about people’s attitudes. […] And there we simply need an awful lot of provided information and awareness raising in public” (A3).

Scientists did mention neither further legislations nor sanctions as an option to reduce conflicts between the urban forests’ climate change adaptation and the need for recreation, nor did they highlight forest visitors’ negative attitudes.

It is essential to all stakeholder groups that there will be no forest ban and that it will remain openly accessible. In addition, strengthening the communication and education of visitors is highlighted by all groups of stakeholders.

“We cannot block a forest, nor can we forbid people to visit certain forest areas at any time of the day or night. In this respect: clarification, creation of acceptance. I think that is the only sensible way” (A1).

“Bans are difficult. I cannot put up prohibition signs everywhere in the forest or something. So the only thing that helps is to try to educate the population better” (NA3).

## Discussion

One of the most discussed aspects of the urban forest’s climate change adaptation was the use of non-native species. Experts and NGOs rather argued for not planting non-native species. They argue that these will best adapt to future climate conditions and that native species are much more valuable for native insects and animals in terms of food source and habitat. Sagoff^40^ identified the same arguments often addressed to illustrate the superiority of native over introduced species. Schoonhoven et al.^41^ and Schirmel et al.^42^ provide evidence that non-native tree species lead to a possible decrease in insect food availability. Further, the Millennium Ecosystem Assessment^43^ identified the increase of invasive species in forests as a driver of its loss of biodiversity. Rejmánek and Richardson^44^ identified broad evidence of invasive tree species’ negative ecological impacts. Sjöman et al.^45^ identified that many city administrations, NGOs, and the media highlight the importance of native species and the need to avoid using non-native species in their praxis-oriented publications and websites.

However, challenges with the definition of non-native species are also highlighted in numerous pieces of literature. Blackburn et al.^46^ highlight that not all introduced species are invasive. Therefore, not all non-native species should be handled as invasive species. In its concept for climate adaptation of the urban forest, the city of Karlsruhe also emphasizes that the genetic diversity of tree species and trees in Karlsruhe’s forests has not yet been investigated. The origin of the tree species designated as native is also ultimately open in many cases, which makes a clear distinction between native and non-native species fundamentally difficult. As an alternative, the list “Overview of native tree species in Germany” published by the Forest Stewardship Council (FSC) certification system is assumed for the definition of site-native tree species in Karlsruhe. In the glossary of the FSC standard, native tree species are defined as tree species of the natural, post-glacial forest development in Germany^8^. Taking that definition as a basis, the city of Karlsruhe aims to limit the share of non-native species in the urban forest to 20 percent^8^.

Studies from other cities, such as Frankfurt am Main, Germany, have shown that non-native species represent a significant component of the urban forest, with a share of almost 60%^47^. Further investigations from Stuttgart, Germany^48^ or the Ruhr area in Germany^49^ support this increasing expansion of non-native tree species in German urban forests. Richardson et al.^50^ and Sjöman et al.^45^ highlight that this trend can be identified in cities worldwide. In this study, stakeholders from the administration highlighted the importance of including non-native species in urban forest management. They argue that these will be needed in arid and hot areas to secure the provision of ecosystem services by the urban forest. The municipal administration of the city of Karlsruhe also emphasizes that numerous native tree species have suffered severe losses in recent years due to climate change and biotic and abiotic stressors. The city administration predicts that these tree species could continue to decline in vitality and competitive strength. In addition, it is to be expected that these tree species will no longer reach their previous life expectancy^8^. The arguments taken up by the administration in this study’s interviews thus support the statements in the city’s climate adaptation plan. There, it is shown that the participation of site-appropriate, non-native tree species is needed as a supplement to maintain sustainable use options for the urban forest. The administration argues that the renunciation of these tree species would reduce the climate resilience of the forests and thus endanger the sustainable safeguarding of all forest functions, at least locally^8^. Conway et al.^51^ argue that using non-native species could reduce the tension between ecosystem service provisioning and ecological integrity in the urban forest. Sjöman et al.^45^ further highlights in their study from northern and central Europe that the number of native species may be very limited in certain regions. They identify a high probability that native species alone will not suffice in those regions to provide the necessary ecosystem services and resilience in the city. The authors, therefore, call for integrating the possibility of using non-native species in regional, national, and international policy documents^45^.

Two recommendations to overcome questions regarding the use of non-native species in urban planning have been highlighted by the stakeholders in the interviews: (i) increased research on the characteristics, chances, and challenges in their use and (ii) increased location-adjusted planning. The Food and Agricultural Organization of the United Nations (FAO) also identifies a need for increased research on tree characteristics, planting opportunities, and the selection of tree species^52^. Reif et al.^53^ in 2011 identified a research gap in the adaptability of non-native tree species to climate change. In 2020, Pötzelsberger et al.^54^ however argue, that extensive research on non-native species and their opportunities and limits for climate change adaptation took place during the last decades. The authors instead call for an uptake in the political and administrative agenda for implementation, where the discussion is relatively new yet. Sjöman et al.^45^ call for regional perspectives in this research and for an enhancement of locally adjusted planning. The individual regional context must be considered when selecting tree species. Furthermore, decisions on the use of non-native species need to be taken regionally as their need and suitability highly depend on the conditions and locally available richness of regional tree flora.

Challenges of financing the urban forest’s adaptation to climate change have been highlighted especially by the group of NGOs and Associations and by Scientists and Professionals. Both criticize the politics for investing too little in the urban forests. According to Ottitsch et al.^55^, the consequences of climate change are neglected by politicians because the effects are still too far in the future. Even though this attitude is currently changing, the issue of funding can only be solved via secure funds, communication between stakeholders and the population, and a public discourse on the future challenges of the urban forest.

The need to strengthen the urban forest’s recreational function during the COVID-19 pandemic has been the most often discussed point during the stakeholder interviews of this study. This is supported by numerous authors, such as Xie et al.^56^ and Weinbrenner et al.^57^, who highlight the urban forest’s importance to meeting and socially interacting, which has been limited in other locations during the pandemic. Further, Beckmann-Wübbelt et al.^15^ analyzed the urban forest’s vital role for the mental and physical well-being of the urban population. A visitor boom in urban forests around the world reveals the importance of the urban forest’s recreational function^14,15,27^. In addition, a new set of visitors has been identified by Derks et al.^27^ and Beery et al.^58^. This new composition of forest visitors challenge urban forest managers and forestry professionals in regard to safeguarding the recreational function for all^27^. The results of this study show that most stakeholders perceive the misconduct of forest visitors as crucial for the emerging conflicts between climate change adaptation and recreation in CCF management.

All interviewed stakeholders called for increased education of the public on how to behave in the forest by showing more respect for nature. Similarly, Bernsasconi et al.^59^ suggested that communication between forest offices and education for recreationists helps to improve forest visitor behavior and may support strengthening its recreational function. Studies by Kuhar et al.^60^ and Padua^61^ on the effects of environmental education in schools on behavior in forest nature reserves have yielded different results. Padua^61^ found the environmental program’s positive effects on behavior inside forests. On the other hand, Kuhar et al.^60^ could not attribute behavioral changes to environmental education. However, numerous authors, such as Beery et al.^58^ call for more guidance for new users in the urban forest to enhance the enlightenment and creation of acceptance. In response to the limitations of environmental education identified by Kuhar et al.^60^ and Beery et al.^58^ highlight that new formats and platforms for outreach must be considered. Besides that, the authors call for infrastructure that can cope with the diversity of recreational activities in the forest^58^.

The scientists and professionals only mention the approach of visitor monitoring to strengthen the recreational function of the urban forest. The literature suggests that more approaches may help increasing a forest’s recreational value. For example, Hunziker et al.^62^ define successful urban forest management as the knowledge of recreation desires, actual recreation, and satisfaction or criticism of the recreationists. Studies such as from the *Forstliche Versuchs- und Forschungsanstalt Baden-Württemberg* (FVA)^63^ and Beckmann-Wübbelt et al.^15^ created use maps through citizen participation. According to Gerstenberg et al.^64^ such maps may help foresters guide visitors and develop recreation infrastructure more efficiently. Beery et al.^58^ highlighted that in Sweden, efforts to channel recreationists to less frequented sites to reduce congestion had been taken during the pandemic. Thereby conflicts between different user groups could be prevented. According to the scientists and professionals that participated in our study, a similar approach could help to strengthen the recreational function of the urban forest in Karlsruhe as well.

The increased number of visitors during the COVID-19 pandemic did not only raise challenges in safeguarding the recreational function of the urban forest. It also revealed conflicts between the recreational and other functions. Especially, functional conflicts between recreation and biodiversity protection, as well as recreation and the economic utilization function, have been highlighted in the interviews. Similarly, Cole and Landres^65^ emphasized that recreation is one of the main threats to biodiversity conversation. Pouwels et al.^66^ found positive correlations between an increased number of visitors and a decrease in bird populations. Likewise, human recreation was identified as a contributing factor to the loss of species by Czech et al.^67^. The interviewed stakeholders highlighted that leaving dead stems in the urban forest in order to promote habitat for a wide range of animals, conflicts with the need to assure safety for visitors’ recreation. While in protected nature parks, visitors highly value biodiversity^68^, a study by Korpilo et al.^69^ in the urban forest in Finland revealed no correlation between biodiversity features and appreciation by visitors. Thus, the authors suggest, that in urban forests human/social and ecological demands may be diverging.

Besides conflicts between the urban forests recreational and biodiversity protection function, increasing conflicts of the economic utilization and recreational function have been highlighted by the different stakeholder groups. It is highlighted that urban forest visitors are displeased with heavy machineries such as harvesters and signs of heavy human intervention. Similarly, Gundersen and Frivold^70^ found that recreationists in the urban forest prefer a close-to-natural appearance of it. Therefore, forest management that prioritizes wood production often conflicts with the recreational function of the urban forest^71,72^. As this study reveals, such conflicts increased during the COVID-19 pandemic. Eggers et al.^72^ highlight that municipalities play an important role in protecting the recreational value of the urban forest. However, this requires financial resources, which are often scarce. In this study, stakeholders from NGOs and associations as well as scientists and experts emphasized that the recreational function and the climate change adaptation of the urban forest need to be prioritized. However, facing this approach’s financial challenges, all stakeholders agree that the utility function in the urban forest cannot be completely disregarded.

To minimize functional conflicts in urban forest management during times of climate change and an increase in visitors due to COVID-19, visitor guidance has been highlighted as an effective tool. If certain areas may be prioritized for biodiversity conversation or wood production, visitors can be guided to other parts of the urban forest that are dedicated to recreational use. Such a strategy is supported by several authors such as Eggers et al.^72^, Juutinen et al.^68^ and Pouwels et al.^66^. To evaluate possible spatial prioritization of urban forest use, Eggers et al.^72^ developed a forest decision support system that integrates the urban forest’s economic and recreational functions. Results from application tests of the system show that enhancing the recreational function of the urban forest can be completed without large economic drawbacks. However, the authors did not consider the possible consequences for its ecological functions^72^. Pouwels et al.^66^ and Korpilo et al.^69^ further highlight the importance of mapping ecological and recreational features and use of the urban forest to prioritize areas for specific functions. According to Korpilo et al.^69^, formulating clear recommendations for practice that include interdisciplinary findings regarding a large number of urban forest functions is difficult and often hinders its implementation. Spatially explicit data could help overcome such difficulties and support urban forests managers in their decision-making.

Although the law allows the forest administration to require certain rules of conduct from forest visitors, and although tools to prioritize areas for recreational function are being developed, there are further challenges associated with visitor guiding. We found that it is very important for many forest visitors to be able to move freely in the forest. Therefore, guidance cannot be too strict. Juutinen et al.^68^ emphasized visitor guiding to create spaces for biodiversity conversation, highlight that a high concentration of visitors in some parts of the urban forest may in turn lead to a decrease of the visitors’ satisfaction and the recreational value of the area. More research is needed on how visitor guidance affects the recreational and biodiversity protection function of the urban forest.

All stakeholders have explicitly highlighted that locking the forest for visitors cannot be considered a solution to functional conflicts.

Even though the study results provide a deep insight into challenges and needs regarding the provision of climate change adaptation measures and recreation in urban forests, some limitations of the study need to be considered. While community decision-makers, NGOs, and researchers were interviewed for this study, private companies and global associations such as the FAO or United Nations Human Settlement Programme (UN-Habitat) and the WHO, were not solicited. However, further research should include these experts as suggested by Salbitano et al.^1^. Concerning the increasing demand for the recreational function of the Karlsruhe city forest, local tourism associations and companies were requested for interview. Those experts disagreed with being interviewed. Private companies from the timber industry were not approached, as the forestry offices of corporate and state forests take care of timber sales.

Future research and monitoring need to further investigate and evaluate the long-term impact of the COVID-19 pandemic on the recreational uses of urban forests. Measures to adapt to climate change impacts also need to be further explored, as climate change impacts cannot yet be assessed with certainty, and policy opinions may vary depending on current circumstances and new research findings.

## Methods

### Study area

**Fig. 4:**
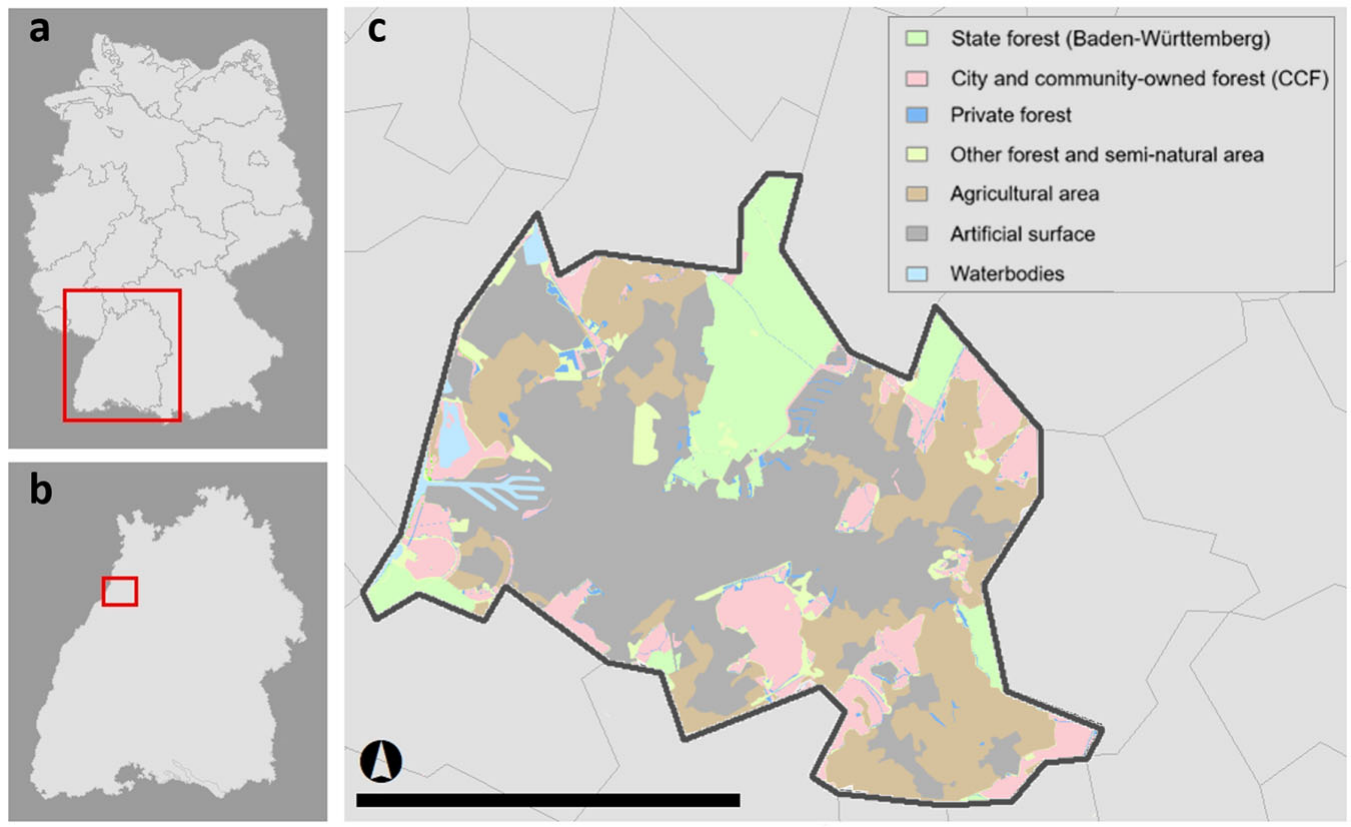
Map of Karlsruhe by land use and forest ownership types. Own illustration based on LFV^81^. Scale bar in **c** represents 10 kilometers.

The study area, the city of Karlsruhe, is located in the southwestern part of Germany in the state of Baden-Württemberg. With its approx. 308,000 inhabitants in 2020, it is the second largest city within the state after the state capital city Stuttgart. As the city was originally built into the forest, Karlsruhe still bears a relatively large forest area of 4446 ha (as of 2020), representing one-quarter of the city’s total area^73^. With an area of 2,250 hectares, about half of the forest is municipal and community-owned forest (CCF) which is managed by the city administration^6^. Figure 4 shows a map of Karlsruhe by land use and forest ownership types. As in many other cities, climate change poses significant challenges to the CCF in Karlsruhe. During the last few years, trees have been suffering irreversible damage, which threatens to increase due to climate change^8^. At the same time, it has been revealed that the urban forest played a crucial role in the physical and mental well-being of the urban population during the COVID-19 pandemic^15^. The city of Karlsruhe, therefore, represents a valuable case study for this research.

### Stakeholders’ selection

To identify the stakeholders, we used the concepts of Salbitano et al.^1^ and Ottitsch and Krott^55^. The stakeholders were divided into NGOs, associations, administration, and scientists and professionals (Table 2).

**Table 2:** Classification of the interviewed stakeholders according to the identified groups.

Stakeholder group	NGOs and associations	Administration	Scientists and professionals
Institution	Nature conservation organization, citizens’ initiative	Administration of state forests, administration of municipal forests, city administration, regional council	Research on climate change, health effect of urban woods, forest policy science, regional planning
Identification code	NA	A	SP
Number of stakeholders/interviewees	4	4	8

*NA* NGOs and associations, *A* administration, *SP* scientists and professionals.

As suggested in the literature, the group of NGOs and associations includes nature conservation associations and citizens’ associations and initiatives from Karlsruhe^1,55^. The administration includes the municipal council, the state forest administration, the Karlsruhe forest administration, the forest districts, and related offices of the city administration. These simultaneously represent the forest owners.

Lastly, the group of scientists and professionals includes experts from the fields of climate change research and human health research, as well as from the forest policy and spatial planning sector.

### Interview guidelines and implementation

According to Meuser and Nagel^74^, the systematized, semi-structured interview was chosen as the basis methodology for the stakeholder interviews. This ensures the assessment of personal perceptions and experiences from a small group of participants and an understanding of the complex reality^75^. A guide was developed to ensure the coherency of all interviews^76^. The guide was grouped into thematic blocks, and a sensible order of the blocks was determined, which was adhered to during all interviews^77^. In the first block, general questions were asked to contextualize the interview and to start the conservation quickly. These questions included questions on the professional background and personal experiences with the urban forest. After that, three blocks of thematic questions followed:

- Firstly, the stakeholders’ opinion on the current climate change adaptation plan (CAP) for the urban forest in Karlsruhe was assessed. This plan was published by the city of Karlsruhe in 2020 and includes resolutions on various aspects such as tree species selection in climate change that should be followed in future CCF management in Karlsruhe^8^.
- Secondly, the influence of the COVID-19 pandemic on urban forest management was discussed.
- Lastly, questions on arising political conflicts and possible solutions for climate change adaptation and the increased demand for recreational functions of the CCF have been posed.

In addition to the main questions, possible follow-up questions were developed to ensure the fluency of the conversation while still leaving room for spontaneously upcoming topics^76^. At the end of the interviews, stakeholders had the opportunity to add important aspects that had been missing during the interview. The interview guide was adjusted and reformulated according to the pre-testing results before conducting the stakeholder interviews. A translation of the final interview guide can be found in the Supplementary Methods.

We had requested 54 potential stakeholders, of which 16 agreed to be interviewed in the summer of 2021. The interviews lasted 25 min on average, with the shortest recording 12 min (with NA3) and the longest 43 min (with NA1). Interviews were conducted in the German language. Due to travel and contact restrictions of the pandemic situation, interviews were conducted via the Zoom video conferencing tool. The program provided a recording function that saved the audio track separately. A privacy notice was emailed to the experts prior to the interviews. Additionally, at the beginning of the interview, the interviewer explained the rules on data protection and assured to evaluate the interviews anonymously. All interview partners approved the audio recording of the interview.

### Evaluation and analysis

The analysis of the interviews contained four parts: (1) familiarization with the data, which included a first scan of all transcripts, (2) two rounds of deductive and inductive coding, (3) generation of themes and interpretation of interrelations, and (4) compilation and visualization of results. Further, the homogeneity of arguments within and between the different stakeholder groups was analyzed. All analysis were implemented in MAXQDA^78^.

The transcription of the stakeholders’ interviews was applied according to Bogner et al.^77^ rules for smoothing the spoken word. This implies an abstraction from the vivid, spoken language with many nuances, stresses, and a specific speech style in favor of what can be recorded in written language. Anonymization of the interviews was performed according to Ebel and Meyermann^79^, who provide a practical guide to identifying personal data, contextual information, respondent characteristics, and sensitive information, and provide options to delete, generalize, or modify them so that individual interviewees cannot be identified.

According to Kuckartz and Rädiker^80^, a coding system was developed deductively based on the theoretical background and the interview guide to group the stakeholder’s arguments and perceptions. This initial codebook was applied in a first round of exploration. By analyzing the interviews one by one, we developed inductive codes which were subsequently added to the codebook. After that, we conducted a second run of analysis to validate the codebook’s integrity.

Finally, the stakeholders’ statements were interpreted qualitatively and analyzed concerning the given framework^77^. The analysis was based on content-structuring qualitative content analysis according to Kuckartz and Rädiker^80^. To facilitate the qualitative content analysis, summary tables were created using the Summary Grid function, which in the first step produces coded segments, i.e., text passages from the interviews. The summaries per interview are subsequently again summarized in a results table per code. The results table serves the researcher to get an orderly overview of the statements^78^. We formulated overarching themes based on the interview structure and the guiding research questions and grouped the codes accordingly. Finally, the codes were examined individually to ensure that the data grouped under them were meaningful, related, and followed the same argument. We further tested the data for overlaps between the arguments of different codes. The qualitative analysis led to further differentiation of the codebook where some codes were divided in sub codes to more adequately interpret the stakeholder’s arguments. In the final codebook, the applied categories are defined, an explanation is given, and a distinction is made as to whether they emerged deductively or inductively during the interviews^80^. The most important codes are represented and explained in Supplementary Table 1.

Additionally, to the qualitative analysis, we quantitatively analyzed and visualized the data in MAXQDA to identify the most often highlighted arguments by stakeholders. A cross tabulation^80^ was created to show the relationship of a group to each theme and to compare the opinions of the stakeholders. In the MAXQDA program, the variable ‘Stakeholder Group’, i.e., NGOs and Associations, Administration and Scientists and Professionals, was set. To compare the stakeholder groups that contain different numbers of stakeholders, the codes mentioned were normalized by indicating the average number of mentions per person.

Interview similarity within and between the different stakeholder groups was calculated quantitatively in MAXQDA using a document similarity analysis^78^. The function uses the following formula to calculate the percentage match of two interview transcriptions where existence and non-existence are counted as a match: (a + d)/(a + b + c + d). Thereby *a* is the number of codes that are identical in both transcriptions, *b* is the number of codes that do not exist in both transcriptions, and *c* and *d* represent the number of codes that exist in only one of the two interview transcripts^78^. The mean of the occurrences of the code segments in the interviews was calculated to compare the stakeholders within and between the groups. The similarity analysis gave an insight into the homogeneity of the answers of several experts of the same stakeholder group and allows for evaluating the stakeholders’ grouping.

### Reporting summary

Further information on research design is available in the Nature Research Reporting Summary linked to this article.

## Supplementary information

Supplementary Material

Reporting Summary

Dataset 1

## Data Availability

The data used in this study are transcripts from audio recordings of the interviews of stakeholders. The anonymous transcripts were uploaded to Figshare open-access repository with the DOI 10.6084/m9.figshare.22099541. The link is https://figshare.com/articles/dataset/Interview_transcripts_for_the_article_Climate_change_adaptation_measures_conflicted_with_the_recreational_demands_on_city_forests_during_COVID-19_pandemic_/22099541.
