# Supplementary material for: Climate change adaptation measures conflicted with the recreational demands on city forests during COVID-19 pandemic
Source: NPJ Urban Sustain. 2023 Mar 15;3(1):17. doi: 10.1038/s42949-023-00096-y (PMC10016162; doi:10.1038/s42949-023-00096-y)
Supplement: Supplementary file 1 — Supplementary Material [file 42949_2023_96_MOESM1_ESM.pdf]

SUPPLEMENTARY INFORMATION: Climate change adaptation measures conflicted with the recreational demands on city forests during COVID-19 pandemic

Angela Beckmann-Wübbelt [1], Lynn Türk [1], Iulia Almeida [1], Annika Fricke [1], Metodi Sotirov [2], Somidh Saha [1, 3]*

[1] Institute for Technology Assessment and Systems Analysis (ITAS), Karlsruhe Institute of Technology, Karlstr. 11, 76133 Karlsruhe, Germany

[2] Chair of Forest and Environmental Policy, University of Freiburg, Tennenbacherstr. 4, 79106 Freiburg im Breisgau, Germany

[3] Institute for Geography and Geoecology (IfGG), Karlsruhe Institute of Technology, Kaiserstr. 12, 76131 Karlsruhe, Germany

*corresponding author: somidh.saha@kit.edu

SUPPLEMENTARY METHODS

Interview Guideline (Translation from German)

Numerical code respondent:
Interview date:
Duration of the interview:
Stakeholder (mark the appropriate)

NGOs and Associations (NA)	1. Nature conservation organization<br>2. Citizens'''' initiative
Administration (A)	1. Administration of state forest<br>2. Administration of municipal forest<br>3. City administration<br>4. Regional council
Scientists and Professionals (SP)	1. Climate change research<br>2. Health effect of urban woods<br>3. Forest policy science<br>4. Regional planning

Interview guideline on "Future political actions of the city of Karlsruhe for the preservation and improvement of the city forests in view of climate change and the COVID-19 pandemic".

<u>Welcoming</u>
(Gratitude, consent form signed, anonymity in the text of the master's thesis, duration, my person, subject of the master's thesis,..)

<u>Topic block 1: about the person: background, mood, socialization</u>
Expectation:
- *Loosening up the situation*
- *Getting into the conversation*
- *Assessing whether the person will talk a lot on his or her own or whether a lot of questions need to be asked*

1. What is the field of work of the company/organization/office where you work?
2. What are your professional responsibilities in the company/organization/office?
3. Have you ever visited one of the city forests of Karlsruhe?
    - If yes, which one, how often, what do you appreciate about this forest?
    - If no, what do you generally appreciate about the forest you visit?
4. What does nature conservation mean to you / what does near-natural forest management mean to you? (If forestry background)

Topic block 2: Process and interpretive knowledge on the current climate protection concept for the urban forest in Karlsruhe.

Expectations:

- Urban forests important role against climate change impacts Promotion important.

- Heat tolerance: tree species selection (native vs. non-native)

- Land use conflict: fragmentation due to urbanization weakens biodiversity and thus important functions of forests

1. This study is about possible political actions to better manage the consequences of climate change. Are you familiar with the current climate protection concept for the urban forest in Karlsruhe?
    - If no, explanation: near-natural management with fulfillment of protection (biodiversity), social (recreation), utility (hunting and timber) and educational functions; legally protected areas; PEFC and ANW guidelines; future tree population climate resilient) (reference "Concept for Climate Adaptation for the City Forest of Karlsruhe dated June 30, 2020 of the City and Forestry Office).
    - If yes, how do you evaluate the current climate protection concept regarding the city forests of Karlsruhe? (Question about weak points and strengths of the current climate policy).

2. What conflicts/ contradictions do you see in the current urban forest management with regard to the ever increasing climate change impacts and at the same time maintaining the quality of the urban forests? (If weaknesses/conflicts not explained enough before).
   Possible follow-up on:
    - Effective tree species selection with limited introduction of non-native tree species.
    - Financing of near-natural management with simultaneous timber harvesting losses.

3. What policy instruments, structural changes, guidelines are needed for the future to protect Karlsruhe's urban forests against climate change?

Topic Block 3: Process & interpretive knowledge of the COVID-19 pandemic and urban forest management.

Expectations:

- Cities more vulnerable to pandemics = forests more important

- Infrastructure / environmental justice for all urbanites.

- Nature-based management and duty of care

- Land use conflict (more forest land and more population at the same time.

1. Research has shown that forest visitation is very important to the physical and mental health of urban populations and that more people went to the forest during the COVID-19 pandemic.
    - What conflicts do they see in the current urban forest management with regard to the COVID-19 pandemic, especially if such situations should possibly occur more frequently in the future? Also using Karlsruhe as an example.

- How can the environmental justice and recreational functions of urban forests be maintained in the face of further pandemics and increasing urbanization?

2. What consequences do you draw from the COVID-19 pandemic with regard to the management of urban forests? Future
   - What policy tools, structural changes, guidelines are needed for the future so that urban forests in Karlsruhe can fulfill their role of sports and recreation facility in situations like the COVID-19 pandemic?
   - What policy changes did the COVID-19 pandemic bring that should continue to be maintained /or deepened?

<u>Topic block 4: Political conflicts</u>

Expectations:
- Maintaining quality in conflict with climate change and recreation factor.
- Pursue Agenda 2030 (SDG): align global goals/networking.
- ownership regulations

1. What does quality of forest areas around Karlsruhe mean to you?
   - In what criteria would you measure the quality of the urban forest?

2. How can global problems such as climate change and the COVID-19 pandemic best be steered politically at the lowest/local level?
   - Would centralization of governance be better, for faster implementation on global problems, or the other way around, why?

3. After the timber cartel case, the 2016 coalition agreement justifies the transfer of the state forest, which also takes up areas of the city forest, to ForstBW, an institution under public law, on the grounds that this will better promote climate goals, recreational value and other forest functions. What is your opinion on this?

<u>Conclusion</u>

1. Is there anything important you would like to tell me in connection with the interview? (Maybe anything you thought of during the interview and forgot to mention?)

2. Who could I ask for further research from? Do you have any helpful literature that you can recommend or refer me to?

SUPPLEMENTARY TABLE

The most important codes referred to in the paper's results and discussion section are presented and explained in Supplementary Table 1.

Supplementary Table 1: Codebook includes the most essential deductive and inductive codes and explanations identified in the study.

**Theme**<br>**Code**	**Category**	**Explanation**
Climate change adaptation	deductive	Contains areas of conflict and solutions to the topic of climate change adaptation in the forests of Karlsruhe from a forest policy perspective.
Causes of current political conflicts	deductive	In the interview, the political areas of conflict that arise from the stakeholders' perspective in the area of climate change adaptation of the forests in Karlsruhe are named and explained in the following subcodes. In addition, the problems that arise from climate change and thus lead to political conflict areas are named.
Use function in focus	inductive	Political decisions are guided by the fact that the useful function, i.e. the economic use of the forest for climate change adaptation, is preferred.
Financing	deductive	Suggestions and opinions on how the measures for financing the climate change adaptation strategy should be carried out.
Clear cut areas	inductive	The existence or planned processes of bare areas caused by tree dieback or wood removal in the Karlsruhe city forest, which could have a negative (subcode "contra") or positive (subcode "pro") impact on the consequences of climate change.
Pro	inductive	Arguments in favor of allowing bare areas to develop as a result of tree death or wood extraction in the Karlsruhe city forest, which could have a positive effect on the consequences of climate change.
Contra (afforesta-tion)	inductive	Arguments for the reforestation of bare areas that are caused by tree death or timber extraction in the Karlsruhe city forest. Arguments that highlight a negative impact of clear cuts on the consequences of climate change.
Urbanization/ Urban design	deductive	A special feature of the city forest is its proximity and thus its function for the city. Thus there is a relationship between the design of the city and the city forest. However, explanations are mostly general, rarely related to Karlsruhe.

Tree species selection	deductive	Probably the biggest area of conflict in climate change adaptation is the selection of tree species, which harbors different opinions when introducing non-native tree species. While the subcodes (cons: non-native tree species and pro: non-native tree species) show the reasoning of the opinions, this conflict is defined here, and the questions about it are discussed.
Contra: non-native tree species	deductive	Arguments against the introduction of non-native tree species or positive properties of native tree species.
Pro: non-native tree species	deductive	Arguments, conditions, and explanations for the introduction of non-native tree species.
Future solutions	deductive	In the interview, the possibilities of future solutions to the conflict areas are discussed on the topic of climate change adaptation of the forests in Karlsruhe and explained in the subcodes.
Contra process protection	inductive	Process protection, i.e., no intervention in the ecological processes of the Karlsruhe city forest, is not seen as a solution to climate change adaptation.
Location adjusted	inductive	Arguments that highlight the importance of site-specific conditions and functions of the forest in the field of climate change adaptation.
Hunting	inductive	Hunting must be permitted under certain regulations to prevent excessive browsing by game in the natural regeneration defined in near-natural forest management.
Use function as a secondary focus	inductive	The solution to the conflict areas in the area of climate change adaptation of the city forests of Karlsruhe lies in the prioritization of the functions to be fulfilled, whereby the financing, especially through forestry, has to take a back seat.
Mixed forest/ biodiversity	inductive	The solution to the areas of conflict in the area of climate change adaptation in the city forests of Karlsruhe lies in increasing the biodiversity of the forest stock through a species-rich mixed forest.
Communication	deductive	The solution to the conflict areas in the area of climate change adaptation of the city forests of Karlsruhe lies in the communication and agreement in the political process of finding a solution.
Research	deductive	The solution to the conflict areas, especially in the choice of tree species, in the area of climate change adaptation of the city forests of Karlsruhe lies in research and years of experience, as well as the incorporation of results from research work on climate change consequences and scenarios.

Recreational function (during COVID-19 pandemic)	deductive	Contains areas of conflict and approaches to solving the topic of the recreational function of the forests of Karlsruhe in times of the COVID-19 pandemic from a forest policy perspective.
Future solutions	deductive	In the interview, the possibilities of future solutions to the conflict areas, which are discussed in the recreational function of the forests in Karlsruhe during the COVID-19 pandemic, are explained in the subcodes.
Financing	inductive	Financial support for the recreation function of the Karlsruhe city forest to manage the visitor pressure in times of the COVID-19 pandemic, e.g., through public funds that provide the infrastructure for recreation.
Infrastructure for recreation	inductive	Arguments that highlight the importance of infrastructure for recreation as a solution to reduce littering, conflicts of use, and environmental injustice.
No bans	inductive	Arguments that highlight the impotance of accessibility of the urban forest for all. Arguments that reject ideas of (partly) closing the forest for visitors.
Visitor management	inductive	In order to solve conflicts of use and function, in particular, the adaptation or the creation of new paths for visitor guidance in the Karlsruhe city forest are being discussed.
Control	inductive	In order to solve the negative behavior of forest visitors during the COVID-19 pandemic and beyond, control and sanctions by the public order office, rangers, or forest workers in the Karlsruhe city forest are being discussed.
Communication/ Education	deductive	Arguments that highlight the importance of communication between the stakeholders, as well as between the forest administration and the population. Arguments that highlight a need for transparent politics as well as education of the population regarding the city forest.
Visitor monitoring	deductive	Surveys and participatory political processes for the design of the Karlsruhe city forest promote the satisfaction of forest visitors and can solve various conflict fields.
Current conflicts	deductive	Arguments that highlight arising conflicts in safeguarding the recreational function of the forests in Karlsruhe in times of the COVID-19 pandemic.
Infrastructure for recreation	deductive	The current infrastructure for recreation (network of paths, parking lots, benches, rubbish bins, signs, information boards, etc.) is insufficient, especially when there is increased visitor pressure due to the COVID-19 pandemic in the Karlsruhe city forest and this leads to conflicts.

Behavior of forest visitors	deductive	The increased conflict that exists in times of the COVID-19 pandemic among those seeking recreation. The new or increasing misconduct of the forest visitors and the causes are discussed.
Accessibility of the forest for all	deductive	Environmental injustice can be discussed as a conflict in the city forest, but it probably occurs less in the Karlsruhe city forest, which is why it is addressed more generally. In addition to accessibility, this also includes a wide variety of barriers, such as allergies to certain tree species.
Usage conflicts	deductive	The conflict that existed before the COVID-19 pandemic may have intensified. Conflicts of use between forest visitors, i.e., those who use the recreational function in the forest but visit the forest for various reasons.
Function conflicts	deductive	The near-natural forest management applied in the Karlsruhe city forest, i.e. the protective function, conflicts at some points with those seeking relaxation or the increasing pressure of visitors caused by the COVID-19 pandemic. The points of conflict are discussed.
