# Supplementary material for: Climate change adaptation measures conflicted with the recreational demands on city forests during COVID-19 pandemic
Source: NPJ Urban Sustain. 2023 Mar 15;3(1):17. doi: 10.1038/s42949-023-00096-y (PMC10016162; doi:10.1038/s42949-023-00096-y)
Supplement: Supplementary file 2 — Reporting Summary [file 42949_2023_96_MOESM2_ESM.pdf]

nature portfolio

Corresponding author(s): Somidh Saha

Last updated by author(s): Feb 15, 2023

Reporting Summary

Nature Portfolio wishes to improve the reproducibility of the work that we publish. This form provides structure for consistency and transparency in reporting. For further information on Nature Portfolio policies, see our Editorial Policies and the Editorial Policy Checklist.

Statistics

For all statistical analyses, confirm that the following items are present in the figure legend, table legend, main text, or Methods section.

n/a	Confirmed	
☐	☒	The exact sample size (*n*) for each experimental group/condition, given as a discrete number and unit of measurement
☐	☒	A statement on whether measurements were taken from distinct samples or whether the same sample was measured repeatedly
☒	☐	The statistical test(s) used AND whether they are one- or two-sided<br>*Only common tests should be described solely by name; describe more complex techniques in the Methods section.*
☒	☐	A description of all covariates tested
☒	☐	A description of any assumptions or corrections, such as tests of normality and adjustment for multiple comparisons
☒	☐	A full description of the statistical parameters including central tendency (e.g. means) or other basic estimates (e.g. regression coefficient) AND variation (e.g. standard deviation) or associated estimates of uncertainty (e.g. confidence intervals)
☒	☐	For null hypothesis testing, the test statistic (e.g. *F*, *t*, *r*) with confidence intervals, effect sizes, degrees of freedom and *P* value noted<br>*Give P values as exact values whenever suitable.*
☒	☐	For Bayesian analysis, information on the choice of priors and Markov chain Monte Carlo settings
☒	☐	For hierarchical and complex designs, identification of the appropriate level for tests and full reporting of outcomes
☒	☐	Estimates of effect sizes (e.g. Cohen's *d*, Pearson's *r*), indicating how they were calculated

Our web collection on statistics for biologists contains articles on many of the points above.

Software and code

Policy information about availability of computer code

Data collection	Stakeholders interview were carried out online and recorded in Zoom. All EU and German guidelines on privacy lawa were followed which were also described in Method section.
Data analysis	Data were analysed in MAXQDA software.

For manuscripts utilizing custom algorithms or software that are central to the research but not yet described in published literature, software must be made available to editors and reviewers. We strongly encourage code deposition in a community repository (e.g. GitHub). See the Nature Portfolio guidelines for submitting code & software for further information.

Data

Policy information about availability of data

All manuscripts must include a data availability statement. This statement should provide the following information, where applicable:

- Accession codes, unique identifiers, or web links for publicly available datasets
- A description of any restrictions on data availability
- For clinical datasets or third party data, please ensure that the statement adheres to our policy

The primary data, which were the transcripts of stakeholders interview, were uploaded in Figshare. The link has been provided in the manuscript.

Human research participants

Policy information about studies involving human research participants and Sex and Gender in Research.

Reporting on sex and gender	Not applicable
Population characteristics	Not applicable
Recruitment	Not applicable
Ethics oversight	Not applicable

Note that full information on the approval of the study protocol must also be provided in the manuscript.

Field-specific reporting

Please select the one below that is the best fit for your research. If you are not sure, read the appropriate sections before making your selection.

☐ Life sciences ☒ Behavioural & social sciences ☐ Ecological, evolutionary & environmental sciences

For a reference copy of the document with all sections, see nature.com/documents/nr-reporting-summary-flat.pdf

Behavioural & social sciences study design

All studies must disclose on these points even when the disclosure is negative.

Study description	Mixed method case study
Research sample	16 stakeholders or respondents from southwest Germany
Sampling strategy	We had requested 54 potential stakeholders, of which 16 agreed to be interviewed in the summer of 2021. The interviews lasted 25 minutes on average, with the shortest recording 12 minutes and the longest 43 minutes. Interviews were conducted in the German language. Due to travel and contact restrictions of the pandemic situation, interviews were conducted via the Zoom video conferencing tool. The program provides a recording function that saves the audio track separately. A privacy notice was emailed to the experts prior to the interviews. Additionally, at the beginning of the interview, the interviewer explained the rules on data protection and assured to evaluate the interviews anonymously. All interview partners approved the audio recording of the interview.
Data collection	Interviews of 16 respondents were done online and recorded in Zoom. The second author (Lynn Türk) did all the interviews and no one was present between the interviewee and interviewer. The second author was familiar with research questions of the project.
Timing	The interviews were recorded between March 2021 and August 2021.
Data exclusions	We did not exclude any respondents or data.
Non-participation	We requested 54 potential respondents and 16 had agreed to be interviewed
Randomization	To identify the stakeholders, we used the concepts of Salbitano et al. (2016) and Ottitsch and Krott (2010). The stakeholders were divided into NGOs, associations, administration, and scientists and professionals (see Table 1 in the manuscript).<br>As suggested in the literature, the group of NGOs and associations includes nature conservation associations and citizens' associations and initiatives from Karlsruhe (Ottitsch and Krott 2010; Salbitano et al. 2016). The administration includes the municipal council, the state forest administration, the Karlsruhe forest administration, the forest districts, and related offices of the city administration. These simultaneously represent the forest owners. Lastly, the group of scientists and professionals includes experts from the fields of climate change research and human health research, as well as from the forest policy and spatial planning sector.

Reporting for specific materials, systems and methods

We require information from authors about some types of materials, experimental systems and methods used in many studies. Here, indicate whether each material, system or method listed is relevant to your study. If you are not sure if a list item applies to your research, read the appropriate section before selecting a response.

Materials & experimental systems

n/a	Involved in the study
☒	☐ Antibodies
☒	☐ Eukaryotic cell lines
☒	☐ Palaeontology and archaeology
☒	☐ Animals and other organisms
☒	☐ Clinical data
☒	☐ Dual use research of concern

Methods

n/a	Involved in the study
☒	☐ ChIP-seq
☒	☐ Flow cytometry
☒	☐ MRI-based neuroimaging
