# Supplementary material for: Climate change adaptation measures conflicted with the recreational demands on city forests during COVID-19 pandemic
Source: NPJ Urban Sustain. 2023 Mar 15;3(1):17. doi: 10.1038/s42949-023-00096-y (PMC10016162; doi:10.1038/s42949-023-00096-y)
Supplement: Supplementary file 3 — Dataset 1 [file 42949_2023_96_MOESM3_ESM.pdf]

TRANSCRIPTS OF INTERVIEWS: Climate change adaptation measures conflicted with the recreational demands on city forests during COVID-19 pandemic

Angela Beckmann-Wübbelt [1], Lynn Türk [1], Iulia Almeida [1], Annika Fricke [1], Metodi Sotirov [2], Somidh Saha [1, 3]*

[1] Institute for Technology Assessment and Systems Analysis (ITAS), Karlsruhe Institute of Technology, Karlstr. 11, 76133 Karlsruhe, Germany

[2] Chair of Forest and Environmental Policy, University of Freiburg, Tennenbacherstr. 4, 79106 Freiburg im Breisgau, Germany

[3] Institute for Geography and Geoecology (IfGG), Karlsruhe Institute of Technology, Kaiserstr. 12, 76131 Karlsruhe, Germany

*corresponding author: somidh.saha@kit.edu

Transcripts of interviews

Note: The data used in this study are transcripts from audio recordings of the interviews of stakeholders. The interviews and analysis for this study have been conducted in German language. Therefore, the below presented transcripts of the interviews are as well in German.

NA1

1	**NA1 (43:04.3 Minuten)**
2	**LT:** Was haben Sie denn für einen Bezug zum Karlsruher Stadtwald?
3	**NA1:** Ja gut, durch meinen Wohnort, oft im Stadtwald unterwegs. Von der Rheinaue, den letzten Resten des Auenwaldes bis hoch oder sagen wir mal über die Kiesrücken also den Hardtwald, die Kinzig-Murg-Rinne. Da gibt es einige auch spezifische Wälder, die geprägt sind durch einen höheren Grundwasserstand. Dann die Hangwälder am Rande der Rheinebene, die nach oben gehen. Und natürlich auf der Hochfläche, da wo die Bergdörfer sind, also Stupferich, Hohenwettersbach, Grünwettersbach, bis runter dann an die Ettlinger Grenze, beziehungsweise nach Norden dann, wo der nördliche Schwarzwald eigentlich endet, also der Buntsandstein-Schwarzwald und in den Kraichgau übergeht. Und da ich jetzt schon ein gewisses Alter habe und schon Jahrzehnte die Situation überblicken kann. Also meine frühesten Kindheitserlebnisse reichen zurück in die Jahrzehnte meiner Kindheit. So alt bin ich schon. Und daher kenne ich also den Wald schon und seit der Zeit habe ich ihn immer wieder begangen, habe die Veränderungen wahrgenommen. Habe auch selber im Wald gearbeitet mit dem Vater also Holz gemacht, wie man sagt, Holz geschlagen, das Holz dann nach Hause gefahren und dann verarbeitet. Und, also jetzt verfolge ich natürlich, oder ich gehe nochmal ein bisschen zurück. 80er Jahre, also das berühmte Waldsterben damals, was einen völlig anderen Charakter hatte und Ursachen hatte als das Heutige, weil die Situation heute, die ist also nicht vergleichbar. Sie ist wesentlich dramatischer. Und das sind also die persönlichen Eindrücke und dann habe ich natürlich mit der Forstverwaltung ständig Kontakt als meine Arbeitsposition. Es gibt Begehungen, es gibt Gespräche, es gibt dann Sitzungen, wo man z.B. informiert wird über die Forsteinrichtung nächste, über Probleme, die es im Wald gibt oder Möglichkeiten, wie man Waldränder gestaltet. Also all das und das beschäftigt mich eigentlich oft während des Jahres. Und insofern denke ich, dass ich vielleicht ein paar Dinge dazu sagen kann.
4	**LT:** Sie haben das erste Stichwort schon genannt, und zwar Waldsterben in den 80er Jahren und daraufhin hatte die Forstverwaltung damals sich dem naturnahen Waldmanagement verschrieben. Und dieses Konzept wird immer noch verfolgt. Und auch im Konzept der Klimaanpassung angewendet. Wie würden Sie das bewerten?
5	**NA1:** Ja also für den Karlsruher Stadtwald muss man natürlich sagen, nach dem Krieg da waren z.B. durch Föderationszahlungen an Frankreich riesige Kahlschläge entstanden. Das musste relativ schnell aufgeforstet werden und man hat dann natürlich auch maschinell aufgeforstet. Also mein Vorgänger in der Arbeitsstelle, der war aus einem anderem Berufsfeld und war zuständig für den Hardtwald und der hat das sogenannte, so eine Art automatisches Pflanzverfahren entwickelt. Und das sah einfach so aus, dass man mit einer Maschine und da saßen ein paar Leute drauf durch den Wald gefahren ist. Da wurde eine Furche gezogen, die Pflanzen kamen rein und so hat man halt eigentlich wie in einer Gärtnerei relativ schnell große Flächen bepflanzt. Und dann natürlich mit einer Baumart, relativ uniform. Mit wenigen Strukturen. Und so ist also der Karlsruher Hardtwald entstanden und wir hatten eigentlich in den 80er Jahren weniger die Probleme im Karlsruher Stadtwald, das war dann eher die Höhenlagen, Schwarzwald z.B.. Weil diese Schwefeldämpfe, die von den nicht-entschwefelten, von den sauren Abgasen der Kraftwerke herrührten, die haben halt vor allen Dingen den Nadelbäumen sehr stark zugesetzt,

	den Fichten vor allen Dingen, Tannen. Die sind einfach über Karlsruhe hinweggezogen. Von daher war, sage ich mal, natürlich angesagt naturnaher Waldbau. Die sogenannten Plänterwälder, das sind also Wälder, die unterschiedliche Altersklassen haben im Gegensatz zum sogenannten Altersklassenwald, der irgendwann gepflanzt wird und da haben alle Bäume die gleiche Größe und werden irgendwann dann wieder gefällt. Und dieser Umtrieb, der hat dann einige Jahrzehnte, in einigen Jahrzehnten findet der statt. Und im Plänterwald, also in einem gestuften Wald, da werden Bäume dann je nach Hiebreife dann entnommen und es entstehen nicht mehr die großen Kahlhiebe, die heutzutage auch nicht mehr zulässig sind. Mal waren es 5 Hektar, heute gibt es kaum noch Kahlhiebe, die also kaum an einen Hektar ran reichen. Und insofern hatten wir mit dem ersten Waldsterben relativ wenig Probleme hier im Karlsruher Stadtwald. Also wie gesagt das war eher dann, das waren die Höhenlagen. Schwarzwald, da sah es also wirklich schlimm aus. Und Sie wissen, dann durch technische Möglichkeiten, durch Filteranlagen, konnte man dann diesen Problemen begegnen und das hat auch einigermaßen hingehauen und die Wälder haben sich etwas erholt. Man hat auch gekalkt. Es wurde stark gekalkt, um die Säure im Boden zu neutralisieren. Das hat man immer etwas kritisch gesehen. In Einzelfällen kann man das mal machen, aber letztlich ist es halt ein Eingriff in ein Ökosystem, was auf der einen Seite vielleicht aus forstlicher Sicht und aus ökonomischer Sicht notwendig ist. Aber auf der anderen Seite eben, den ökologischen Charakter eines Waldes deutlich macht, ist er gut oder ist er nicht so gut. Und natürlich haben diese uniformen Wälder diese Belastung nicht so gut vertragen. Was wir heute haben, das ist eine ganz andere Situation. Wir hatten drei Jahre extreme Trockenheit, sie wissen es. Und mittlerweile muss man sagen, diese Trockenheit, die hat dazu geführt, dass natürlich der Grundwasserspiegel extrem abgesunken ist. Und nicht nur die Fichten mittlerweile großflächig absterben, auch die Kiefern. Fichten hat man wenig, kaum eigentlich im Karlsruher Stadtwald. In Stupferich z.B. einige, aber es sind hauptsächlich die Kiefer. Und es sind mittlerweile auch die Buchen, die Rotbuchen, die leider ziemlich angegriffen sind, zum Teil. Und für die Rotbuche hat Deutschland eine besondere Verantwortung, weil die Rotbuche hier einen Verbreitungsschwerpunkt hat in Westeuropa. Und hinzu kommen dann noch die Pilzkrankheiten, Schädlinge und so weiter, die massiv auftreten, die natürlich die geschädigten, die schlechten Bäume dann umso mehr befallen. Aber es sind auch neuartige Krankheiten, wie z.B. das Eschentriebsterben, die Eschen fallen nahezu vollständig aus. Und dann haben wir z.B. die Rußrindenkrankheit bei Ahorn hauptsächlich. Und auch bei anderen Laubbäumen treten solche Pilzkrankheiten auf. Und das macht also ganz, ganz große Sorgen. Der Wald ist in einem schwierigen, ganz schwierigen Zustand. Das beschäftigt einen natürlich, weil man die Ökologie insgesamt sieht und weil man natürlich als Naturfreund im Wald unterwegs ist, man beobachtet z.B. über Jahre hinweg bestimmte Vogelarten und stellt fest, die sind entweder nicht mehr da oder es sind andere gekommen, die früher nicht da waren. Das sind Reaktionen der Natur, die vielleicht der Förster jetzt nicht in erster Linie sieht, natürlich denkt er auch an Nachhaltigkeit, aber dieser Begriff wurde von einem Förster erfunden vor über 200 Jahren, Nachhaltigkeit. Aber der war natürlich hauptsächlich aus forstlicher Sicht begründet, also ich muss so viel Mal nachpflanzen, dass auch die kommende Generation, Generationen jederzeit wieder was zu Ernten haben, diese Verpflichtung für die kommenden Generationen. Und das ist jetzt mit Sicherheit massiv unterbrochen, weil, was nachpflanzen? Wie schnell nachpflanzen? Lässt die Trockenheit, lässt der Klimawandel es zu, dass man relativ schnell einen oder sagen wir mal in absehbarer Zeit mit mindestens einem Großteil der einheimischen Bäume wieder einen entsprechenden Wald aufbauen kann? Das ist die große Frage.
6	**LT:** Jetzt haben Sie recht detailliert die Klimawandelfolgen aufgezählt oder genannt. Und das Forstamt reagiert darauf, eben durch dieses naturnahe Waldmanagement oder das Konzept zur Klimaanpassung, was auch veröffentlicht wurde letztes Jahr. Und da geht es auch ganz viel um die Baumartenauswahl und Naturverjüngung. Welche Lösungsansätze sehen Sie denn noch? Oder welche Konflikte sehen Sie bei diesen beiden Lösungsansätzen?
7	**NA1:** Also grundsätzlich ist es so, dass unser Wald, auch wenn es ein forstlich genutzter Wald ist, über die Jahrhunderte, über die Jahrtausende eigentlich seinen Charakter entwickelt hat. D.h., die

	Ökologie, d.h. das Zusammenspiel der Lebensformen vom kleinen Pilz oder von der Mikrobe bis zum Greifvogel meinetwegen, oder bis zum Reh und Hirsch, das hat sich einigermaßen in einer gewissen Abstimmung untereinander entwickelt. Das ist Ökologie, also das berühmte Netz, wenn nun Netze ausfallen, dann heißt das, je grobmaschiger dann dieses Netz wird, desto schwieriger wird es für die Lebensformen, dieses Netz wieder zu flicken und man denkt nun, dass man das Netz dadurch flicken kann, dass man jetzt Ersatzbäume nun pflanzt. Aber das Netz, was jetzt entsteht, das ist nicht mehr das von vorher. D.h., es wird also im gesamtökologischen Gefüge wird es Auswirkungen haben. Also das Auftreten von Schädlingen, das ist nur die eine Sache. Das Ausbleiben oder das Verschwinden anderer Arten, das ist die andere Seite. Man weiß, dass auch allein die Struktur des Waldes dazu führt, dass bestimmte ökologische Zusammenhänge sich verändern. Wir werden wahrscheinlich einen trockeneren Wald kriegen, d.h. also Feuchtstandorte werden weniger werden mit ihren typischen Lebenszusammenhängen. Und jetzt sagt der Forst natürlich, also wir gucken mal, was wir da pflanzen können. Jetzt hat der Forst natürlich ein Problem, er muss auch ökonomisch denken. D.h., die Waldbesitzer, ob das jetzt der Staat ist, ob das eine Kommune ist oder ein privater Waldbesitzer oder eine Stadt, also wie Karlsruhe, die haben natürlich auch ein Interesse daran, dass der Wald Gewinn abwirft, oder zumindest bis zu einem gewissen Teil auch Erträge bringt. Also der typische Waldbauer z.B., im Schwarzwald und so, der sagt, also der Wald ist meine Sparkasse. Und was den Staatswald oder den Stadtwald betrifft, da ist es nun so, dass der Karlsruher Gemeinderat schon vor einigen Jahren eigentlich darüber abgestimmt hat, wo sollen denn die Schwerpunkte liegen, der Nutzung des Waldes. Der Wald hat verschiedene sogenannte Wohlfahrtsfaktoren, also Erholung, Wasserschutz, Naturschutz und natürlich Ökonomie, das ist klar, Windschutz auch. Also es gibt verschiedene Wohlfahrtsfaktoren. Und da wurde klar bestimmt und klar entschieden, dass die Ökonomie, also der monetäre Aspekt, nicht an erster Stelle stehen sollte. Und wir haben schon den Eindruck, das wird ein Stück weit auch befolgt, nichtsdestotrotz denkt man natürlich drüber nach, also wie ersetzt man jetzt die entfallenden Nadelbäume, also z.B. durch die Douglasie, durch andere Baumarten und da haben wir natürlich unsere Bedenken, wenn man über eine bestimmte Fläche hinaus geht oder einen bestimmten Anteil hinausgeht. Also wir können es uns nicht vorstellen, dass wir in Zukunft in einem künstlich gepflanztem Mittelmeerwald leben. Die Natur ist nun so, dass sie sicher auch mit größeren Kalamitäten umgehen kann, also ob sie 100 Prozent zurechtkommt, das ist eine andere Frage, aber die Natur reagiert. Also der Wald stirbt nicht, der verschwindet nicht, es wird sich natürlich anderer Wald bilden. Und jetzt ist die große Frage, also was soll der Forst machen. Da wird geforscht, das ist richtig. In einer anderen Stadt in einer Institution werden natürlich auch Empfehlungen entwickelt und man versucht auch durch das Setzen von anscheinend gegen Trockenheit unempfindlicheren Arten den Wald wiederaufzubauen. Nur, das geht nicht von jetzt auf nachher, das ist ganz klar, also wenn man überlegt, eine Buche wird vielleicht mit 120 Jahren hiebsreif, das ist eine Sache der Generationen. Und wenn in der Zwischenzeit sich die Situation noch weiter verschärft, womit wahrscheinlich zu rechnen ist, leider also. Dann wird aus dem mit viel Energie und Liebe und Überzeugungskraft gesetzten Buchen oder Stieleichen, dann natürlich nur noch ein kümmerlicher Wald. Also es gibt sicher Arten, die relativ stabil sind, ich sag mal Feldahorn, Hainbuche oder andere Arten, da würde ich sagen ja, ist gut, kann man versuchen, die zu verstärken, das macht der Forst auch. Meine Arbeitsstelle wird auch immer eingebunden in die Projekte einer anderen Institution, das sind immer zehnjährige Pläne, die da entwickelt werden und die weitere Institution, die unterstützt das, die gibt diese Ratschläge. Aber man sieht manches kritisch, z.B. ist geplant, die Jungpflanzen zu bewässern im Wald, also das kommt dann nah an eine, an eine Waldwirtschaft ran, die eher an Gärtnern oder an bewässerte Landwirtschaft erinnert und das können wir uns eigentlich nicht vorstellen. Insbesondere auch deshalb, weil die Waldflächen möglichst auch ungestört bleiben wollen, das ist auch immer ein Eingriff in die Waldökologie. Also ich sage mal, es gibt z.B., von der Eiche gibt es auch südlichere Arten, die kann man auch mal probieren. Das macht der Forst auch, Flaumeiche z.B. solche Arten. Was mit großer Sorge zu betrachten ist, sind also invasive Arten, Neophyten, die sich mittlerweile breit gemacht haben und die fast nicht mehr

	in den Griff zu kriegen sind. Also ich sage mal die Spätblühende Traubenkirsche, Kermesbeere, das sind solche Arten, die ganze Bestände mittlerweile befallen und die eigentlich nur mit großem personellem Aufwand zu bearbeiten sind oder wiederherzustellen sind. Das ist ein Riesenproblem. Und aus Naturschutzsicht könnte man natürlich jetzt sagen, also wir lassen das jetzt alles grad so wie es ist und warten mal, was daraus jetzt wird. Nur, man hat natürlich auch riesige Mengen an Schadholz, mittlerweile ist ein Großteil des Holzes, was eingeschlagen wird, ist nicht das regulär einzuschlagende Holz, sondern das ist Schadholz, das muss auch bearbeitet werden, Borkenkäfer und so weiter, das wissen Sie. Und hinzukommt, dass die Walderträge, die Holzerträge im Moment dermaßen runtergehen, weil sehr viel Holz, containerweise geht es nach China, nach Nordamerika, in die USA, die kaufen sehr viel Holz auf, also da ist eine große Nachfrage nach Holz, das ist natürlich richtig, aber wie das mit der Nachhaltigkeit zu tun hat, wie man sie also als Naturschutzmensch oder als Biologe versteht, das ist meiner Arbeitsstelle noch nicht klar. Also man sieht einiges kritisch und ist mit dem Forst natürlich auch im Gespräch.
8	**LT:** Und welche politischen Instrumente wünschen Sie sich für die Zukunft, um den Wald an den Klimawandel anzupassen oder besser zu agieren?
9	**NA1:** Also ich denke, es braucht wirklich eine offene Diskussion der Betroffenen oder derer, die sich damit auseinandersetzen. Also das ist natürlich in erster Linie der Forst, das sind sicher auch die Waldbesitzer, es ist der Naturschutz, es sind die Ökologen, es gibt also ausgewiesene Waldökologen, die Naturschutzverbände habe ich erwähnt. Und es ist im Grunde auch die interessierte Öffentlichkeit, man muss das sehr transparent machen, man muss die Probleme darlegen, meine Arbeitsstelle den Eindruck, dass in der breiten Öffentlichkeit derzeit, das ist vielleicht auch Corona geschuldet, dass dieses zweite Waldsterben überhaupt nicht im Fokus ist oder nur am Rande. Und die Dramatik, die dahintersteht, weil es nicht um einzelne Baumarten geht, sondern weil es um eine Gesamtproblematik geht, um ein Gesamtsystem geht, die ist den Leuten oder großen Teilen der Öffentlichkeit und auch der Politik noch nicht so bewusst. Man hat von politischer Seite, also den Waldbesitzern, natürlich Geld auch gegeben, aber das deckt eben grade mal die Schäden oder einen Teil der Schäden, die entstehen durch Schadholz, durch nicht verkauftes Holz, Holz, das nicht mehr verwendet werden kann und so weiter. Aber ob jetzt im großen Stil die Waldökologie als Grundlage für eine zukünftige Waldbewirtschaftung im Mittelpunkt steht, da haben wir unsere Zweifel. Da müssen wir auf kommunaler Ebene, im Gemeinderat, mit den einzelnen Parteien, mit den Fachleuten, mit dem Forst natürlich ins Gespräch gehen und müssen unsere Vorstellungen, und das machen wir auch, entsprechend zu Gehör bringen. Momentan hat die Stadt Karlsruhe ein Konzept entwickelt und man hat kritisch dazu Stellung genommen, insbesondere was den Anteil fremdländischer Baumarten betrifft. Natürlich kann man Esskastanien pflanzen, das ist klar, aber über große Flächen kann ich mir eigentlich im Moment nicht vorstellen, dass der Wald ein völlig anderes Gesicht hat, zumal wie gesagt, die ökologischen Zusammenhänge dadurch sich völlig verändern und unter Umständen ganz ungewollte Entwicklungen auch auftreten. Wir wissen das einfach noch nicht.
10	**LT:** Danke, dann würde ich jetzt zum zweiten Themenblock schreiten. Und zwar ist es jetzt seit der Covid-19-Pandemie immer mehr vorgekommen, dass die Erholungsfunktion des Waldes auch in Karlsruhe, beim Karlsruher Stadtwald von der Stadtbevölkerung mehr gefragt ist. Sehen Sie da Konflikte?
11	**NA1:** Also zunächst mal ist es gut und schön, dass die Leute wieder mehr den Wald entdecken und die Wohlfahrtswirkungen des Waldes, also bis zum Waldbaden und ähnliche Dinge entdecken. Das ist gut und auch grade in der heutigen Zeit auch notwendig und man versucht, auch wenn die Situation sich wieder bessert, aber das hat man auch schon immer gemacht, durch Führungen die Leute z.B. zu informieren und auf besondere Dinge aufmerksam zu machen. Das ist die positive Seite. Was man feststellt ist, also dass Teile, zumindest Teile des Waldes in einer Art und Weise teils aus Unkenntnis, teils aus Böswilligkeit, teils aus einfach Nachlässigkeit überrannt werden. Also es gibt einige Beispiele, ich wohne in meinem Wohnort z.B. und da wurde in naher Vergangenheit ein Trail für Mountainbikes eingerichtet, weil da hat es schöne Abhänge und so weiter, das gab es schon immer eigentlich. Aber mittlerweile ist es so, dieser Trail, der ist

	jetzt so bekannt, bzw. wird bekannt gemacht auch über die sozialen Medien, da kommen die Leute aus zwei anderen Städten in dem Bundesland wo ich wohne angefahren und aus einer dritten Stadt in diesem Bundesland zum Teil mit Autos und was da noch ökologisch ist, das frage ich mich. Und die Spaziergänger, also der ganz normale Spaziergänger, Familien mit Kindern oder sonstige Erholungssuchende, die fühlen sich zunehmend genervt, zumal auch durch diese Mountainbikes auch entsprechende Waldschäden entstehen. Das ist ein Beispiel. Es werden Naturschutzgebiete betreten, das ist natürlich ganz schlimm, es wird Müll hinterlassen, und zwar wirklich an manchen Stellen in großen Mengen. Und das Niederwild und natürlich auch das Hochwild, also Reh und so weiter wird verscheucht, wird beunruhigt mit allen negativen Folgen. Die Jäger, die klagen, also seit einem Jahr mindestens massiv über diese Störungen. Hunde laufen frei durch den Wald, früher hat man sich das kaum getraut, weil Jäger auch mal einen wildernden Hund erschossen haben, das hat abgeschreckt, sie müssen eigentlich im Wald an der Leine bleiben. Aber wir haben zunehmend Fälle von Rehen, die von wildernden Hunden gerissen werden, man regt sich auf, wenn irgendwo ein Wolf mal ein Schaf tötet, das ist seine Nahrung, aber wir haben wirklich hunderte von Fällen gerissene Rehe oder anderes Wild durch wildernde Hunde, das ist ein Riesenproblem. Und der Bestand der Hunde hat auch enorm zugenommen, die Leute gehen nicht verantwortlich damit um, das ist eine weitere Geschichte. Lärm, Müll habe ich genannt. Auch illegale Corona-Feste, solche Dinge, hat es auch in meinem Wohnort schon gegeben. Also das sehen wir mit Sorge.
12	**LT:** Und bei den ganzen Problemen, welche Lösungsansätze auf politischer Ebene würden Sie da vorschlagen?
13	**NA1:** Gut, also man hat natürlich Kontakt zu der Forstverwaltung. Bestimmte Wege, die werden auch gesperrt, also wo es in empfindliche Bereiche geht, da hat man erreicht, dass der Forst diese Wege zulegt mit gemischtem Erfolg, manche klettern dann grade extra drüber. Aber es nützt etwas. Nur viele Leute haben da gar kein Verständnis dafür, weil, ich bin immer schon da gegangen und ich bin hier aus der Gegend, ich nehme mir das Recht raus und ich habe meinen Hund auch unter Kontrolle und so weiter. Das sind die Argumente. Man würde sich wünschen, dass die Stadt Karlsruhe, ich sage es jetzt mal konkret, über die Feldhut z.B., eine verstärkte Feldhut, stärker kontrolliert, die Leute auch drauf aufmerksam macht, und zwar dann auch nachdrücklich, unter Umständen auch Verstöße wirklich mal sanktioniert, was heutzutage praktisch nicht stattfindet. Es sind Gebote und Verbote und Freiheit ist auch immer Verantwortung. Und wenn man die Verantwortung für die Natur nicht wahrnimmt, dann ist es auch mit der Freiheit vorbei. Insofern versucht man auch auf die Politik und vor allen Dingen auch die Verwaltung, die Forstverwaltung oder auch die Feldhut z.B. Einfluss zu nehmen, dass da kontrolliert wird.
14	**LT:** Dann habe ich noch eine allgemeinere Frage, und zwar in welchen Kriterien würden Sie die Qualität des Stadtwaldes messen?
15	**NA1:** Also wir haben in weiten Teilen einen ökologisch-qualitativ guten Stadtwald. Vor allen Dingen da, wo die großen Eichenbestände sind oder Buchen auch noch oder wo der Mischwald ist und vor allen Dingen auch in den Rheinauen, da werden im Moment allerdings großflächig die alten Pappeln rausgenommen. Das sind zwar Hybrid-Pappeln aber die haben auch ihren ökologischen Wert und man sieht eigentlich nicht so richtig, wie diese Flächen entsprechend nachhaltig aufgeforstet werden sollen. Klar, Stieleiche ist eine Möglichkeit aber also der ökologische Wert. Man plädiert auch dafür, dass auch Pappeln stehen bleiben. Die einheimische Schwarzpappel, die gibt es fast nicht mehr, ist wirklich eine Seltenheit. Es sind halt Pappelarten, die sich bewährt haben, die ursprünglich nicht bei uns heimisch waren, aber die haben schon auch sozusagen Heimatrecht langsam. Was die Hangwälder und die Wälder auf den Hochebenen betrifft, also in den Bergdörfern wie Stupferich, wird die Fichte wahrscheinlich verschwinden, die wird man ersetzen müssen, das sind nicht so große Bestände. Und die sind auch über weite Strecken noch relativ, also ökologisch noch in Ordnung, weitgehend in Ordnung. Also man sieht das an den Vogelarten z.B. die man regelmäßig, wo regelmäßig auch Bestandsaufnahme gemacht wird und wo man auch Veränderungen dann wahrnimmt. Die Wälder sind dichter geworden,

	Stickstoffeintrag über die Luft z.B., meine Arbeitsstelle denkt, dass auch lichtere Wälder ihr Recht haben, also grade auf den Kiesrücken, der Hardtwald, der könnte aus Sicht meiner Arbeitsstelle auch durchaus mal lichtere Bereiche aufweisen, weil das fördert die Artenvielfalt besonders bei den Insekten ganz unglaublich. Und man muss das einfach tolerieren, dass einfach nicht jeder Quadratmeter, der natürlich rechtlich Wald ist entsprechend bestockt ist mit Bäumen, mit Forstpflanzen. Also da wird man dafür plädieren, etwas nicht lockerer zu sein, sondern einfach mehr Geduld zu haben, auch mal so Aufwuchs und auch natürlichen Aufwuchs zu dulden, weil sich da eben dann auch Arten einstellen, die auch irgendwo in den Wald gehören. Also ich sage mal Ginster, Heide, solche Flächen. Das hatten wir früher, ich kenne das noch nach diesen großen Hieben nach dem Krieg, kenne ich noch die großen Kahlflächen im Wald und da hatten wir Arten, die sind heute verschwunden. Also ein Zeichen dafür, der Wald war damals durchaus auch mit diesen Kahlflächen, die dann wirklich aufgeforstet wurden, der hatte auch seinen ökologischen Wert. Aber insgesamt würde ich sagen, Karlsruhe hat noch einen guten Waldbestand, den es zu schützen und zu bewahren gilt, aber mit Augenmaß und wie gesagt, unter den Prämissen, die ich geschildert habe. Also man muss im Gespräch bleiben, man muss das diskutieren, man muss den Forst auch mal zumuten, dass er vielleicht traditionelle Sichtweisen bei Seite räumt und einfach auch das Prinzip der Nachhaltigkeit, wie es die Ökologie sieht, in den Vordergrund stellt. Und das würde ich mir erhoffen von der Politik und von der Forstwirtschaft. Es wird viel geschehen, gebe ich zu und räume ich ein, aber man hat immer noch an vielen Stellen Gesprächsbedarf.
16	**LT:** Jetzt sind Klimawandel und die Covid-19-Pandemie sehr globale Themen, also auch wenn das den Wald betrifft, und finden Sie, dass sich sowas dann trotzdem am besten auf lokaler, regionaler Ebene lösen lässt oder angehen lässt oder sollte es dann eher auf höherer Ebene behandelt werden?
17	**NA1:** Also global denken, lokal handeln, das ist die Devise, denke ich. Wir müssen endlich nicht nur in Deutschland, natürlich weltweit, aber wir sind hier für Deutschland zuständig, ein Bundeslandwirtschaftsministerium ist auch ein Bundeswaldministerium, das muss wirklich unter Einsatz aller Fachlichkeit Konzepte entwickeln, vor allen Dingen auch für die großen Waldbesitzer, es gibt unheimlich große private Waldbesitzer auch, die davon leben, wie der Wald der Zukunft aussehen soll und wie man das bewerkstelligen soll, also diese Konzepte, die fehlen mir noch. Und das muss natürlich lokal runtergebrochen werden, anhand der besonderen Standortfaktoren, also es geht da um glaube ich viele lokale Situationen, die muss man berücksichtigen. Die Förster sind die Fachleute, die wissen das, die kennen ihren Wald sehr genau und sie müssen sich überlegen, inwieweit diese Konzeption, die wir erwarten und an der sicher auch gearbeitet wird, inwieweit die auf lokaler Ebene realisiert wird. Und da würde sich meine Arbeitsstelle schon gern mit einbringen. Wir haben da Fragen dazu, wir haben da auch teilweise eine ablehnende Haltung oder wir haben Anregungen und von daher denke ich, wird es eine spannende Zeit, weil mittlerweile auch dem Forst klar ist, sie können nicht mehr nur rumexperimentieren, sie denken, wenn man in Jahrzehnten denkt, muss es etwas sein, was auch wirklich nachhaltig ist.
18	**LT:** Jetzt gab es ja in Baden-Württemberg infolge des Holzkartellverfahrens eine Reformierung der Gewaltenteilung. Also dass der Staatswald von der Anstalt des öffentlichen Rest Forst BW jetzt übernommen wird und der Körperschaftswald immer noch vom Forstamt Karlsruhe. Was ist da Ihre Meinung dazu?
19	**NA1:** Also wir haben das nicht befürwortet, wir haben das, wir sehen das mit einem gewissen Ärger, dass da so verfahren wurde, weil die Forstverwaltung und vor allen Dingen die Fachlichkeit, die ist vor Ort eigentlich am besten platziert und die Forstverwaltung vorher, die hatte die gesamte Waldfläche in und um Karlsruhe im Blick und das ist jetzt ein Stück weit auseinandergerissen. D.h. also viele Entscheidungen, einmal die Forstnutzung und auf der anderen Seite der Waldbau, also die ökologischen Aspekte, das halten wir im Moment für auseinanderdividiert. Und das ist keine Entwicklung, die wir befürwortet haben, wir hätten es begrüßt, wenn die Fachlichkeit nach wie vor an einer Stelle verblieben wäre, so wie es vorher war. Wir können nicht verstehen, dass Zuständigkeiten dann zig Kilometer weiter platziert

	werden, was immer man sich dabei gedacht hat, das halten wir nicht für gut, weil es ist eben nicht vor Ort und ist eben, es sind Absprachen notwendig, die unter Umständen gar nicht greifen, also ich sag mal die Nutzung des Waldes, die ist sozusagen ausgegliedert durch diese Struktur. Und da fehlt uns der Zusammenhang grade mit dem Begriff der Nachhaltigkeit, das bedauern wir, dass das so gekommen ist. Wir haben es nicht befürwortet.
20	**LT:** Gibt es zum Abschluss noch etwas Wichtiges, was Ihnen im Gespräch noch eingefallen ist aber jetzt noch nicht angesprochen wurde?
21	**NA1:** Ja also, die Pandemie hat natürlich gezeigt, dass die Leute verstärkt, weil Alternativen fehlen, den Wald wieder entdecken, was stark diskutiert wird und da gibt es mittlerweile so viel Literatur drüber und das ist auch in der Literaturgeschichte so nachgewiesen, der Wald hat offensichtlich also grade in Deutschland auch eine gewisse tiefere Bedeutung oder fast eine mythische Bedeutung. Ich sehe das gar nicht jetzt ideologisch, sondern es ist einfach so, dass uns mit dem Begriff *Wald* mehr verbindet als die grünen Bäume. Das ist etwas, was ich sag es mal ein bisschen pathetisch, so in der Seele verortet ist. Und ich glaube, dass die Menschen, die brauchen so etwas auch, weil vieles geht verloren, viele Orientierungen, die sind nicht mehr da, das geht rasant. Werte verschwinden, andere Werte treten an deren Stelle und der Wald als eine Art Heimat, ich sage es in Anführungszeichen, ich gehe mit dem Begriff sehr vorsichtig um, und auch die innere Verfasstheit auch anspricht. Das halte ich für ganz wichtig und grade für die junge Generation, auch für die Kinder, die jetzt kommen, die sollen diese Erfahrung machen, weil die ist etwas ganz Besonderes. Und grade, weil ich anfangs den Buchenwald erwähnt habe, das ist eine Waldform, die ganz besonders anheimelnd ist, das ist eine Waldform, die sehr natürlich auch ans Gemüt geht. Und das halte ich für ganz wichtig, von daher denke ich, ist der Wald unser aller Anliegen unbedingt.

NA2

1	**NA2 (33:57.5 Minuten)**
2	**LT:** Sie haben die Datenschutzerklärung gelesen und sind damit einverstanden, dass das ich das hier aufnehme?
3	**NA2:** Ja, bin ich einverstanden mit.
4	**LT:** Haben Sie denn gerade in Ihrer Arbeitsstelle zurzeit was zum Thema Klima und der Stadtwald von Karlsruhe?
5	**NA2:** Ja also der Forst beschäftigt uns zurzeit sehr sogar. Sowohl der Karlsruher Forst, also man hat, wissen sie wahrscheinlich, man hat zwei Bereiche hier in Karlsruhe und um Karlsruhe, das eine ist der so genannte Stadtforst, also die Stadt ist Besitzerin eines Teils des Hardtwaldes und Auwaldes und so weiter und das andere der Landkreisforst oder Staatsforst ist das eigentlich vom Landkreis dann bearbeitet und uns beschäftigt das was vor allem der Stadtforst macht im Moment sehr. Ich denke, das kann man sagen, das ist kein Geheimnis: zum einen wurde ein Antrag gestellt auf Bewässerung aus öffentlichen Gewässern für die Bäume, die man pflanzt und da haben wir natürlich eine Haltung dazu in unserer Position, die dem Forst so nicht gefällt, wir werden auch ins Gespräch eintreten. Und dann beschäftigt uns die Forstarbeit, also die Forstwirtschaft, die unserer Meinung nach einfach nicht mehr in die Zeit passt. Und da geschehen Dinge, die uns entsetzten. Nicht nur uns, ganz nebenbei, sondern man bekommt oft, kommt über Kontakte von Menschen, die durch den Wald laufen z.B., entsetzte Rückmeldungen, was macht der Forst hier? Wo dann Kahlschläge stattfinden. Wobei die Kahlschläge das Eine sind und wie des Anderen, wie diese Kahlschläge produziert werden. Also mit welcher Brachialität. Da werden schwerste Maschinen eingesetzt, also gerade in der Nähe von einer Ortschaft, kenne Sie die Ecke auch? Das ist bei Karlsruhe. Wo es gerade um einen See herum wirklich unglaublich intensiv eingegriffen wird, sogar mit Bodenveränderungen und so weiter. Und das sind natürlich Dinge, die einen umtreiben. Und ganz schwer, man hat jetzt schon gemerkt, dass es ganz schwierig ist eine übergeordnete Stelle zu finden, die sich dann mit dem Forst in Verbindung setzt, weil ganz

offensichtlich vom Gesetzt her der Forst autonom und privilegiert handeln kann, solange er, wie man so schön sagt, das so genannte ordnungsgemäße Forstarbeit rechtfertigen kann. Aber einen beschäftigt schon sehr, was hier geschieht und vor allem eben die Kahlschlagpolitik der Forstes, die Forstwirtschaft und sie beschäftigt einen vor allem auch deshalb, weil man in Karlsruhe im Stadtwald eigentlich eine gute Voraussetzung hätte für eine nachhaltige Forstwirtschaft, und zwar deswegen weil der Gemeinderat beschlossen hat, dass der Karlsruher Forst sich ganz eigentlich auf eine nachhaltige Forstarbeit konzentrieren kann und nicht gezielt aufgefordert wird irgendwelche Wirtschaftlichkeit zu verfolgen und da sieht man eine Diskrepanz, wobei man inzwischen der Meinung ist, dass es wohl daran liegt, dass die verschiedenen Zeiten die Begriffe, die hier im Spiel sind "Nachhaltig", "Zukunftsorientierung" und so weiter unterschiedlich geführt werden. Sie kennen wahrscheinlich aus ihrem Studium, davon gehe ich jetzt mal aus, die Tatsache, dass der Begriff der Nachhaltigkeit ursprünglich ein Begriff ist, der vom Forst geprägt wurde im 19. Jahrhundert und der meinte nichts anderes als, dass das was weggenommen wird aus dem Wald wieder nach gepflanzt wird. Sodass der Wald in etwa in einem Gleichgewicht bleiben, weil es natürlich durch einen Einschlag erstmal zerstört werden, aber durch die Dauer danach folgt, die Zeit danach folgt wieder aufgeholt werden und Nachhaltigkeit im Sinne von Naturschutz und Klimaschutz ist was anderes. Da geht es viel mehr als ein Wald, ein einigermaßen grob gehaltenes Waldgleichgewicht zu erhalten, sondern da geht es natürlich darum welche Funktion soll der Wald erfüllen, welche, oder wie kann man den Wald so aufstellen, dass er den neuen Klima Anforderungen besser gerecht wird? Da sieht der Forst auch ganz andere Ansätze. In Karlsruhe z.B. wird befürwortet, dass mal eben mit südlichen Bäumen, mit Bäumen aus einer anderen Region versuche macht und meine Institution sieht es eigentlich anders. Der versucht zu erreichen -ganz kurz nochmal Zwischenbemerkung: Sie wissen wahrscheinlich, dass in Karlsruhe im Moment eine Arbeitsgruppe darin arbeitet ein bestimmtes Konzept zu erstellen für die ganze Stadt, unter Federführung von einer Politikerin der Umwelt sozusagen. Eine politische Institution ist beteiligt, viele, viele Interessengruppen. Sie kennen die Damen, die vor allem in diesen Ämtern arbeiten, vor allem auch in einer anderen politischen Institution- und da ist tatsächlich einer der entscheidenden Punkte in der Diskussion mit dem Forst gewesen, welche Bäume befürwortet man? Und der Ansatz meiner Arbeitsstelle war eigentlich der, dass man versucht die heimischen Bäume zu fördern, weil man mit der spätblühenden Traubenkirsche sehr, sehr schlechte Erfahrung gemacht hat. Man hat einfach einen Baum von woanders hergeholt, der dort wunderbar funktioniert hat, also wunderbar im Sinne des Forstes. Schnell hochwachsend, kerzengerade hochwachsend und nach einer überschaubaren Zeit wunderbar zu ernten. wie die Förster sagen, und damit Holz zu gewinnen. Und der hat sich hier völlig anders entwickelt und hat hier eben in dieser anderen Umgebung sich zu einem invasiven Neophyten entwickelt, der die Waldstruktur verändert und inzwischen hat einer der großen der bekannten Botaniker hier -vielleicht kennen Sie den Namen auch- mal gesagt bei einen Treffen mit dem Forst, finden wir die spätblühende Traubenkirsche bis hoch in den Schwarzwald schon. Also nicht nur hier unten in dem Gebiet nahe meines Wohnortes, in unseren Wäldern -ich wohne in meinem Wohnort und bin sehr regelmäßig in den Wäldern unterwegs-, sondern eben das zieht sich hoch bis in Regionen des Schwarzwaldes und es ist eine Bedrohung für unsere einheimischen Gehölze. Und wir wünschen uns, dass diese -Forst gibt inzwischen zu, gesteht zu, dass das ein Fehler war- der ist aber nicht mehr reversibel, weil der Baum ist da, er wird nicht mehr verdrängt werden können, weil er auch eine große Kraft sich zu auszuweiten zu expandieren hat und wir haben dafür plädiert, dass man solche Experimente unterlässt solange man nicht Erfahrungen abschließen Bäumen in unserem Klima einerseits und in unserer doch besonderen Bodenstruktur, Klimastruktur, Biotopstruktur. Im Auwald, am Rhein, um den Rhein herum, ist noch mal ein ganz anderes Bioklima als, von mir aus, in Italien oder in Südfrankreich oder sonst wo. Also da haben wir eine höhere Luftfeuchtigkeit, Bodenbeschaffenheit und man kann eigentlich nur seriös neue Bäume aus anderen Umgebungen hier anpflanzen, wenn man Erfahrung gesammelt hat, um zu verhindern möglicherweise, dass die wieder ähnliche expansive Verhaltensweisen entwickeln, wie es eben die spätblühende Traubenkirsche gemacht hat. Und das ist im Moment ein wichtiger

	Auseinandersetzungspunkt, der noch lange nicht entschieden ist. Und der Forst befürwortet, das weiß man, bestimmte Bäume die schnell wachsen natürlich, die schnell Holz ergeben für die Weiterverwertung, wirtschaftlich soll der Forst sein und so weiter. Also insofern, das ist eine so ganz, ganz dringende Frage, die wo im Moment noch, also in naher Zukunft ist wieder ein Treffen für ein Projekt, wo es um Biodiversität, zum Klimakonzept geht der Stadt Karlsruhe und es weiter diskutiert wird. Und da der Forst eben diese, ich sage jetzt mal, diese Unabhängigkeit hat, die er hat, weiß man nicht wie er sich aufstellen wird. Also was man erreicht hat schon ist er hat eine Pyramide gehabt, der Baumarten, die er bevorzugt und die wurde durch Gespräche und intensive Auseinandersetzungen, die wurde schon korrigiert, kann man sagen. Und das ist zunächst mal erfreulich, aber wie jetzt dann in sagen wir mal vier, fünf Jahren, in drei, vier Jahren die Anpflanzungspolitik aussehen wird des Forstes können wir noch nicht einschätzen. Zumal, vor Kurzem habe ich erfahren von einer Freundin und Kollegin, auch schon seit einiger Zeit, die in einer anderen Region in meinem Bundesland, also eher da woanders, in ihrem Arbeitsort da arbeitet und im gleichen Metier, also den gleichen Beruf, wie ich, dort hat, die hat gesagt, da gibt es einen Förster, der will 30% Douglasien pflanzen. Und das ist natürlich jetzt nicht das was wir unter einem nachhaltigen Wald verstehen. Wir sehen ihn eher als einen Wald, der einerseits einen Mischwald natürlich darstellt, aber vor allem einen Laubmischwald und Mischwald heißt, dass Arten gemischt werden und man hat den Hardtwald und dann noch eben die Auwälder. Und gerade in den Wäldern meines Wohnortes bin ich vor Kurzem mit jemanden von einer anderen Institution durch, meine ich, einen Wald bei meinem Wohnort gelaufen, das ist um einen See herum und der hat auch gesagt, also die Arten, die Baumartenvielfalt ist viel zu gering. Weil die Artenvielfalt, und zwar eine heimische Bäume, er meinte in die dortigen Wälder passen gut, vor allem in einem Bereich, passen gut bis zu 20 Laubholzarten, die man aber eigentlich nicht mehr antrifft dort, weil der Forst auch, das ist eine andere Entscheidung, die er im Moment trifft und uns in meiner Arbeitsstelle damit ein bisschen ködern möchte. Er plant vor allem, das wissen sie wahrscheinlich, Eichen. Und sagt nun, das ist doch eine ganz wertvolle Geschichte, weil es ökologisch ein hochwertiger Baum ist. Stimmt. Aber erstens mal nach dem Kahlschlag etwa 60, 70 Jahre kaum. Also dieser Zeitfaktor, das ist natürlich eine Sache, da muss man sich dran gewöhnen. Also zuerst mal sagen, wir sind nachhaltig und ökologisch, weil wir pflanzen Eichen. So einfach ist die Sache nicht unserer Meinung nach, sondern erstmal wird auch ein Vakuum geschaffen. Wo ein funktionierendes Biotop durch Kahlschlag erstmal auch beeinträchtigen wird. Ganz nebenbei auch natürlich durch die Öffnung des Waldes eine Angriffsfläche für die Wärme, für die Hitze, die Einstrahlung und zunächst mal auch der Schutz, den der Wald darstellt, also man sagt normalerweise gehen drei bis fünf Grad Wärme- oder Kälteunterschied in einem funktionierenden Laubmischwald. Das hat man da eben an diesen Stellen so nicht und je größer die Kahlschläge sind, desto problematischer wird es dann zusätzlich und wenn ich dann hinterher hergehe als Forst und möchte aus den Gewässern auch in Naturschutzgebieten Wasser entnehmen und diese Gewässer noch mehr, sozusagen, in Bedrängnis bringen, die auch Probleme haben, mit der Erwärmung, dann wird es problematisch. Jetzt habe ich lange geredet, haben sie fragen?
6	**LT:** Die Baumdiversität. die man -jetzt z.B. was Sie angesprochen haben- in den Auenwäldern abnimmt: ist das Klimawandel bedingt oder vom Waldmanagement her?
7	**NA2:** Die ist nicht klimabedingt. Im Moment weiß man, das ist Ihnen vom Studium her sicher sehr bekannt, dass der natürlich im Grunde fast alle Bäume Probleme bekommen. Durch die Erwärmung. Zum einen sind es zunächst mal Bäume, wo man denkt, komisch. In meinem Wohnort, die Kiefer z.B., die gerne sandige, also trockene Böden mag, aber sie mag die Hitze nicht, also der Kiefer geht es hundsmiserabel und es müssen viele Kiefern herausgenommen werden. Aber im Grunde kann man sagen, fast kein Baum leidet nicht in einer bestimmten Weise unter dieser Veränderung und den steigenden Temperaturen. War lange auch gedacht, die Rotbuche ist so ein wunderbarer Baum, der diesen klimatischen Veränderungen standhält, man weiß inzwischen auch der Rotbuche geht es schlecht, aber der Grundgedanke des hat mir dieser Kollege -ist kein Kollege von mir, sondern das ist der Mitarbeiter einer anderen Institution- gesagt, je

	größer die Vielfalt der Bäume ist, die dann auch egal in welchem -also vor allem Laubbäume- egal in welchem Wald stehen, umso größer ist die Chance, dass nicht wieder irgendein Parasit kommt, ein Pilz, eine Krankheit, die dann wieder eine Art wie z.B. die Esche, kennen Sie das Eschentriebsterben, beeinträchtigt und der so zusetzt, das dann innerhalb von zehn Jahren der Wald, eigentlich der Wald völlig verloren gegangen ist, weil dieser Baum nicht mehr bestand hat. Und ganz nebenbei, wenn man bisschen über eine Gebietsgrenze rübergehen, ein Nachbarbundesland oder die Nachbarregion liegt uns gegenüber: ich wundere mich immer wieder beim Spaziergang, Wanderungen, der Wald ist dort anders aufgestellt, weil dort tatsächlich der Laubmischwald viel stärker eine Rolle spielt als hier. Man konzentriert sich nicht auf ein paar wenige Arten, sondern schaut, dass eine Vielfalt dasteht und das tut dem Wald offensichtlich gut. Und das wäre für uns ein Konzept, um, also Laubmischwald gilt sowieso eben als eine sinnvolle Alternative oder ein stabilisierender Faktor für diese immer, immer stärker Erwärmung in den Sommern und gleichzeitig die Vielfalt als eine Chance um künftige Herausforderungen wie Krankheiten, Parasiten und so weiter auch besser aufzufangen. Insofern sind diese Dinge hier der Hintergrund dafür, dass wir das befürworten und was nicht heißt, dass die Einzelbaumarten nicht auch durchaus leiden. Ich meine, wenn es die Bedingungen hat um sich so, ich sage es jetzt mal ein bisschen pathetisch, in den prächtigen, prächtig zu entfalten, wie es eben vor 15, 20 Jahren noch der Fall war.
8	**LT:** Und neben der Baumartenauswahl: Welche anderen politischen Instrumente sehen Sie noch, um den Karlsruher Stadtwald gegen den Klimawandel zu schützen? Oder eben vorzubereiten, anzupassen, könnte man auch sagen.
9	**NA2:** Schwer zu sagen, weil, wie gesagt, ich habe Ihnen die zwei Bedingungen genannt, die in Karlsruhe prägend sind. Das Eine ist die Entscheidung des Gemeinderates, eine gute Entscheidung, dem Forst gegenüber keine Forderungen hinsichtlich der Wirtschaftlichkeit zu stellen. Finde ich eine ausgezeichnete, sie wissen da, dass die Grünen in Karlsruhe eine große Fraktion haben, das ist die bedeutendste Fraktion, die haben bei der letzten Wahl, Gemeinderatswahl, meine ich, 30% oder mehr bekommen, also stellen eine große, große Anzahl und damit mächtige Anzahl von Abgeordneten dar. Da wurde ein mehrheitlicher Beschluss gefasst, der das so sieht und auf der anderen Seite haben wir den Forst, der aber eben eine Vorstellung von Forstwirtschaft und von Nachhaltigkeit hat, die nicht mit dem im Einklang steht. Insofern und das ist ein Ansatz im Moment ist man gerade dabei mit dem Forst zu reden einerseits, was schwierig ist, aber es ist noch möglich, in naher Zukunft hat man auch ein Treffen, also ein Zoom-Treffen auch mit dem hiesigen Forstamtsleiter und wahrscheinlich werden auch seine Mitarbeiterinnen und Mitarbeiter dabei sein, die man gut kennt. Und daneben aber auch über die politische Schiene bestimmte Projekte des Forstes zu hinterfragen und zu bitten, dass die Politik prüft ob das im Sinne dieses Entschlusses ist, dass das vor ein paar Jahren gefällt worden ist. Weil man will keine Aufstände anzetteln und auch nicht. Könnte man. Weil unsere Zeiten, das wissen Sie auch, da wird schon schnell, weil man hört immer wieder von recht aggressiven Aktionen in den Wäldern, die stammen hundertprozentig nicht von meiner Institution. Wo Maschinen angegriffen werden, also ich habe das mit Entsetzten bei meiner Begehung ein bisschen weiter weg von hier, mit einem Kollegen und mit einem Förster länger unterhalten und der hat gesagt ein Landwirt, der sich auch auf Waldpflege konzentriere, dem wurden teuerste Maschinen zerstört. Und das geht gar nicht! Das ist kein Ansatz, also wir sind keine Maschinenstürmer und schon gar keine Sachbeschädiger, sondern wir möchte in einem sachlichen, fachlich-sachlichen Austausch versuchend eine Linie zu finden, wo wir uns besser ein bisschen annähern gegenseitig. Das wäre schon eine gute Sache. Aber die Prinzipien habe ich Ihnen im Grunde genannt, die uns wichtig und richtig erscheinen und insofern.
10	**LT:** Dann würde ich jetzt ein bisschen das Thema wechseln. Und zwar erfüllt der Wald auch die Erholungsfunktion und man hat gemerkt oder es gibt auch einige Studien, dass jetzt vor allen Dingen auch seit dem letzten Jahr, als die Covid-19-Pandemie begonnen hat, immer mehr Mensch den Wald nutzen, als Ausgleich vom Alltag und da wollte ich fragen, ob Sie bei der jetzigen Stadtwaldverwaltung Konflikte im Hinblick der Erholungsfunktion oder der ansteigenden

	Nachfrage der Erholung sehen?
11	**NA2:** Also sehe ich eigentlich nicht. Ich meine, der Forst weiß, das betont er auch immer wieder bei Begegnungen und Stellungnahmen, dass der Wald mehrere Funktionen erfüllt und erfüllen soll. Eine ist die Forstwirtschaft, die andere ist die sozusagen die Entwicklung der Biodiversität oder Erhaltung der Biodiversität und das andere ist natürlich, dass das ein wichtiger Erholungsraum für die Bevölkerung ist. Dass die Bevölkerung jetzt über Corona vor allem das spielt jetzt eine Rolle, also diese besondere Situation, die wir im Moment haben, muss man als solche auch betonen. Wie sich das hinterher weiterentwickelt weiß niemand, es könnte sein, dass es die Menschen insgesamt zu einer intensiveren Begegnung zur Natur herangeführt hat diese Zeit und es auch hinterher so bleibt. Weiß noch keiner. Also ich bezweifle, dass wenn die Menschen wieder weiter wegdürfen, wird sich das verlaufenen ein bisschen. Der Forst empfindet, die Forstverwaltung empfindet jetzt diese Dinge zunächst nicht als eine Bedrohung oder Belastung oder sonst irgendwas. Zumal kennen sie die Wälder bei Karlsruhe? Man hat viel Wald in Karlsruhe, unglaublich viel Wald eigentlich. Der Hardtwald ist ein sehr großer, der sich da im Grunde von Rastatt her bis nach Mannheim fast hinziehen und so weiter und die Auwälder sind auch sehr großzügig eigentlich und großflächig noch aufgestellt. Also man hat Raum für diesen Menschen. Was allerdings ein Problem darstellt, das ist die Art und Weise wie die Menschen diesen Wald besuchen. Und da stellt man fest: es gibt zwei Dinge, die man da beachten muss hierbei. Man hat in Karlsruhe im Grunde in unserer Umgebung eigentlich im Wesentlichen Natura2000, FFH-Gebiete und -die Begriffe kennen Sie, in ihrer Bedeutung- und dann eben zwei, drei Naturschutzgebiete. Und man stellt fest, dass die Menschen, warum auch immer, leider auf die Regeln, die im vor allem Naturschutzgebieten herrschen und vorgegeben sind -Sie kennen das Wegegebot, Hunde anleinen, keine Feuer und so weiter und sofort- dass diese Dinge leider nicht immer berücksichtigt werden. Und jetzt sage ich mal, das ist eigentlich meine Arbeitsstelle. Der Forst kommt damit weniger in Konflikt, weil er in seiner Arbeit eigentlich nicht beeinträchtigt wird dadurch. Aber wir in meinem Arbeitsmetier sagen natürlich, wenn die Wege nicht nur eingehalten werden und plötzlich quer durch wichtige Wiesen, durch wichtige Waldstücke Trampelpfade entstehen, ganz willkürlich. Vor allem was man festgestellt hat in letzter Zeit das unglaublich Müll zurückgelassen wird. Dann wird anscheinend gefeiert -ist in Ordnung, wenn man zu viert, zu fünft mal mit Freunden in der Nähe von dem Gewässer feiert- dann wird der Abfall aber leider nicht mitgenommen. Oder wenn ein Lagerfeuer gemacht wird, dann geht es schon viel zu weit. Zumal ein Kollege hat mir heute morgen erzählt, bei ihm im Arbeitsort war kürzlich ein Waldbrand ein kleiner, warum auch immer der entstanden ist. Und das sind Gefährdungen, die eigentlich wahrscheinlich uns mehr belasten oder beschäftigen, als den Forst. Weil, wieso sollte der Forst mit mehr Menschen, die mit dem Fahrrad durch den Wald fahren oder wandern, spazieren durch den Wald Probleme haben? Sehe ich eigentlich nicht.
12	**LT:** Sehen Sie eigentlich, dass so globale Probleme, wie z.B. der Klimawandel lieber so klein wie möglich, also auf lokalster Ebene geregelt werden sollte oder sollte sich da mehr national oder international mit befasst werden?
13	**NA2:** Natürlich beides. Also man kann ein Phänomen, wie den Klimawandel nicht lokal lösen. Also einerseits ist eine erstmal eine nationale Aufstellung, wie haben bestimmt in Ministerien, die sich darum kümmern oder auch nicht. Sie wissen es gab vor kurzem, vor wenigen Tagen ein bahnbrechendes Urteil: des Bundesverfassungsgerichts, ich habe Freudentänze aufgeführt, weil möglicherweise dieses Gesetz, erstmal hat dieses Gesetz der Jugend in ihrem Engagement für den für oder gegen den Klimawandel und für eine andere Klimapolitik unglaublich Recht gegeben, weil es geht um die Jugend, was die alten Herren in den Regierungen und Parteien oft vergessen, deswegen finde ich das großartig, dieses Urteil. Und wichtig ist jetzt, dass man tatsächlich auf nationaler Ebene zunächst mal z.B., es gibt eine Politik, die sich mit der Aufforstung beschäftigt oder mit der Unterstützung der Waldbesitzer, wie kann man die Waldbesitzer in diesen schwierigen Zeiten unterstützen. Dann gibt es ein Ministerium, das hat auch mit Wald zu tun letztlich, nämlich Landwirtschaftsministerium, der sich um solche Fragen eigentlich kümmern müsste und diese Frage, ich will das jetzt nicht bewerten. Das ist hier keine politische

	Veranstaltung. Wie die Bundesministerin sich da aufstellt, muss man natürlich untersuchen. Und dann, ganz bedeutend, vor Kurzem war der große Weltklimagipfel: das ist die Ebene, wo man das nur lösen kann letztlich. Und deswegen bin ich, ganz unter uns, sehr dankbar, dass der amerikanische Präsident inzwischen ein anderer ist. Der sehr gute Signale ausgesandt hat. Wie ernst auch die USA den Klimawandel nehmen und was er tun gedenkt, um das anzugehen. Also das ist die politische Ebene. National, international und nun sind wir beim andern, aber eben auch auf der kleinsten Ebene, dort wo die Menschen leben und nun sich selbst sich einsetzen können für etwas was bedeutsam ist und dazu zählt tatsächlich auch, deswegen ist man in Auseinandersetzung mit dem Forst, dazu zählt, was macht der Forst, wie sieht man das, trägt das, was der Forst macht dazu bei das Klima, den Klimawandel abzufangen, auf zu puffern oder eher nicht und was macht die hiesige Politik vor Ort? Also wie gestaltet sie unsere Stadt z.B.? Und da geschieht einiges, deswegen auch dieses Konzept das gerade entwickelt werden soll. Und wichtige Fragestellungen oder Weichen für die Zukunft wie man vor Ort in einer Stadt arbeitet und sich aufstellt, um eben den Klimawandel, die Erwärmung, es geht vor allem auch in der Stadt um Hitze und Erwärmung einer Stadt, wie man das auffangen kann und abfedern kann. Also alle Ebenen sind bedeutsam.
14	**LT:** Und wie ist dann Ihre Meinung zu dem aus dem Holzkartellverfahren resultierenden, oder diese Reformierung der Gewaltenverteilung, also dass der Staatswald von der Anstalt des öffentlichen Rechts ForstBW übernommen wurde und in Karlsruhe jetzt der Stadtwald oder Körperschaftswald nur noch vom Forstamt übernommen wird?
15	**NA2:** Also wir haben die, Sie meinen die Forstreform? Meinen Sie die Forstreform, die jetzt vom Minister in den letzten Jahren zum Ziel geführt wurde? Meinen Sie die? Ja, wir haben es kritisch gesehen. Wir haben das sehr kritisch gesehen, weil und vor allem deswegen, weil die Struktur unserer Meinung nach nicht geeignet ist, eine ortsnahe. Und da sind wir genau bei dem Punkt. Eben habe ich gesagt und betont, wie wichtig es ist vor Ort zu handeln. Ganz konkret und so wie der Staatsforst zumindest -der Stadtforst ist was anderes, der sitzt [29:06.2-29:08.8 nicht transkribiert, wegen Anonymität] Mitten im Hardtwald. Der ist genau richtig platziert und von da aus kann er die Auwälder bearbeiten, kann er den Hardtwald bearbeiten und so weiter- der Staatsforst hat sein Forstamt, soviel ich weiß, für die Staatswälder hier um uns herum in Eppingen. Wissen Sie wo das liegt? Fast im schwäbischen? Also ich hoffe, dass ich nichts Falsches sage, wenn ich was Falsches sage, bitte ich dies zu korrigieren. Ich möchte jetzt nicht mit diesem Begriff da Stimmung machen, aber was ich sagen will, für uns ist die Struktur die vorliegt für den Staatsforst nicht zielführend, weil er Menschen in Verantwortung bringt für uns hier z.B., die ganz selten hier vor Ort sind, riesige Wege haben hier anzureisen -natürlich sind die Förster hier, das ist klar- aber die Leiter der Ämter, die sind ganz weit weg und man weiß, ich kenne das, ich bin Romanist z.B. ich kenne es aus Frankreich: eine Zentralregierung, eine zentralisierte Führung eines bestimmten Themas ist anders als die regionale. Das haben auch die Franzosen, deswegen irgendwann in den 80er Jahren verstanden und haben ihren Zentralismus in Regionalismus teilweise zumindest umgewandelt. Und hier geht man den anderen Weg und das bedauern wir sehr. Weil je weiter ich von irgendwo weg bin auch ansässig bin, umso weniger fühle ich mich verantwortlich und verbunden. Ganz nebenbei, deswegen bedauern wir auch -das wäre übrigens ein Thema vielleicht auch für ihre Arbeit- deswegen bedauern wir sehr wie hier Forstarbeiten vergeben werden. Das sind oft -weiß nicht, ob sie es kennen schon- das sind oft Fremdfirmen. Die kriegen vom Förster, vom Forstamt den Auftrag das und das zu machen. Und dann kommen diese Leute aus Österreich, sie kommen aus Polen, sie kommen von mir aus noch relativ nah aus der Pfalz drüben, aber das sind alles sozusagen Unternehmen, die nicht hier angesiedelt sind und die genau wissen, sie sind hier eine Woche dann sind sie wieder weg. Und genauso arbeiten sie, unsere Meinung nach. Nämlich nicht mit der gebotenen Sorgfalt, nicht mit der Behutsamkeit, weil sie wissen, bis sich die Leute aufregen bin ich aus der Schusslinie. Und das bedauern wir sehr. Man könnte sowas auch anders machen, aber da geht es natürlich auch wieder um Kosten: polnische Arbeiter sind eben günstiger einzustellen, bei österreichischen wunder ich mich, aber bei polnischen kann man das sicher sagen, aber das sind Dinge in der Struktur und der

	Organisation des Forstes, die wir fragwürdigen finden, weil sie nicht Verantwortung fördert und nicht -ich denke, das ist vielleicht ein wichtiges Wort- Identifikation. Ich sage jetzt mal mit einem Menschen, der hier wohnt und lebt, der identifiziert sich anders mit der Umgebung, als jemand der von 2000 km oder 1000 km hier anreist zum Arbeiten. Und dann entsteht auch eine andere Art zu arbeiten. Wir würden uns wünschen, dass das anders organisiert wird und dadurch vielleicht auch eine größere Behutsamkeit entsteht. Also das fände ich einen wichtigen Aspekt: die Pflege der Wälder für, ich weiß nicht, ob sie das vorgedacht haben schon und eh berücksichtigen bei Ihrer Arbeit, aber das ist ein. Also Pflegemaßnahmen in den Wäldern und Kahlschläge: wer führt die eigentlich durch? Das halte ich für ein interessantes Thema. Ja, es ist ein hochinteressantes Thema, weil wir immer wieder sagen, guck dir an wie die die Bäume rausziehen. Die ziehen die lange erstmal machen sie Rückegassen -der Begriff ist Ihnen bekannt- die machen sehr breite Rückegassen, fahren mit schwersten Maschinen rein, hinterlassen unglaubliche Spuren und dann ziehen sie die Bäume noch so raus, dass sie zwei, drei, vier andere voll intakte Bäume beschädigen. Das ist nicht gut. Also wir dokumentieren das immer mit Fotos auch, deswegen kann ich das so sagen, ich habe genug Fotos und gerade was jetzt in einer Ortschaft passiert ist, wenn sie das sehen, da wird es jedem Menschen, dem der Wald am Herzen liegt übel, richtig übel. Okay. Aber wäre ein Thema, wäre ein Thema. Pflege.

NA3

1	**NA3 (12:40.4 Minuten)**
2	**LT:** Was haben Sie denn für einen Bezug zum Karlsruher Stadtwald?
3	**NA3:** Persönlich bin ich nicht allzu oft dort, aber durchaus auch schon mal dort gewesen auch in meiner Funktion bei Projekten zusammen mit Forstverwaltung, sowohl Stadt Karlsruhe als auch Landkreis Karlsruhe.
4	**LT:** Und was bedeutet für Sie Naturschutz? So ganz allgemein.
5	**NA3:** Naturschutz bedeutet, den Erhalt unserer natürlichen Umwelt. Was Biotopflächen, Tiere und Pflanzen bedeutet. Und möglichst den Zustand dieser Flächen nicht nur zu erhalten, sondern auch zu verbessern.
6	**LT:** Und kennen Sie das Konzept zur Klimaanpassung für den Stadtwald von Karlsruhe?
7	**NA3:** Ja so ungefähr. Also ich habe es jetzt nicht parat, aber ich bin darüber schon informiert worden.
8	**LT:** Und wie würden Sie die Klimaschutzanpassung von Karlsruhe bewerten?
9	**NA3:** Etwas zwiespältig. Für sinnvoll, sehr sinnvoll halte ich es, wenn man noch stärker -und das ist wohl vorgesehen- auf Naturverjüngung setzten möchte. Denn ich denke, was natürlich unter den jetzigen Klimaumständen auch wächst wird das Klima eine ganze Weile noch vertragen. Für wenig gut halte ich es, wenn man jetzt mit fremdländischen Baumarten verstärkt arbeiten möchte.
10	**LT:** Sehen Sie noch weitere Konflikte in der jetzigen Strategie, vor allem auch im Hinblick, wenn der Klimawandel immer, also die Folgen, immer stärker werden sollten?
11	**NA3:** Ja, natürlich gibt es da Konflikte und Probleme, dass Altbestände vielleicht noch weiter absterben. Was meines Erachtens aber nicht daran liegt, dass das insbesondere bei der Buche eine nicht-trockenheitsverträgliche Baumart wäre, sondern dass einfach die alten Bäume sich nicht so wahnsinnig schnell, wie es eigentlich soll, an veränderte Klimabedingungen und damit Standortbedingungen anpassen können. Die Buche wächst auch in Süditalien und kommt mit der dortigen Hitze und Trockenheit offensichtlich zurecht, weil sie es von klein angewohnt ist.
12	**LT:** Also ganz konkret: welche politischen Instrumente, meinen Sie fehlen noch für die Zukunft?
13	**NA3:** Es fehlt teilweise an Geduld die natürlichen Entwicklungen auch hinzunehmen und abzuwarten. Es ist zum Teil auch so ein bisschen Aktionismus, habe ich fast den Eindruck, wenn

	man jetzt an vielen Standorten Eichen pflanzt, die prinzipiell etwas trockenheitsverträglich sind, als Buchen oder wenn man jetzt eben gar auch auf Baumarten ausweicht, die eigentlich nicht hier her gehören und deren ökologische Wert sehr viel geringer ist, also der der heimischen Baumarten.
14	**LT:** Dann kommen wir jetzt zum zweiten Thema meiner Masterarbeit, und zwar, dass die Erholungsfunktion gerade bei der Stadtbevölkerung jetzt in der Zeit der Covid-19-Pandemie sich immer weiter ausgebreitet hat, also immer mehr Leute gehen in den Wald. Und welche Konflikte sehen Sie dort?
15	**NA3:** Die Konflikte sind hauptsächlich dort, wo die Leute nicht nur da wo es durchaus gewünscht ist auf den Waldwegen spazieren gehen, sondern die Wege verlassen. Da die Tierwelt beunruhigen. Auch noch nachts durch den Wald streifen mit künstlicher Beleuchtung. Das sind einfach Störungen für den Naturhaushalt, für die Tierwelt dort, die inzwischen ein Ausmaß erreichen, dass das kritisch ist. Also nichts gegen, prinzipiell gegen Besucher in dem Wald, die sollen gerne in den Wald kommen, aber sich dort, ich sage mal, regelkonform oder angemessen verhalten, dass der Wald eben nicht beschädigt wird.
16	**LT:** Und welche Lösungsansätze auf politischer Ebene sehen Sie daran?
17	**NA3:** Schwierig. Es wird wahrscheinlich nur immer wieder in der Presse und es geht darum darauf aufmerksam zu machen, dass dieses Verhalten eben den Wald schädigt und die Naturschädigt, und die Tierwelt schädigt. Verbote sind schwierig. Ich kann auch nicht überall Verbotsschilder im Wald aufstellen oder sowas. Also da hilft nur der Versuch die Bevölkerung, ich möchte fast sagen, besser zu erziehen.
18	**LT:** Dann nochmal eine allgemeine Frage: in welchen Kriterien würden Sie die Qualität des Waldes messen? Auch gerne in Bezug auf den Karlsruher Stadtwald.
19	**NA3:** Also Hauptkriterium für mich ist eigentlich, dass es eine möglichst hohe Artenvielfalt. Wobei das nicht unbedingt heißen muss, dass auf jeder Fläche gemischte Baumarten in großer Zahl vorhanden sein müssen. Es sollten nur eben keine Reinkulturen sein. Und es sollten, ich habe es ja schon mal gesagt, heimische Baumarten sein, weil die als Nahrungsgrundlage für unsere heimischen Insekten, insbesondere Schmetterlingsraupen, deutlich wertvoller sind als irgendwelche eingeführten Baumarten, an die die heimische Fauna ja eigentlich nicht angepasst ist.
20	**LT:** Aber die Umsetzung zurzeit haben Sie ja schon kritisiert? Von der Forstverwaltung.
21	**NA3:** Ja, ja.
22	**LT:** Was sind da die Schwachstellen, nochmal genau?
23	**NA3:** Ja die Schwachstellen sind, meines Erachtens, dass man auf fremdländische Baumarten ausweichen möchte. Ich kann das verstehen, weil einem nichts viel Besseres einfällt. Ich habe auch keine Patentlösung wie es im Wald wieder besser aussehen kann in Zukunft, aber ich denke, es ist der falsche Weg jetzt da Baumarten reinzuholen, die eben von geringerem ökologischerem Wert hier sind.
24	**LT:** Jetzt sind Klimawandel und die Covid-19-Pandemie sehr globale Probleme und finden Sie aber trotzdem, dass die auf lokalster Ebene angegangen werden sollten oder sollte es solche politischen Entscheidungen und Zielkonzepte höher passieren?
25	**NA3:** Sowohl als auch auf jeden Fall. Das muss auf Bundesebene, auf europäischer Ebene, auf globaler Ebene mehr passieren, aber es ist auch unabdingbar, dass auf der lokalen, kommunalen Ebene da viel gemacht wird.
26	**LT:** Jetzt gab es ja eine Reformierung der Gewaltenverteilung in Baden-Württemberg nach einem Holzkartellverfahren, also der Staatswald bei Karlsruhe wird jetzt von der Anstalt des öffentlichen Rechts ForstBW übernommen und der Körperschaftswald bleibt beim Forstamt Karlsruhe. Was ist da Ihre Meinung dazu?
27	**NA3:** Halte ich für sehr ungeschickt und ungünstig. Es wäre wirklich sinnvoll gewesen die Bewirtschaftung des gesamten Karlsruher Waldes in einer Hand zu lassen, damit einfach da kongruent gehandelt werden kann und damit auch für alle anderen, die im Wald zu tun haben ein

	Ansprechparten vorhanden ist.
28	**LT:** Aber sind da jetzt schon aktuell Konflikte aufgetreten?
29	**NA3:** Kann ich nicht wirklich sagen, aber es gibt schon, also, das Beschwerden beim Karlsruher Forstamt eingegangen sind, die sich eher auf den Staatswald beziehen z.B. Also das, das weiß ich, dass es das schon gegeben hat.
30	**LT:** Gut. Dann würde ich zum Abschluss noch fragen, ob Sie noch irgendetwas Wichtiges loswerden wollen? Generell zum Thema, was Ihnen noch eingefallen ist.
31	**NA3:** Zum Thema Waldbewirtschaftung ein Punkt vielleicht noch. Es hat früher -das war nicht gut- große Kahlflächenhiebe gegeben, die sind jetzt aber ganz verpönt und es gibt nicht mal mehr kleine Kahlflächen. Außer jetzt, wo man ganze Fichtenkulturen, die abgestorben sind flächig dann abgeholzt hat. Grundsätzlich sollte man von Zeit zu Zeit Kahlflächen im Wald zulassen oder sogar schaffen, um dort die natürliche Subsektion zuzulassen und auch Stadien wieder zu schaffen mit einer ausgeprägten Krautschicht und Strauchschicht als Übergangsphasen. Die gibt es in unseren heutigen Wäldern kaum noch. Und das sind auch Biotopflächen, die ihre Wertigkeit haben und die jetzt einfach teilweise fehlen. Ansonsten habe ich glaube ich das, was mit wichtig war, gesagt.

NA4

1	**NA4 (24:02.6 Minuten)**
2	**NA4:** Haben dann auch gleichzeitig untersucht, wie das mit den städtischen Bäumen, also in den Straßen, in den Parkanlagen, in den städtischen Gärten und so weiter ist. Und haben uns da eben auch zusammen mit der Arbeitsgruppe einer Partner-Institution kurzgeschlossen, die eben diese Straßenbaulisten erstellt haben, nachdem sie die Eignung der unterschiedlichen Bäume an diesen Standorten untersucht haben.
3	**LT:** Ich konzentriere mich ja mehr auf den Stadtwald. Finden Sie, dass da dieses naturnahe Waldmanagement zurzeit gut angewandt wird in Karlsruhe?
4	**NA4:** Also, ich finde, dass das in den letzten 30 Jahren eben doch vermehrt angewendet worden ist. Wir haben am Anfang viel, viel mehr Nadelwaldbestände gehabt, die im Kahlschlag geerntet wurden, wo die Wald-, das Wirtschaftliche, die Holzproduktion noch eine viel größere Rolle gespielt hat, als es Gott sei Dank heute der Fall ist. Wo die Wohlfahrtswirkungen im Wald letztlich doch die Erholungsfunktion, Klimaschutzfunktion, Wasserschutzfunktion und so weiter doch eine viel, viel größere Rolle spielt. Es gab dann wieder auch eine Tendenz auch im Forst Zierbäume mitzuverwenden. In Alleen z.B., gerade im nördlichen Hardtwald. Das hat man eigentlich abgelehnt. Man hat gesagt, wir legen größeren Wert auf das Pflanzen soziologisch sinnvolle Pflanzen dort verwendet werden und keine Ziergehölze aus gestalterischen Gesichtspunkten. Im städtischen Bereich sieht es wieder anderes aus, weil die Standorte in der Stadt meistens sekundäre Bodenstandorte sind und durch die Verdichtung auf den Straßen durch die Schwingungen, die der Verkehr ausübt, dieser Boden doch sehr stark verdichtet wird. Und man hat auch in der Zeit viele Untersuchungen angestellt. Man hat also Bäume, die nicht wachsen wollten, hat man ausgegraben und hat geguckt, wie ist die Durchwurzelung dort. Wo ist die Durchwurzelung hingegangen oder eben nicht? Und man hat sich auch beteiligt an größeren Projekten, die Bundesweit stattgefunden haben, unter anderem zwei andere Institutionen, wo vierzehn Städte damals mit der *Tilia* \`Pallida\` Untersuchungen gestartet haben. Da ist in allen Städten aus der gleichen Baumschule sind diese *Tilia* \`Pallida´ gepflanzt worden und es sind im Abstand von Jahren, sind dann immer die Hälfte des Wurzelraumes ausgegraben worden, um zu sehen wie die Durchwurzelung stattfindet. Und seit dieser Zeit werden auch besondere Baumschutz, Baumsubstrate verwendet, um dieses Wurzelwachstum bei den städtischen Bäumen doch positiver zu ermöglichen. Und das ist eine der wesentlichen Unterschiede, die zwischen auch der Baumuntersuchung im Forst und im Stadtbereich stattfindet.

5	**LT:** Und um jetzt nochmal auf den Stadtwald zurück zu kommen: da gibt es vom Forstamt seit einem Jahr auch ganz neu ein Konzept zur Klimaanpassung, Klimawandelanpassung. Wie würden Sie das bewerten? Kennen Sie das?
6	**NA4:** Ja, natürlich kenne ich das, ich habe das auch hier, das Buch. Da muss ich sagen, da hat man eigentlich sehr, sehr stark mit dem Forst zusammengearbeitet. Das war anfangs, als ich hier im Jahr meines Umzugs nach meinem aktuellen Wohnort kam von meinem vorherigen Wohnort aus, war das nicht der Fall. Da waren also die Förster, die wollten von den Grünen nichts wissen, die haben also ganz andere Forstwirtschaft gemacht. Und man hat immer versucht zusammen mit der Politik, also mit den Stadträten den Aspekt der Erholung, des Klimas doch viel stärker mit einzubringen. Man hat auch zusammen mit dem, damals mit einem Institut in Karlsruhe, zusammen Untersuchungen gestartet, wie die Temperaturen zwischen Wald und Innenstadtbereichen im Laufe des Tages ist, wo man dann auch darauf hingewiesen hat, dass das doch ein ganz großer Unterschied ist und der für die Durchlüftung der Stadt eine große Rolle spielt. Das ist damals alles eingeflossen worden und sehr stark dann immer mehr mit letztlich in die Forstwirtschaft eingebracht worden. Und es ist auch so, mein Vorgänger hier in der Arbeitsstelle gewesen, der in einem anderen Ort einen anderen Beruf hatte und der ist auch der Vorsitzende von einem Verein, wo Forst und Grün eigentlich doch sehr, sehr eng zusammenarbeiten.
7	**LT:** Und welche Konflikte sehen Sie bei der Klimaanpassung beim Karlsruher Stadtwald?
8	**NA4:** Ich sehe eigentlich immer noch doch Probleme, dass von der Baumartenauswahl es immer wieder, das Wirtschaftliche eine große, nach wie vor, eine große Rolle spielt. Und das hat man eigentlich jedes Mal, wenn es einen neuen Flächennutzungsplan gab, wenn man den Landschaftsplan dazu gemacht hat, hat man jedes Mal darauf gedrungen, dass die Wohlfahrtswirkung hier in dem Ballungsraum viel, viel stärker bewertet werden müssen und der wirtschaftliche Erfolg eigentlich doch etwas stärker hinten anstehen muss. Ich meine, man hat auch diese Einsätze von diesen großen Holzernten, diese großen riesen Maschinen, die hat man doch eigentlich immer sehr negativ angesprochen. Und einem war das noch viel sympathischer, als man noch die Rückpferde in den Wäldern eingesetzt hat. Das hat zwar dazu geführt, dass man die Eiben aus den Wäldern vertrieben hat, weil die die Pferde zum Teil die gefressen haben und da dran dann gestorben sind, deswegen sind die Eiben von den Förstern aus den Wäldern verbannt worden. So langsam kommen sie jetzt wieder. Aber auch das war etwas, was man eigentlich nicht so positiv gesehen hat. Man hat auch gesagt, wir wollten die Erholung gezielt machen. Also nicht überall die Leute in Massen im Wald haben. In gewissen Bereichen und die auch entsprechen herrichten und auch besonders intensive Waldränder angelegen, weil die natürlich für die Naherholung eine ganz große Rolle spielen und es da auch interessant ist die unterschiedlichsten Arten zu haben und man hat auch jedes Jahr in einem der Stadtteile solche Waldrand- und Wiesenführungen gemacht, wo man dann die Bevölkerung oder die Interessierten der Bevölkerung denen auch erläutert hat, was es für Pflanzenarten dort gibt, was für eine Bedeutung die haben insgesamt und hat da auch schon auf die Artenvielfalt hingewiesen. Auch dass Arten gepflanzt werden, die für die Vögel und für das Wild insgesamt eine Rolle gespielt haben. Aber auch, wie gesagt, Artenvielfalt insgesamt. Und bei vielen Waldführungen wird immer auch die Krautvegetation in den Wäldern dargestellt und eben auch die Bedeutung dieser Pflanzen für die Gesamtbetrachtung immer dann mit darauf hingewiesen.
9	**LT:** Also, wir haben jetzt schon das Konzept zur Klimaanpassung besprochen und die Kommunikation mit der Bevölkerung. Sehen Sie noch weitere politische Instrumente, um den Stadtwald von Karlsruhe in Zukunft vor dem Klimawandel zu schützen?
10	**NA4:** Außerdem ist 2017 vom Gartenbauamt das Freiflächenkonzept erstellt worden, was auch entsprechende Aussagen zu den Waldflächen macht und wo auch ganz besonders deutlich wird, dass die Verbindung der unterschiedlichen Grünelemente zusammengesehen werden muss, sodass Frischluftschneisen aus den Wälder heraus wirklich auch in die besiedelten Gebiete hinein fließen können und deshalb gibt es dort dieses Grünflächensystem, was die Durchlüftung der Stadt insgesamt erreichen soll und es sind in den letzten 30 Jahren auch einige größere

	Parkanlagen geschaffen worden, die diese Funktion ganz besonders auch haben.
11	**LT:** Jetzt ist es seit dem Ausbruch der Covid-19-Pandemie so, dass immer mehr Menschen aus der Stadt die Erholung im Wald suchen, vielleicht ist Ihnen das auch in Karlsruhe aufgefallen, gibt es da Konflikte, die neu auftreten?
12	**NA4:** Ja es gibt sicher Konflikte. Einmal, man hat viele Hundehalter, die dort im Wald mit unterwegs sind und die natürlich Schwierigkeiten mit dem Wildbesatz geben, aber ein größeres Problem sehe ich in der Vermüllung der Wald- und auch der Grünflächen und ich bin in den letzten Jahrenzehnten immer wieder in einem anderen Land gewesen, habe da auch gearbeitet zum Thema Biodiversität und dort gibt es eine ganz andere Auffassung. Jeder, der dort irgendetwas mitnimmt zum Essen oder Trinken, der nimmt die Verpackung selbstverständlich wieder zurück. Es gibt dort in vielen Parks keine Müllbehälter und so weiter, weil die Leute ihre Sachen alle wieder mitnehmen. Und das ist etwas, was hier bei uns nun wirklich eine Katastrophe ist. Man hat mal entlang eines Gebietes ein Jahr lang gesammelt, was dort aus den Autos alles rechts und links in die Grünflächen reingeschmissen wird und hat das mal in so große Behälter aufgestellt, um das als Zeichen mal deutlich zu machen und ich war selber erstaunt, wie viel letztlich da zusammen gekommen ist und das ist etwas, was man leider auch in den Wäldern durch diese stärkere Fluktuation in den Wäldern doch immer wieder merkt. Aber ich meine, was eigentlich immer wieder wichtig war, das war der Bezug zu der Politik, zu den Stadträten, deswegen war es immer gut, wenn auch der Forst dann Führungen im Wald gemacht hat, meine Arbeitsstelle war dann auch dabei, da waren die Stadträte dabei und da konnte man dann auch dabei das denen deutlicher machen. Das war am Anfang war das also ein bisschen konfliktbeladener, weil man häufig dann gegenüber dem Forst doch angesprochen hat, dass man doch eine naturnähere Pflege und Waldunterhaltung haben will und weniger diese Koniferen Monokulturen und man hat dann vom Forst immer wieder die wirtschaftlichen Gesichtspunkte, hat dann immer deutlich gemacht wie viel Geld letztlich da in den Stadthaushalt fließt. Und man war immer der Meinung, das ist eigentlich das Grün in den Ballungsgebieten, hat hauptsächlich Wohlfahrtsfunktionen und die wirtschaftliche Funktion, die darf nicht diese Rolle spielen. Und ich meine, was auch diese großen Waldbereiche heute, wenn man z.B. durch eine Region in einem anderen Bundesland fährt und dort ganz Täler Koniferenwälder abgestorben sieht, dann kann man gut sehen, dass die Forstwirtschaft doch -auch zum Teil die Private- ziemlich daneben gelaufen ist. Weil man eben, und das hat auch damals vor 200 Jahren dazu geführt, man wollte möglichst schnell aufforsten und deswegen hat man die Kiefer in größerem Umfang in den Hardtwald gebracht und dort steht sie heute zum Teil noch.
13	**LT:** Und wenn jetzt für die Forstleute die Finanzierung, also die Waldwirtschaft im Mittelpunkt steht, wie könnte man denn sonst politisch den Wald finanzieren anstatt von Holzproduktion?
14	**NA4:** Der Wald, der muss, meiner Ansicht nach, durch die öffentliche Hand finanziert werden, weil die Wohlfahrtswirkungen so wichtig geworden sind und es wird nicht anders gehen und man wird auch den privaten Forstbetrieb, den wird man auch unterstützen müssen, genauso wie man auch die Landwirtschaft mit Subventionen unterstützt, weil sie auch Wohlfahrtswirkungen neben ihrem eigentlichen Ertrag produzieren. Und ähnlich ist da mit dem Wald. Anders kann es eigentlich gar nicht gehen, es sei denn man geht her und kassiert Eintritt. Wie man das ja zum Teil in den Parks versucht hat oder gemacht hat. Nur das führt letztlich auch leider zu unschönen Auswirkungen. Das sieht man in meinem Wohnort in einem Park, da ist der Eintritt, dadurch das ein Teil Freizeiteinrichtung drin ist, heute so teuer, dass jemand, der in den einen Teil des Parks geht oder in den anderen Teil des Parks gehen will, der zahlt den hohen Eintritt und das sind dann weniger und dadurch sind die Leute ausgeschlossen. Wobei gerade dort für den Stadtteil meines Wohnortes das eigentlich die Grünfläche ist, die für die Leute dort eine ganz große Rolle spielt und das sieht man dort, dass der große Spielplatz für die Kinder, der wird unwahrscheinlich dort angenommen. Das ist so, das, wenn man diesen Weg geht, schließt man viele Leute aus. Und das kann es nicht sein.
15	**LT:** Nach welchen Indikatoren würden Sie die Qualität des Stadtwaldes messen?
16	**NA4:** Die würde ich einmal danach messen wie der Gesundheitszustand des Waldes ist und dann

	welche klimatischen Funktionen erfüllt werden und natürlich auch die Artendiversität. Die würde auch bei mir eine ganz große Rolle spielen, denn wenn man sich mal so einen Fichtenmonobestand anschaut. Dort steht dann nur die Fichte, untendrunter ist es dann praktisch eine biologische Wüste, da ist also gar nichts. Während in einem vernünftigen Mischwald, wo die unterschiedlichen Altersstufen zusammenkommen, dort sehen Sie auch Schmetterlinge, dort sehen Sie Libellen, da sehen Sie Vögel und hin und wieder springt da ein Reh über den Weg. Und das ist natürlich, so sollte eigentlich so ein städtischer Wald letztlich dann aussehen. Aber man sollte auch bei den städtischen Wäldern die Wegeführung so wählen, dass es ruhigere Bereiche gibt und dann auch Bereich gibt, die zur Erholung attraktiv sind, dass da möglichst die Leute sich aufhalten und nicht in den ganzen Wald letztlich dann so mit Beschlag belegen, dass dort überall rumgelaufen wird und das bringt dann doch erhebliche Schäden mit sich.
17	**LT:** Jetzt sind der Klimawandel und die Covid-19-Pandemie sehr globale Probleme. Finden Sie, dass die sich am besten auf kommunaler Ebene angehen lassen oder sollte das mehr international oder national geregelt werden?
18	**NA4:** Das sehe ich auf verschiedenen Ebenen. Das muss einmal für die ganze Bundesrepublik generell und dann über die Länder und dann aber zu den einzelnen Kommunen. Was ich schlecht finde hier in dem Bundesland, wo ich wohne wie die Entwicklung so in den letzten Jahren gegangen ist. Dieses hin und her und her und hin. Das hat dazu geführt, dass wir jetzt wieder staatliche Behörden innerhalb der Stadt haben. Direkt daneben eine städtische Fläche haben, denn wir haben hier ungefähr 50/50% hier und 2% privaten Wald und das ist nicht besonders sinnvoll. Gerade in den Ballungsgebieten, wo diese Erholungsfunktion, die Klimafunktion und so weiter so eine große Rolle spielen, da sollte das eigentlich möglichst schon in einer Hand sein und nicht so abgedrängt nebeneinander herlaufen. Also das finde ich leider in dem Bundesland, wo ich wohne zurzeit nicht optimal. Was für mich immer noch sehr interessant ist, dass ist den Klimawandel als Prozess zu sehen. Wir suchen heute nach Baumarten, die auch in 30, 40, 50 Jahren noch hier wachsen können. Ich habe ein bisschen Beziehungen nach einem anderen Standort, mit dem Kollegen bin ich befreundet und der Vorgänger, der wollte mal ganz schlau sein und hat einen Teil Palmen gepflanzt. Das ging ein paar Jahre gut und dann kam ein Frost und dann waren die alle hinüber. Und das muss man wirklich alle mit überlegen und muss eben mit dem Prozess eigentlich mitgehen. Und entsprechende Baumarten pflanzen auch dass man die anderen die Tierwelt mitberücksichtigt. Interessant ist ja, dass die heimische Eiche für viel mehr Insekten interessant ist, als z.B. die amerikanische Roteiche. Und das sind Gesichtspunkte, die müssen jeweils mit dabei berücksichtigt werden. D.h. es wird auch nicht den typischen Baum für die nächsten 50 Jahre geben, sondern es wir auch da eine Bandbreite geben müssen, aus der man wählt und möglichst eine Mischung. Das haben wir auch hier in der Stadt überall gesehen, wenn dann plötzlich eine neue Krankheit in die Bäume herkommt. Z.B. die Platane oder auch bei der Kastanie, dann fällt sofort alles aus. Wobei ich schon dazu neige in den einzelnen Straßen schon durchgängig eine Baumart zu pflanzen, um auch eine bessere Orientierung in der Stadt zu finden. Wenn ich hier irgendwo in eine Straße reinfahre, wo durchgängig Platanen sind, dann weiß ich gleich, jetzt bin ich dort oder dort. Also eine bunte Mischung finde ich nicht so gut, aber doch eine starke Mischung in den verschiedenen Straßen, wobei immer möglichst der Standort entsprechend dann auch berücksichtigt werden muss. Und das ist im Wald letztlich genauso, ich meine, der Schädlingsaspekt spielt auch im Wald eine Rolle, das fing historisch auch an mit der Ulme, jetzt haben wir es mit der Esche und vielen anderen Dingen, denn durch den Klimawandel kommen auch entsprechende Schädlinge entsprechend stärker auch hier. Und die Nützlinge, das Verhältnis Nützling zu Schädling verändert sich auch fortlaufend.
19	**LT:** Gibt es zum Abschluss noch irgendetwas, was Sie im Zusammenhang sagen möchten? Sonst wären wir schon am Ende.
20	**NA4:** Was ich nochmal betonen möchte, dass man unter diesen Wohlfahrtswirkungen nicht nur eine Art sieht, nicht nur Parks oder nur Wälder oder sowas, sondern wirklich das Grünflächensystem insgesamt zusammen. Denn es gibt Gutachten z.B. für New York, wenn man da sämtliche Flachdächer begrünen würde, dann würde man nicht nur die

	Temperaturentwicklung besser in den Griff kriegen, sondern man würde auch diese Starkregen, die dort runtergehen und die dann jedes Mal an 500 Stellen zu Überschwemmungen führt, das würde man dann letztlich auch sinnvoll regeln können und hätte

A1

1	**A1 (23:34.8 Minuten)**
2	**LT:** Was bedeutet für Sie naturnahes Waldmanagement?
3	**A1:** Gut. Naturnahes Waldmanagement, das leitet sich natürlich in erster Linie ab von der Konzeption 'naturnahe Waldwirtschaft', dass man schon seit über 30 Jahren anwendet. In der Landesforstverwaltung, bzw. im Staatswald. Es ist in erster Linie eine standartgerechte Baumartenwahl und da gehört natürlich die Klimaeignung jetzt ganz aktuell mit dazu. Man hat da auch entsprechende Werkzeuge da dafür, die eine Forschungseinrichtung bereitstellt. Wie Baumarteneignungskarten, nach bestimmten Klimaszenarien etc. pp. Also da ist man, denke ich, recht gut aufgestellt. Natürlich immer mit einem gewissen Unsicherheitsfaktor. Irgendwo ist es ja schon ein Blick in die Glaskugel. Also standartgerechte Baumartenwahl steht da sicherlich ganz mit im Vordergrund, dann aber natürlich auch möglichst das Ausnutzen der natürlich Verjüngung. Das Genreservoir entsprechend auszunutzen. Das ist insbesondere gerade wichtig bei der Verjüngung der Buchenaltbestände. Die ja im Moment stark kränkeln. Und da hofft man schon, dass da mal bei der natürlichen Verjüngung der Genpool so groß und ausreichend ist, dass doch die nächste Waldgeneration dann doch etwas klimaangepasster dann entwickeln kann. Gut. Dann natürlich auch entsprechend angepasste Schalwildbestände. Bei uns ist das eher in erster Linie das Rehwild. Das angepasste Wildbestand ist notwendig auch, um eine standartgerechte Wiederbewaldung auf natürlicher Basis, auf Naturverjüngung zu erreichen. Und wenn ich eben von Wildstand oder -stände spreche oder Wildverbiss ist natürlich auch der selektive Verbiss ganz entscheidend, das sieht man immer wieder. Und ein weiterer Faktor der naturnahen Waldwirtschaft ist natürlich auch der Aufbau von gemischten Wäldern. Und das ist natürlich auch in Anbetracht des Klimawandels ganz entscheidend. Dass wir nicht nur auf eine Baumart setzten, sondern eben auf mehrere Baumarten. In Form von Mischung oder Mischungsformen und wenn wir natürlich, natürlich Verjüngen wollen, sind dann dort entsprechend angepasste Wildbestände ganz entscheidend.
4	**LT:** Welche Konflikte sehen Sie da jetzt gerade? Bei der jetzigen Stadtwaldverwaltung und
5	**A1:** Gut es ist schon so, die Arbeitsstelle betreut ausschließlich Staatswald. Und der Großteil dieser Staatswälder bejagt oder bewirtschaftet man in eigener Regie. Also da legt man die Bejagungsziele fest und bejagt die Flächen dann entsprechend. Probleme bereiten sich dort, sagen wir mal so, wo aufgrund von enger Verzahnung, von Staatswald und nicht-Staatswald man jagdrechtlich nicht das Sagen hat. Und da zeigen sich dann auch die Probleme. Mit der Konsequenz, dass man eben einen sehr hohen Aufwand betreiben muss beim Schutz der Kulturen oder der Verjüngungsflächen. Was schon einen erheblichen Kostenaufwand darstellt.
6	**LT:** Und Konflikte bei der Baumartenauswahl? Gibt es die auch?
7	**A1:** Konflikte bei der Baumartenauswahl sehe ich dort nicht. Also wie gesagt, man setzt in erster Linie auf heimische Baumarten, von denen man ausgeht, dass die klimastabil sind. Bei uns in unserem Naturraum in erster Linie ist es die Traubeneiche. Gefolgt von *Sorbus*arten, wie Elsbeere, Speierling oder auch andere Mischbaumarten: Kirsche, Feldahorn zählt dazu. Das ist, sagen wir mal, der erste Bereich. Und der zweite Bereich: man setzt aber auch auf bewährte, anerkannte Fremdländer, fremdländische Baumarten. Das ist bei uns die Douglasie, es ist in Teilen auch die Roteiche. Aber da sind durch die Zertifizierung, insbesondere FSC, der gesamte Staatswald ist ja FSC-zertifiziert, da sind schon sehr stringente Regularien vorgegeben. Mit dem Anteil dieser Fremdländer, mit der Mischungsform, also man darf die nicht großflächig Beimischen, sondern

	maximal horstweise, das sind 0,3 ha. Dann hat man aber auch eigene Vorgaben, die die Verwaltung sich selber auferlegt hat im Zuge der Gesamtkonzeption Waldnaturschutz. Nach dieser Konzeption dürfen maximal 49% einer Bestandes Fläche mit Fremdländern verjüngt werden. Also das ist schon sehr stringent. Da könnte, würde FSC sogar mehr, heute mehr ermöglichen, aber das ist eine Eigenverpflichtung von der Verwaltung, dass man eben sich bei der, beim Anbau von Fremdländern zurückhält. Nach FSC gehört auch die Edelkastanie mit dazu, auch das ist eine nicht-heimische Baumart, nach FSC. Auch die Walnuss, auch eine klimaangepasstere oder klimastabilere Baumart. Zählt auch als eine nicht-heimische Baumart, die dann entsprechend nach diesen Restriktionen belegt sind.
8	**LT:** Und welche weiteren politischen Instrumente braucht es für die Zukunft, um die Stadtwälder oder Staatswälder bei Karlsruhe, gegen den Klimawandel zu schützen?
9	**A1:** Also ich denke, neben der jagdwirtschaftlichen Situation, neben der Jagdwirtschaft, was ich bereits angesprochen habe, und da muss natürlich gerade ein kommunaler Waldbesitzer, der ja auch die Regularien und die Ziele vorgibt für die jagdliche Bewirtschaftung, das kann er ja dann auch entsprechend in den Pachtverträgen, in den Jagd-Pachtverträgen festhalten. Das ist der eine Bereich. Aber der andere Bereich ist natürlich auch, dass ausreichend Ressourcen für den Umbau der Wälder in Richtung klimastabile Wälder zur Verfügung stehen. D.h. auf der einen Seite sind es finanzielle Mittel und das sind große Beträge, die da zusammenkommen. Also ein Hektar Eichenkultur, also in gemischter Form natürlich, das sind alles Mischwälder, die künstlich begründet werden muss, also über Pflanzungen mit Schutz, da sind wir relativ schnell mal bei zwischen 20.000 Euro das Hektar. Also es müssen finanzielle Mittel bereitgestellt werden und natürlich auch Ressourcen personeller Art. Insbesondere auch das entsprechende Fachpersonal in Form von Försterinnen und Förster, die das ganze betreuen. Auch entsprechende Forstwirte, die die Arbeit draußen machen. Natürlich kann man auch auf fremde Ressourcen zurückgreifen, sprich den Forstunternehmen, aber nichtdestotrotz braucht man einen Stamm von eigenen Beschäftigten, die draußen, die Ortskenntnisse haben, die auch entsprechend qualitativ hochwertige Arbeit macht. Und die auch zeitnah zur Verfügung steht.
10	**LT:** Und die finanziellen Mittel: woher würden die dann kommen?
11	**A1:** Die finanziellen Mittel kommen bei der Staatswaldverwaltung aus dem Landeshaushalt. Und man erwirtschaftet diese Mittel indem man Holz verkauft. Ganz einfach. Im Moment gibt es ein Sonderprogramm beim Land für die Wiederbewaldung. Das sind im Moment drei Millionen im Jahr, drei Millionen Euro für das gesamte Land, also für ForstBW, für den Staatswald im Land Baden-Württemberg. Wenn wir diesen Sondertopf nicht haben, den haben wir jetzt erst frisch bekommen, dann müssen wir die Kosten für die Wiederbewaldung aus der Holzproduktion und aus dem Holzverkauf decken.
12	**LT:** Das zweite Thema meiner Masterarbeit ist noch die Erholungsfunktion des Waldes und dass jetzt durch die Covid-19-Pandemie immer mehr Menschen in den Wald gehen und diese Erholungsfunktion gesteigert genutzt wird. Sehen Sie da Konflikte bei der aktuellen Stadtwaldverwaltung und der größeren Nachfrage des Waldes bei der Stadtbevölkerung?
13	**A1:** Also den größten Konflikt, den ich im Moment sehe, machen sich die Erholungssuchenden selber. Wenn Sie gerade mal in das Spannungsverhältnis Radfahrer, Mountainbiking, E-Biking und fußläufig unterwegs, Fußgänger, Wanderer, die das fußläufig unterwegs sind. Der Konflikt ist gewaltig. Vor allem dann, wenn, sagen wir mal, die eine Gruppe sich nicht an die Spielregeln hält und dann schmale Pfade befährt, was sie eigentlich nicht dürften, aber das ist sehr schwer zu regulieren. Also die Konflikte machen sich die Erholungssuchenden selber. Der zweite Konflikt, der sich zunehmend zeigt ist auch der Konflikt mit der jagdlichen Nutzung. Die Erholungssuchenden sind leider nicht mehr nur in normalen Tageszeiten unterwegs, morgens bis abends, sagen wir mal so, sondern leider auch bei Nacht oder in der späten Dämmerung. Und das sind eben Zeiten in denen eben das Wild, das Rehwild, Schwarzwild aktiv ist und dadurch nicht nur die Bejagung erheblich erschwert wird, sondern auch das Wild in seinen natürlichen Nahrungsgewohnheiten und Abläufen gestört fühlt und dadurch wieder gezwungen wird mehr Forstpflanzen konzentriert aufzusuchen und dort entsprechend ein größerer, Schad-,

	Wildverbiss vorzunehmen. Da sehe ich schon einen Konflikt. Ansonsten, ein dritter Konflikt, was sich zur Zeit abzeichnet, gerade auch im Bereich vom Erholungswald: die Verwaltung des Staatswaldes bewirtschaftet ja große Teile des Erholungswaldes, d.h. der Wald dient nicht nur der Erholungsfunktion, sondern er dient auch der Nutzungsfunktion und viele Waldbesucher, Erholungssuchenden stören sich da dran. Viele leben einfach noch in der Vorstellung: ein Erholungswald, der ist nur für die Erholungssuchenden da und alle anderen Funktionen müssen sich dem unterordnen. Und das ist halt teilweise schwierig. Man nimmt da zwar massiv Rücksicht, aber das fängt schon an im Winter, wenn eben der Holzeinschlag ist, da sind dann auch mal die Wege verschmutzt. Die Wege werden dann zwar wiederhergerichtet, aber viele Waldbesucher stören sich da dran, wenn sie eben mit normalen Schuhen dann durch den Wald laufen und dann eben schmutzige Schuhe bekommen. Oder sie stören sich rein an dem Anblick von Maschinen. Also da ist schon ein gewisses Konfliktpotenzial da: Erholungsnutzung auf der einen Seite, forstliche Nutzung auf der anderen Seite. Also das würde ich jetzt mal also drittes Konfliktpotenzial ansehen. Einer steigernden Erholungsnutzung durch Corona-Zeit.
14	**LT:** Und welche politischen Lösungsansätze sehen Sie dafür?
15	**A1:** Also ich denke, auf der einen Seite, ganz wichtig ist die Aufklärung, Öffentlichkeitsarbeit. Egal welche Konfliktpotenziale das sind. Und das andere ist eben eine ganz gezielte Besucherlenkung. Andere Möglichkeiten sehe ich als schwierig an: wir können keinen Wald sperren, wir können den Leuten auch nicht verbieten Tages- und Nachtzeiten bestimmte Waldgebiete nicht aufzusuchen. Insofern: Aufklärung, Schaffung von Akzeptanz. Ich denke das ist der einzige vernünftige Weg.
16	**LT:** Dann noch eine allgemeinere Frage: in welchen Kriterien würden Sie die Qualität des Stadtwaldes messen?
17	**A1:** In welcher Hinsicht? In Richtung Erholungsfunktion oder?
18	**LT:** Oder auch die Nutzungsfunktion oder Biodiversitätsfunktion.
19	**A1:** In welchen Kriterien? Also das geht vor allem in Richtung natürlicher Faktoren, wie Baumartenzusammensetzung oder Struktur. So in die Richtung, oder wie meinen Sie das?
20	**LT:** Ganz frei können Sie das eigentlich beantworten. Was ist für Sie ein hochqualitativer Wald?
21	**A1:** Also ein hochqualitativer Wald ist in erster Linie ein stabiler Wald, also ein gesunder Wald, weil dieser nämlich allen Funktionen, allen Waldfunktionen am besten gerecht wird. Jetzt ganz konkret in Richtung Erholung gedacht, ist natürlich ein gemischter Wald wichtig. Und zwar nicht nur Laubbäume, sondern auch Nadelbäume, weil die eben wintergrün sind. Es gibt gerade, sagen wir mal, über die Wintermonate schon nochmal andere Waldbilder. Deshalb ist es eben auch wichtig, dass wir bei der Artenauswahl die Nadelbäume auch nicht ganz vergessen. Wir sehen, dass wir auch wieder vermehrt Eiben pflanzen. Die Eibe ist ja eine heimische Baumart, also auch Nadelbaumart, die auch recht klimastabil ist. Auch auf die greifen wir jetzt zunehmend mehr zurück neben der Douglasie wohlgemerkt. Weitere Kriterien: gut, natürlich ein entsprechendes Erschließungsnetz, dass da auch entsprechende Möglichkeiten da sind, dass der Wald aufgesucht werden kann. Von Fahrradfahrern, von Wanderern, Spaziergängern, etc. Dann auch entsprechende andere Infrastruktur für die Erholung von Wanderparkplätzen bis hin zu Bänken, Unterstellmöglichkeiten. Also Schutzhütten etc. Auch das wäre wichtig. Wie z.B. auch bei uns am Arbeitsort, im stadtnahen Erholungswald. Das ist der große Wald innerhalb des Arbeitsgebiets, wo wir auch entsprechende Schutzhütten haben, sanieren. Man hat Infotafeln, man hat Waldlehrpfade, etc. Auch das würde ich jetzt mal unter Infrastruktur sehen. Aber wichtig denke ich, ist ein gemischter Wald, ein abwechslungsreicher Wald, auch nicht nur ein dunkler Wald, sondern da müssen auch mal lichtere Partien da sein, wo die Menschen dann auch wieder Sonne tanken können. So würde ich jetzt einmal vor allem mit dem Fokus eines Erholungssuchenden einen qualitativ hochwertigen Erholungswald charakterisieren.
22	**LT:** Wir hatten jetzt den Klimawandel angesprochen und die Covid-19-Pandemie und das sind ja beides sehr globale Probleme. Und gleichzeitig geht es um den Karlsruher Staatswald: meinen Sie, dass das besser ist solche großen Probleme auf niedrigster Ebene zu lenken oder sollten da

	höhere Vernetzungen stattfinden auf internationaler oder nationaler Ebene?
23	**A1:** Gut ich denke mal, wenn wir jetzt einmal von den Ursachen absieht von Klimawandel und Pandemie, ich meine gut, dass können wir örtlich, das Problem können wir eigentlich nicht lösen. Aber ich denke, jetzt rein was forstwirtschaftliche oder das forstliche Handeln betrifft, meine ich, müssen wir das auf regionaler Ebene lösen. Da sind einfach die Verhältnisse zu indifferent, dass man da irgendwelche nationalen oder internationale Waldprogramme schafft dafür. Gut, okay. Die können gewissen Rahmen vorgeben aber letztendlich müssen wir es vor Ort klären, vor Ort lösen. Wir müssen doch vor Ort Lösungsansätze suchen und umsetzten.
24	**LT:** Und wenn es dann solche Reformierung in der Gewaltenlenkung gibt, wie jetzt, dass z.B. der Staatswald jetzt von der Anstalt des öffentlichen Rechts ForstBW übernommen wurde und jetzt in Karlsruhe das Forstamt nur den Körperschaftwald verwaltet. Was ist da Ihre Meinung dazu?
25	**A1:** Das sehe ich jetzt völlig unkritisch. Weil letztendlich ForstBW ein sehr enges Korsett von Vorgaben hat in Richtung Zertifizierung. Ich weiß nicht, ob der Stadtwald Karlsruhe FSC-Zertifiziert ist, das weiß ich jetzt nicht. Der Staatswald ist es. Wenn Sie sich mal mit den Standards auseinandersetzten, sehen Sie sehr schnell, dass mit diesen Kriterien sowohl ökonomischen, ökologischen und sozialen Kriterien sehr weitgehend Rechnung getragen wird. Dann kommt natürlich dazu, das haben wir anfangs angesprochen, die naturnahe Waldwirtschaft. Ich glaube nicht, dass im Stadtwald, also im kommunalen Stadtwald Karlsruhe da anders gewirtschaftet wird. Man trägt im Staatswald bestimmten Funktionen, im Besonderen Rechnung, gerade wenn es um den Erholungswald geht. Dort wird man sicherlich anders wirtschaften, oder wirtschaftet man anders, als, ich sage mal, in ländlichen Räumen. Also ich kann mir nicht vorstellen, dass jetzt die kommunale Forstverwaltung in Karlsruhe ihre Wälder grundsätzlich anders bewirtschaftet, als die Verwaltung des Staatswaldes.
26	**LT:** Also wie bewerten Sie dann jetzt die Auswirkungen des Holzkartellverfahrens? Das ist ja auch erst seit 2020 so, oder?
27	**A1:** Ja gut, Die Auswirkung vom Kartellverfahren ist, dass eben jetzt eine staatliche Forstverwaltung dadurch, dass sie für alle, also in der Vergangenheit, für alle Waldbesitzarten - Kommunal-, Privat- und Landeswald- in einer Hand das Holz vermarktet hat, ein Vertriebskartell darstellt. Das ist ja so auch nie widerlegt worden auch nicht von höchst richterlichen Entscheidungen. Also d.h. es gibt oder es gab ein Vertriebskartell und damit war eben in den alten Organisationsstrukturen die Vermarktung vom Nadelstammholz, um das ging es ja letztendlich, ein Vertriebskartell und dementsprechend musste man andere Organisationsstrukturen finden, um zu vermeiden, dass da ein Vertriebskartell stattfindet. Und da war eben die Gründung oder die Herauslösung der Staatswaldbewirtschaftung in eine Anstalt öffentlichen Rechts eine wesentliche Maßnahme dagegen. Aber ich will mal so sagen, ob jetzt die Verwaltung des Staatswaldes das Holz aus dem Staatswald vermarktet und das städtische Forstamt das Holz aus dem Stadtwald vermarktet, das hat sicherlich keinerlei Auswirkungen auf die Gewährleistung der Waldfunktionen. Unabhängig davon, ob es sich jetzt um Erholungsfunktionen oder um Schutzfunktionen handelt. Man wird deshalb nicht mehr und nicht weniger Holz einschlagen, unabhängig jetzt davon, ob man jetzt eigenständig als eigenständige Anstalt öffentlichen Rechts das Holz vermarkten oder ob es das städtische Forstamt vermarktet.
28	**LT:** Dann sind wir auch schon beim Abschluss. Gibt es noch irgendetwas, was Sie im Zusammenhang mit dem Interview loswerden wollen oder was Ihnen noch eingefallen ist?
29	**A1:** Nein.

A2

1	**A2 (15:59.8 Minuten)**
2	**LT:** Zum Einstieg würde ich einmal fragen, ob Sie zurzeit durch Ihre Arbeit mit dem Thema Karlsruher Stadtwald in Berührung sind?

3	**A2:** Ja, also wir sind zuständig für Naturschutzgebiete oder auch für europäische Schutzgebiete, also FFH-Gebiete oder Vogelschutzgebiete und das gibt es natürlich auch Überschneidungen, der Wald in meinem Arbeitsort z.B., der ist teilweise auch FFH- und zu anderen Teilen auch Vogelschutzgebiet.
4	**LT:** Und was bedeutet für Sie das naturnahe Waldmanagement?
5	**A2:** Das naturnahe Waldmanagement. Also im Idealfall würde es für mich bedeuten, dass man auch natürliche Prozesse im Wald zulässt, also z.B. jetzt sehe ich eben im Rahmen des Klimawandels und eben auch der Absterbe Erscheinungen der Wälder sehr große Bemühungen dort mit fremdländischen Baumarten auch wieder aufzuforsten. Und das sehe ich kritisch. Ich würde mir wünschen, dass man hier zum einen auch gewisse Prozesse einfach mal zulässt. Absterbe Erscheinungen, Naturverjüngung. Dass man auch Vorwaldbaumarten wie Birke oder Salweide oder ähnliches einfach wie es im natürlichen Ablauf wäre, jetzt wieder die Flächen besiedeln lassen würde. Und genau, das würde ich mir wünschen im Rahmen der naturnahen Waldwirtschaft.
6	**LT:** Das haben Sie ja jetzt schon fast als aktuellen Konflikt ausgedrückt, die Baumartenauswahl oder Naturverjüngung. Was für weitere Konfliktfelder sehen Sie denn noch in der Klimaanpassungsstrategie von Karlsruhe?
7	**A2:** Ja, also wir haben ja auf der einen Seite das Thema Insektensterben sehr, sehr präsent, dass überall in den Medien ist und diese Klimaschäden des Waldes, die sind für viele Menschen natürlich gravierende Schäden und natürlich ist es auch langfristig die Frage, wie geht es weiter mit den Wäldern? Werden wir weiterhin Buchenwälder haben? Und ähnliches, aber es ist auf der anderen Seite im Augenblick auch eine große Chance der biologischen Vielfalt, also Totholz ist sicherlich der Faktor, der die Insektenvielfalt im Wald enorm beeinflusst. Und insofern sehe ich die Entwicklung zweischneidig. Also ich sehe auch gewisse positive Effekte, auch dadurch, dass wir gerade im Wald große dynamische Prozesse haben, die so in der Vergangenheit nicht da waren, auch nicht geduldet wurden, und die auch die biologische Vielfalt erhöhen und da würde ich mir durchaus ein wenig mehr Gelassenheit wünschen mit diesen Prozessen auch entsprechend umzugehen. Ich habe ja vorher schon angedeutet auch Wiederbewaldung durch forstlich nicht so interessante Baumarten fände ich jetzt im Stadtwald völlig akzeptabel, weil der ja mehr die Erholungsnutzung hat und weniger jetzt die Wertholzerzielung.
8	**LT:** Und welche weiteren politischen Instrumente sehen Sie für Lösungsansätze in der Klimaanpassungsstrategie?
9	**A2:** Welche weiteren politischen Lösungsansätze? Also vielleicht können Sie nochmal ein bisschen präzisieren, worauf Sie hinaus wollen mit der Frage?
10	**LT:** Das naturnahe Waldmanagement ist ja ein politisches Instrument, würde ich sagen. Und ob Sie auch innerhalb dessen noch weitere Instrumente, oder auch Strategien sehen? Oder auch daneben noch?
11	**A2:** Genau also die Klimaanpassungsstrategie, das ist ja zum Teil auch sehr Fachkonzepte, die auch erarbeitet werden, also z.B. landesweit werden da Fachkonzepte erarbeitet und eben im Bezug auf die Stadt Karlsruhe ist mir jetzt gar nicht bekannt: Hat die Forstverwaltung Karlsruhe eine eigene Klimaanpassungsstrategie für den Wald, die politisch diskutiert wurde im Gemeinderat? Das ist mir jetzt z.B. nicht bekannt, ob das als politische Fragestellung im Gemeinderat thematisiert wurde.
12	**LT:** Also das Forstamt hat ein Konzept zur Klimaanpassung für den Stadtwald Karlsruhe letztes Jahr veröffentlicht. Und da wurde eben dieses naturnahe Waldmanagement beschrieben und auch nochmal Klimaprognosen und welche Überlegungen dazu gerade diskutiert werden.
13	**A2:** Okay, das ist mir jetzt im Detail nicht bekannt. [05:35.0 - 05:45.5 nicht transkribiert, wegen Anonymität] Aber einfach, wenn es jetzt um Inhaltliches geht, hatte ich ja schon einiges angemerkt, wo ich finde, da könnte man anders vorgehen, also dieser Druck möglichst rasch aufzuforsten, die Flächen möglichst rasch zu räumen. Da würde ich mir eine andere Herangehensweise wünschen.

14	**LT:** Und wäre da nicht die Finanzierung ein Problem?
15	**A2:** Also nein, also was ja finanziell was Geld kostet ist ja die Flächen zu räumen und die Flächen neu zu bepflanzen, den Aufwuchs durch entsprechende Pflege frei zu halten. Das ist ja das, was viel Geld kostet. Die gesamte Jungbestandpflege ist ja im Forst eigentlich das, was sehr viel Kapital bindet. Und wenn ich Naturentwicklung zulasse, dann habe ich eigentlich natürlich weiterhin die Verkehrssicherungspflicht, die kostet natürlich auf jeden Fall, aber die ändert sich ja nicht. Egal wie ich arbeite. Aber ansonsten würde ich eher Kosten sparen damit.
16	**LT:** Dann würde ich zum zweiten Thema meiner Masterarbeit kommen und zwar ist das die politische Lenkung im Hinblick auf die Erholungsfunktion, die ja jetzt immer stärker nachgefragt wird, vor allem auch in Zeiten der Covid-19-Pandemie gehen ja mehr Menschen aus der Stadt in den Wald. Und sehen Sie da Konflikte, die jetzt auftreten?
17	**A2:** Also ganz grundsätzlich sehe ich auch diese vermehrte Freizeitnutzung im Wald. Was mir auffällt ist natürlich auch, Dinge wie Mountainbiketrails und ähnliche Dinge, die im Wald angelegt werden, die plötzlich auftauchen, der Wald als Spielplatz. Dass da alle möglichen Dinge gebaut werden: plötzliche große Hängematten, Seile und was weiß ich was. Das ist aus meiner Sicht erstmal grundsätzlich nicht das riesige Problem, es ist dort ein Problem, wo wir besonders schutzbedürftige Gebiete haben und da findet, also da ist es schwierig offensichtlich, dass die Bevölkerung akzeptiert, dass es Bereiche gibt, wo ein freies Betretungsrecht besteht und Bereiche, wo das eben nicht der Fall ist. Und da fehlt tatsächlich auch geeignete Konzepte, die die Menschen dann auch aus diesen Bereichen rauszuhalten. Ich denke jetzt gerade an die Naturschutzgebiete, z.B., Naturschutzgebiet im Arbeitsbereich hat ja auch Waldanteile mit drin. Da ist es genau wie in anderen Bereichen eben auch. Da sieht man eben auch Leute mit freilaufenden Hunden und Leute teilweise abseits der Wege. Und das ist tatsächlich aus meiner Sicht auch ein Problem, das zugenommen hat und das, für das wir aktuell noch keine richtige Lösung haben.
18	**LT:** Also, politische Lösungswege oder Lenkungen, Instrumente würden Ihnen dazu jetzt auch nicht einfallen oder werden da schon welche diskutiert?
19	**A2:** Naja also. Politisch könnte man z.B. bei der Prioritätensetzung bei Personal z.B. denken, dass man Ranger für solche Gebiete einstellt. Man könnte die kommunalen Ordnungsdienste auch stärker für solche Arbeiten mit heranziehen, dass da eben nicht nur Falschparker kontrolliert werden, sondern eben auch mal in den Schutzgebieten Kontrollen stattfinden. Das ist tatsächlich immer eine Frage der personellen Ressourcen und der Prioritätensetzung dort und die könnte man natürlich auch politisch vorgeben.
20	**LT:** In welche Kriterien würden Sie die Qualität des Karlsruher Stadtwaldes messen?
21	**A2:** In welchen Kriterien würde ich die Qualität messen?
22	**LT:** Den Hinblick können Sie sich aussuchen, also das ist eine ganz offene Frage.
23	**A2:** Also es geht jetzt nicht um eine Bewertung von meiner Seite, sondern Kriterien, nach denen ich den Stadtwald gerne bewerten würde? Ja also, wie gesagt, ich bin Biologe, Naturschützer, mich interessiert immer die biologische Vielfalt und die Besonderheiten. Also von daher würde ich Wälder gerne nach ihrem Wert auch für die biologische Vielfalt auch beurteilen.
24	**LT:** Und jetzt sind ja Klimawandelfolgen und so Situationen, wie die Covid-19-Pandemie, globale Probleme. Finden Sie es vorteilhaft, wenn die auf lokalster, kleinster Ebene gelenkt werden oder gelöst werden? Oder sollte das dann auf eher höheren Ebenen angegangen werden?
25	**A2:** Ja also das eine schließt das andere ja nicht aus. Also, ich denke, was sicherlich keinen Sinn macht, ist dass sich jetzt jede kleinste Einheit mit grundsätzlichen Fragen beschäftigt. Wie werden bestimmte Baumarten auf das Klima reagieren? Wie reagiert auch sozusagen unsere heimische sonstige Tier- und Pflanzenwelt auf Veränderung der Baumarten Zusammensetzung? Das sind, glaube ich, Dinge, die man besser auf Landes- oder Bundeseben auch mit entsprechenden Fachleuten diskutiert und dann aber natürlich die konkrete Übertragung, damit sollten sich auch die Kommunen beschäftigen, das finde ich schon sinnvoll.
26	**LT:** Und sehen Sie Auswirkungen bei der Reformierung der Gewaltenverteilung in Baden-

	Württemberg? Also, dass jetzt die Staatswälder von der Anstalt des öffentlichen Rechts ForstBW übernommen werden seit 2020 und der Körperschaftswald in Karlsruhe nur noch vom Forstamt?
27	**A2:** Also direkte Auswirkungen jetzt auf die Wälder, kann ich noch nicht feststellen. Es ist natürlich, sage ich jetzt mal, eine gewisse Befürchtung da, dass ein Forstbetreib vielleicht die wirtschaftlichen Aspekte auch stärker gewichtet, aber das wird die Zukunft zeigen, wie sich das auswirkt.
28	**LT:** Und für die politischen Handlungen sehen Sie auch noch keine Auswirkungen?
29	**A2:** Für die politischen Handlungen.
30	**LT:** Oder jetzt auch in Ihrer Arbeit?
31	**A2:** Natürlich ist es komplexer geworden, weil man einfach bei der Forstverwaltung mehr unterschiedliche Player hat. Also es ist ja jetzt eben auch so, dass im Staatswald jetzt nicht nur ForstBW zuständig ist, sondern es ist natürlich nach wie vor auch die Landesforstverwaltung für bestimmte Dinge zuständig, d.h. ich habe hier einfach eine Vervielfachung der zuständigen Personen für einzelne Teilbereiche und das macht es für uns auch etwas komplexer.
32	**LT:** Dann würde ich zum Abschluss noch fragen. ob Ihnen in dem Zusammenhang zum ganzen Thema noch was einfällt, oder ob Sie noch was loswerden wollten?
33	**A2:** Also wie gesagt, der Hauptpunkt, der mich eigentlich stört, ist diese Fokussierung auf rasche Widerbegründung der Waldflächen, insbesondere eben mit nicht-autochthonen Baumarten und wir haben eben sehr, sehr viele Tierarten, die sind angepasst an unsere ehemaligen Baumarten und ich glaube, man geht hier ein bisschen vorschnell weg von den ehemaligen heimischen Baumarten, ohne wirklich auch zu testen, ob nicht z.B. durch Naturverjüngung, durch eine breite genetische Basis der heimischen Baumarten, vielleicht eben auch diese weiterhin dem Klimawandel standhalten können. Es gibt z.B. auch deutlich weiter südlich Buchenwälder in weiter südlichen Gegenden und da finde ich geht man wirklich etwas vorschnell auf andere Baumarten, wie Roteiche oder ähnliches. Das finde ich sehr kritisch, weil eben die speziell angepassten Arten damit dann natrülich auch Probleme bekommen.
34	**LT:** Und wäre dann eine Ausweitung der Naturschutzgebiete auch eine Lösung oder striktere Regelungen?
35	**A2:** Ja das sind eher eigentlich jetzt die Konzepte zum Umgang mit dem Klimawandel. Also, wie stark setze ich jetzt eben auf andere Baumarten? Eine Ausweisung zum Naturschutzgebiet ist nicht die Lösung. Also, weil wenn Sie sich mal die Naturschutzgebiete anschauen, dann ist in den meisten Naturschutzgebieten steht da drin, dass die ordnungsgemäße Forstwirtschaft zulässig ist. D.h. es müsste eigentlich dann auch in der Naturschutzgebietsverordnung spezielle Regelungen dazu geben. Das ist bislang in den wenigsten Naturschutzgebieten gelungen, also von daher ist, wäre nicht eine pauschale Ausweisung als Naturschutzgebiet wichtig, sondern allenfalls eine Ausweisung mit speziellen Regelungen bezüglich dann der Forstwirtschaft.

A3

1	**A3 (40:21.2 Minuten)**
2	[Dialog von 00:00.0-00:48.8 nicht transkribiert, um Anonymität zu wahren]
3	**LT:** Was bedeutet für Sie das naturnahe Waldmanagement?
4	**A3:** Das ist eine sehr komplexe Frage. Ich sage mal, ich persönlich verstehe den Wald auch als Ökosystem. Und es wurden natürlich für den Stadtwald auch ganz spezielle Ziele definiert, zu denen auch die naturnahe Waldbewirtschaftung gehört. Ich arbeite jetzt schon seit über 30 Jahren hier und habe letztendlich ein Stück weit diese drei Jahrzehnte Umstellung auf naturnahe Waldwirtschaft miterlebt und mitgestaltet. Und diese naturnahe Waldbewirtschaftung hat natürlich ganz viele Komponenten, die eben als zentralen Baustein haben auch möglichst viele natürliche Abläufe zu integrieren. Wie z.B. die natürliche Waldverjüngung, aber es sind natürlich

	da was Naturnähe der Waldbewirtschaftung heißt auch ganz unterschiedliche Vorstellungen da. Die Einen sehen unter Naturnähe z.B. die Maximierung der Biodiversität. Andere sehen wiederum spezielle Leitfaden im Fokus. Wir haben gerade eine aktuelle Diskussionen, jetzt auch letztens in einem Ausschuss gehabt, da geht es um die lichten Wälder. Naturnahe Waldwirtschaft ist ja eher eine dunkle Waldwirtschaft. Kleinflächig, Naturverjüngung ausnutzen, keine Kahlschläge, wenn möglich keine Kahlschläge. Insofern leiden da schon etwas die Lichtwaldarten und jetzt kommen zum Teil wieder Forderungen eben aus Gründen der Erhöhung der Biodiversität bestimmter Arten mehr Licht in den Wald zu bringen über Kahlschläge z.B., die wieder größer werden. Also es gibt da ganz unterschiedliche Vorstellungen, ob es nun zentrale Elemente sind. Elemente wie eben Ausnutzung von Naturverjüngung, überwiegend auch natürlich die Großdiskussion, welche Baumarten verwendet man? Also meine Mitarbeiter*innen und ich halten nach wie vor sehr stark an den standartheimischen Baumarten fest. Aus meiner Sicht werden wir aber auch einen bestimmten Anteil nicht-heimischer Baumarten brauchen, die bewährt sind im Anbau. Es wurde dazu auch letztes Jahr ein Anpassungskonzept des Stadtwaldes an den Klimawandel erarbeitet und beschlossen im Gemeinderad, ich weiß nicht, ob Sie das schon gesehen haben. Es steht ja auch auf der Homepage drauf und ist öffentlich zugänglich. Dort sind auch die Ziele definiert. Es sind natürlich beim Stadtwald auch als übergeordnete Ziele nicht die Optimierung der Holzproduktion drin, sondern die Ziele Ökologie und Erholung, Freizeit, Soziales ist natürlich der übergeordnete Zielsetzungsbereich im Stadtwald.
5	**LT:** Genau über dieses Konzept der Klimaanpassung wollte ich auch reden. Wie bewerten Sie denn da die Anwendung zurzeit für den Stadtwald in Karlsruhe?
6	**A3:** Gut sagen wir mal, dieses Konzept umfasst jetzt keine ganz wesentlich neuen Ideen, sondern setzt aus meiner Sicht neue Schwerpunkte. Einerseits was die Pflege vorhandener Waldbestände angeht. Dort geht es eben sehr stark darum, dass man die Kronenentwicklung von Bäumen fördert, um möglichst stabile Einzelbäume zu bekommen und dann zweiter Schwerpunkt, wie geht man eben mit geschädigten Waldbeständen um? Setzten auf Naturverjüngung, Ergänzungspflanzungen für konkurrenzschwache Baumarten und Baumarten, die sind da natürlich verjüngt schwer haben, das ist ein ganz zentrales Element natürlich, also Baumart Eiche. Man ist da eigentlich in den letzten Jahren schon auf diesem Weg, dass man Naturverjüngung kombiniert. Man wird jetzt ein Stück weit überholt eben durch diese Waldschaden Situation. Gerade jetzt speziell Eschentriebsterben ist ein ganz großes Problem, weil da Flächen junger Bestände ausfallen. Und dann auch die natürliche Verjüngung im Prinzip in den Hintergrund drängt, weil dann eben keine Samenbäume auf diesen Flächen stehen. Gibt da natürlich auch über Samenflug Eintrag, aber dann gibt es sehr stark den Vorhang für z.B. den Bergahorn. Die Eiche kommt auf diese Flächen nicht von alleine und da ist man natürlich auch immer in der Diskussion drin, ist der Bergahorn da auch langfristig stabil? Lässt man den einfach laufen? Was natürlich bestimmte Vorteile hat. Z.B. dass man nicht Pflanzen muss mit großem Aufwand, aber man hat natürlich auch bei Bergahorn, dass man durch den Rußrindenpilz massive Ausfälle hat. Dass der Bergahorn auch als Baumart nicht stabil in die Zukunft führen wird. Insofern setzt man eben auch auf Baumartenvielfalt. Bei der Naturverjüngung gucken was kommt. Für das Amt wichtig eben ist eine kleinflächige Betrachtung der Wälder. Das Amt hat auch immer festgehalten an Revieren, die von der Fläche her überschaubar bleiben. Damit die Revierförster, wenn sie ihren Wald auch sehr gut kennen, das ist für mich die Voraussetzung auch einer naturnahen Waldbewirtschaftung. Ich spreche manchmal schon davon, dass das was wir jetzt auch durch diese Waldschäden bedingt betreiben ja so ein bisschen in Richtung Waldgärtnerei geht. Sehr kleinflächig, sehr angepasst an die jeweilige Verjüngungssituation, aber ich glaube gerade im stadtnahen Wald ist das eine wichtige Voraussetzung, um eben diesen Funktionen, die gefordert sind gerecht zu werden.
7	**LT:** Und welche politischen Konflikte sehen Sie bei der Anwendung der Klimaanpassung?
8	**A3:** Gut sagen wir mal, wir haben ja diese Diskussionsfelder gehabt. Man hat da dieses Klimaanpassungskonzept nicht im stillen Kämmerlein entwickelt, sondern hat da drei Runden gedreht auch mit Verbänden zusammen. Das ist mir auch immer wichtig. Bei der letzten Forsteinrichtung wurde das schon so praktiziert, dass man im Vorfeld im Workshop mit

	einbezogen hat. In die letztendliche Zieldiskussion dann im Gemeinderat. Und die zentralen Themen waren eben die Frage: Einbringung nicht-standartheimischer Baumarten; die Frage: Pflanzung oder Naturverjüngung und auch zuletzt ein Stück weit die Frage des Holzeinschlages, des Ausmaßes des Holzeinschlages in der Folge zu den Waldschäden. Und ich denke, es wurden da auch natürlich gewisse Kompromisse schließen müssen, geschlossen. Bei der Frage der Beteiligung nicht-standartheimischer Baumarten gab es natürlich auch diejenigen, die gesagt haben, der Wald der Zukunft soll komplett mit den heimischen Baumarten aufgebaut sein. Keine Verwendung von nicht-heimischen Baumarten. Ging es dann natürlich auch um Diskussion, gibt es vielleicht Herkünfte, von den heimischen Baumarten, die den Trockenstress, die erhöhten Temperaturen vielleicht besser ertragen? Und meine fachliche Meinung ist die, dass wir natürlich ein Grundaufbau beibehalten sollen nach Möglichkeit so lange das geht aus heimischen Baumarten, dass wir aber auch einen gewissen Anteil von nicht-heimischen Baumarten brauchen, die bewährt sind bei uns. Das sind ganz klar die Roteiche, die Douglasie. Und man hat im Konzept auch ganz speziell die Invasitätsproblematik abgearbeitet, weil da muss man natürlich aufpassen, dass man keine Baumarten einbringt, hat man ja teilweise auch schon, die dann durch die Invasivität das Gleichgewicht mittel- und langfristig stören und in Gefahr bringen. Das ist ein ganz spezielles Themenfeld sicher. Aber gerade auf schwierigen Standorten, Sie kennen das ja auch, extrem trockene, sandige Böden, da bin ich der Meinung, dass wir da einen gewissen Anteil an nicht-heimischen Baumarten brauchen. Da denke ich speziell an die Roteiche. Das ist eigentlich momentan die einzige Baumart, die bisher keine Trockenschäden zeigt und vital geblieben ist. Um, sagen wir mal, für den Erholungsbereich aus meiner Sicht ganz zentral ist auch die Sache des Kleinklimas im Wald. Die Menschen aus der Stadt gehen ja gerne in den kühlen Wald. Temperaturen sind da gleich 5,6, Grad niedriger, als im Stadtbereich. Und um diesen kühlen Wald aber auch zu bekommen, brauche ich letztendlich ein geschlossenes Kronendach, ich brauch die Schattenwirkung der Bäume. D.h. vielleicht aus Biodiversitätsgründen eher lichter Wald, der ist eher ein lichter Waldtypus bringt dann wieder Nachteile, weil dann dieses Waldinnenklima nicht mehr vorhanden ist. Und für mich ist das ein ganz wichtiger Aspekt, den Menschen auch in Zukunft nach Möglichkeit diesen geschlossenen Wald mit einem angenehmen Waldinnenklima noch bereitstellen zu können. D.h. da und dort sicherlich auch lichtere Strukturen, die wir automatisch bekommen durch die Waldschadenssituation aber auch versuchen eben auf schwierigen Standorten ein geschlossenes Kronendach zu bekommen. Weil gerade diese schwierigen Standorten Hardtwald und so, die sind ja die Hotspots auch im urbanen Bereich dort braucht der Menschen bei den Temperaturen auch den Ausgleich. Also unser Ziel bei unserer Arbeit ist eigentlich der Ausgleich der vielen Interessen, die an den Wald gestellt werden und da gibt es letztendlich immer dann diese Konflikte eben, dass man es einzelnen Interessengruppen, mit ihren speziellen Interessen vielleicht nicht so hundertprozentig richtig machen kann und insofern wird das nie eine hundertprozentige Zufriedenheit in allen Gruppen geben. Gerade auch im Erholungsbereich haben wir Diskussionen jetzt Mountainbike, Radfahren, Dirtbikestrecken im Wald, da gibt es ganz, ganz viele Konfliktfelder, die sich auftuen, wo man einfach, ja aber auch gewisse Barrieren hat. Wald ist eben ein gesamtkomplexes System und nicht das Spielfeld von einzelnen Interessen.
9	**LT:** Und welche politischen Instrumente fehlen dann da noch als Lösungsansätze für die Zukunft?
10	**A3:** Also ich glaube, wir haben da ganz gut politische Instrumente bisher entwickelt. Aber ein ganz wichtiger Punkt für mich ist die Beteiligung. Beteiligungskonzepte zu entwickeln, nicht so im stillen Kämmerlein zu agieren. Die Mitarbeiter*innen des Amts sind sicherlich auch nicht die einzigen, die eine Ahnung haben, da gibt es noch ganz viele andere Leute, Fachleute, die in ihren Fachbereichen Spezialkenntnisse haben, die ich und meine Kolleg*innen nicht haben. Die gilt es auszutauschen, die gilt es auch einzuarbeiten. Konzepte zu entwickeln, das ist ein permanenter Prozess und dieser Austausch ist wichtig. Knackpunkt von solchen Beteiligung und Verfahren, sage ich jetzt mal ganz allgemein, ist, dass es zeitaufwendig ist. Dass es notwendige Abstimmungen braucht und diese Zeit ist manchmal einfach sehr knapp. Aber ich glaube, um eine intensive Beteiligung kommt man nicht rum. Und, wie gesagt, man hat das schon bei der letzten

	Forsteinrichtung praktiziert, 2016/17 im Workshop. Man hat es jetzt beim Waldanpassungskonzept gemacht, man macht sehr viele Waldbegehungen bis hin zu der Waldpädagogik. Mit einem Angebot für alle Zielgruppen, Erwachsene, Familien, etc. Das Amt ist in den politischen Gremien präsent. Gerade jetzt in diesen Diskussionen vom Klimaanpassungskonzept oder gerade jetzt letztens auch mit im Ausschuss. Man hat ja einen Singletrail eingerichtet, da gab es gestern einen Erfahrungsbericht nach einem Jahr, wo der jetzt besteht. Oder auch jetzt in der Diskussion um die Mitgliedschaft im Naturpark, der Berg[14:36.3?] etc. etc. Da gibt es ganz viele Instrumentarien, die das Amt bespielt und bespielen muss. Wichtig ist mir auch immer ein Austausch mit den Verbänden. Ich meine, wir haben hier in Karlsruhe Verbände, die sehr aktiv sind und die wirklich auch, würde ich sagen, sehr fachkundig sind was einen Wald angeht. Und man hat jetzt gerade wieder so eine Diskussion gehabt um das Thema Bewässerung von Forstkulturen, von Pflanzungen. Wo auch große Diskussionen entstanden sind und Schreiben geschickt worden sind dazu. Und man hat letztens dann eine Video-Runde gemacht mit den Umweltverbänden, um dann vielleicht Missverständnisse, die aufgekommen sind, auszuräumen. Das ist ganz klar und da geht man auch auf Bedenken ein. Da geht es ja speziell darum, ob das Amt in Trockenzeiten Bewässern, aus Oberflächengewässern Wasser entnehmen kann, um eben die frisch gepflanzten Bäumchen gießen zu können, um einen Objektschutz betreiben zu können. Also nicht als Regelmaßnahme, sondern als reine Notmaßnahme. Man hat jetzt daraufhin den Antrag auf die Entnahme aus Oberflächengewässern zurückgezogen. Das erhöht zwar massiv den Aufwand und die Kosten aber, das beseitigt dann die Bedenken der Verbände, dass dann bei Trockenzeiten vielleicht dann Schäden an Gewässern entstehen könnten. Also solche Dinge muss man einfach miteinander besprechen. Wie gesagt, extrem zeitaufwendig. Das Amt wird manchmal etwas belächelt, da es in Karlsruhe ja jetzt nur noch für den Stadtwald betrieblich zuständig ist mit einer relativ kleinen Fläche von 2.300 ha, aber es gibt so wahnsinnig viele Fragestellungen. Und es gibt so viele Bedürfnisse auch diese Fragen untereinander abzustimmen. Und dieser Aufwand ist enorm groß, den darf man auch nicht unterschätzen, aber wenn wir den nicht betreiben, dann glaube ich, werden wir auf wenig Akzeptanz unserer Maßnahmen stoßen. Wichtig ist dem Amt auch, abschließend auch noch vor Ort noch stärker zu informieren im Wald mit Informationsschildern, mit angebotenen Waldführungen. Das Amt macht auch, wenn irgendwelche Bürger auf es zukommt, auch Einzelpersonen, die irgendwelche kritischen Anmerkungen haben, also Fragen haben, bieten Mitarbeiter*innen gleich an, komm, geh mal mit raus, ich zeige dir das, erläutern das, wie wir das sehen, im [17:26.8?] wie du das siehst". Und insofern versucht man da auch eine Nähe zum Bürger zu haben, die einfach auch wichtig ist. Und als kommunales Amt ist man ja ganz nah auch an den politischen Entscheidungsträgern dran, die kommen dann auf das Amt zu, wenn sie irgendwelche Fragen haben und man hat jetzt auch wieder eingeführt, dass man regelmäßig mit dem Ausschuss und auch mit den Naturschutzvereinen in den Wald geht. Das Amt geht mit den Ortschaftsräten in den Wald. Und das ist ganz, ganz wichtig.
11	**LT:** Jetzt haben Sie es eigentlich schon recht viel angesprochen, aber mein zweites Thema ist die Erholungsfunktion, die jetzt gerade in Zeiten der Covid-19-Pandemie viel mehr nachgefragt wurde, also die Stadtbevölkerung sucht den Wald verstärkter auf und ein paar Konflikte hatten Sie auch schon genannt. Haben Sie den Eindruck, dass die Konflikte seit dem Ausbruch der Pandemie auch verstärkt sind oder sind noch neue dazu gekommen?
12	**A3:** Ja. Also ich sage mal, was auf jeden Fall für uns deutlich wird, dass der Wald und natürlich auch die sonstigen Freiräume unheimlich stark frequentiert werden. Auch von Gruppierungen, Gruppen, die bisher, ich sage jetzt mal, eher ins Sportstudio gegangen sind in der Freizeit. Es ist auch so, dass Menschen, habe ich den Eindruck, auch nicht so nur die gewohnten Wege gehen, die sie vielleicht leichter erreichen, weil dort so viel los ist, die erkunden dann auch diese ruhigeren Ecken. Das Amt wird auch gezielt immer wieder angefragt: "Na, wo gibt es denn mal eine ruhigere Ecke, wo ich vielleicht auch mal hinkann?" Aber das ist natürlich auch sehr sensibel, dann noch diese ruhigen Ecken zu empfehlen, die wir mittlerweile sehr selten nur noch haben. Und insgesamt beobachte ich schon, die Mitarbeiter*innen draußen und meine Kolleg*innen, die in

	der Fläche arbeiten, dass die Individualinteressen doch sehr stark in den Vordergrund rücken. Und was, glaube ich, ganz zentral ist als Botschaft ist das Thema Respekt vor der Natur auch haben ein Stück weit. Und, sagen wir mal, auch einsehen, dass die Natur auch ein Recht hat. Und was in den letzten Jahren nicht unbedingt Corona bedingt ist, das war auch vorher schon so, hat sich aber natürlich auch verstärkt, sind z.B. auch Aktivitäten in der Nacht. Es gibt da jetzt überall in dem Supermarkt diese extrem starken Stirnlampen zu kaufen. Die nutzen die Jogger, die nutzen Spaziergänger, die nutzen Radfahrer, um dann querbeet durch den Wald zu fahren. Und das ist eine unserer Botschaften, dass wir da zumindest in der Nacht auch der Natur ein Stück weit Ruhe gönnt. Ich bin jemand, der sagt, weiter Regelungen, über Verordnungen und Gesetzte, ja das kann man zwar machen, Leinenzwang per Gesetzt verordnen und solche Dinge, aber dann brauche ich auch wieder jemanden, der das kontrolliert, dann komme ich später mit Verboten und Bußgeldern. Ich glaube, damit kann man vielleicht ein bisschen was bewirken, aber letztendlich geht es um die Einstellung der Menschen. Es gibt viele Menschen, die sich heute um den Naturschutz, Umweltschutz engagieren, aber wenn sie dann mit ihrem Mountainbike unterwegs sind, vergessen sie das alles und fahren querbeet durch den Wald und vergessen die guten Vorsätze, weil es einfach Spaß macht. Und da gilt es auch einfach wahnsinnig viel zu informieren, Öffentlichkeitsarbeit zu machen. Aber auch gerade beim Mountainbiken, haben wir auch gesagt, man kann nicht immer nur verbieten und "nein" sagen. "Hier dürft ihr nicht durch, dann da dürft ihr nicht". Deswegen hat das Amt mit dem Mountainbikeclub zusammen auch in einer weiten Abstimmungsrunde, das hat fast zwei Jahre gedauert, einen Trail, diesen Singletrail in einer Ortschaft zu legalisieren. Das hat gut funktioniert und hat auch Erfolg gebracht, das hat auch aktiv illegale Strecken, die sensibel sind von der Ökologie her zurückgebaut. Aber solchen Strecken ziehen natürlich auch wieder andere Nutzer von weiter her an. Diese Communities, ob das jetzt die Läufer sind, die Radfahrer oder die Mountainbiker oder die Schatzsucher da. Jeder hat heutzutage eine eigene Community, die gut vernetzt ist. Die sagen dann, man, da ist was Tolles und geh da mal hin und geh dort mal hin. Und da kommen dann trotzdem der Schwerpunkt bei der örtlichen Bevölkerung liegt, dann doch eben die Menschen mit dem Auto angefahren aus angrenzenden Städten, Landkreisen, aus weiter entfernten Bereichen, um dann diese Dinge auszuprobieren, wie jetzt diese Mountainbiketrails. Es kommt eben auch dazu, dass die Menschen eben sehr viel Freizeit haben auch tagsüber im Homeoffice freie Zeit der Arbeitsgestaltung, da geht man tagsüber dann doch mal mehr raus und arbeitet dann abends noch. Hat ja Vorteile, aber bringt eben den Druck. Und jetzt haben wir was sehr stark zugenommen hat ist auch das Thema Konflikte zwischen Spaziergängern, Radfahren und freilaufenden Hunden. Das ist so ein Thema, das momentan bewegt, was immer wieder Einforderungen gibt nach Einführung von Leinenzwang. Wir haben ja momentan keinen. Nur in Schutzgebieten z.B. Aber wie gesagt, ob das der richtige Weg ist? Ich weiß es nicht so richtig. Was wir auch feststellen, ist zunehmend eben Vermüllung. Vor allen Dingen so an ein paar Plätzen, wo Sitzbänke stehen, wo Hütten sind, wo sich eben Jugendliche, wo der Club zu ist, wo ihr Treffen im privaten Umfeld nicht mehr möglich ist, nicht mehr erlaubt ist, die treffen sich dann nachts irgendwo im Wald. Und am nächsten Morgen stellt man fest: überall liegen die Bierflaschen ganz oder zerdeppert, irgendwelche Schilder sind um getreten dann oder auch weitere Schäden an Hütten z.B. Jetzt letztes Wochenende gab es wohl auf dem Downhilltrail an einer Gemarkung Beschädigungen von Schildern und sonstigen Einrichtungen. Ja, da kann kommt es einfach zu Vandalismus oder an den Grillstellen auch. Und das sind unliebsame Erscheinungen, die jetzt auch Corona bedingt sind, wenn die Gruppierungen, gerade Jugendliche, auch wieder mal daheim feiern können oder unauffällig sich treffen können, dann hört das vielleicht auch wieder ein bisschen mehr auf. Aber da müsste man eigentlich viel mehr unterwegs sein, auch nachts. Da passiert natürlich auch viel. Aber das können wir uns einfach nicht leisten.
13	**LT:** Jetzt sind ja der Klimawandel und die Covid-19-Pandemie sehr globale Problematiken. Finden Sie, dass sie sich deswegen am besten auf globalster oder bundesweiter Ebene lösen lassen oder ist das auf lokaler Ebene besser anzugehen?
14	**A3:** Ich glaube, das kann man nicht voneinander trennen. Man braucht natürlich diese

	grundlegenden Entscheidungen. Gerade was den Klimaschutz angeht. Wenn diese Dinge, die wir jetzt im Wald hier machen, Waldanpassung etc. Das sind eigentlich nur Hilfskrücken. Beim Klima muss letztendlich durch Klimaschutz wirkungsvoll reagiert werden, um dieses 1,5-Grad-Ziel z.B. einzuhalten. Also das sind natürlich ganz wichtige Dinge, die zur Walderhaltung grundsätzlich den Grundstein legen müssen. Aber ich sage mal, die lokalen Maßnahmen, die Anpassungsmaßnahmen, die müssen natürlich auch ein Stück weit vielleicht Zeit überbrücken. Ich kann jetzt aber nicht die Hände in den Schoß legen und sagen: "Jetzt warte ich mal bis sich da irgendwas auf globaler oder bundesweiter Ebene tut und so lange mache ich da nichts." Sondern man muss das irgendwie miteinander verlinken. Aber Klimaanpassung ohne Klimaschutz ist eigentlich unsinnig. Der Klimaschutz mit zentralen politischen Maßnahmen, der muss ganz im Vordergrund stehen. Und ich glaube das muss allen klar sein, die sich auch für den Walderhalt einsetzten. Also ich spreche deshalb auch ungern vom Waldumbau, das erweckt immer so den Eindruck, jetzt wir wollen den jetzigen Wald -ich sage es jetzt mal ein bisschen flapsig-plattmachen. Und irgendwas neues Pflanzen im Wald der Zukunft, der dann Mittelmeercharakter hat und der diese 2,5 oder 3,5 Szenarien dann vielleicht auch noch aushält. Sondern es geht einfach jetzt darum jetzt auch noch diese bekannten Waldgesellschaften, diese Ökosysteme zu erhalten, zu strecken und in der Hoffnung, dass eben auch diese Klimaschutzmaßnahmen greifen und wir tatsächlich auch Szenarien oder ein Szenario haben, wo diese Waldökosysteme auch noch eine Zukunft haben und nicht völlig umgestellt werden. Und bei dem Thema Covid-19 ist es das gleich. Wobei das kann man natürlich sehr kontrovers diskutieren. Ich denke mal, auch ein Teil vom Wald stärkt letztendlich auch das Immunsystem, stärkt auch die Abwehrkräfte und sportliche Betätigung ja genauso. Insoweit ist es sicherlich von Vorteil, wenn die Menschen sich draußen aufhalten können. Und es gilt da sich an gewisse Regeln zu halten und die sind auch relativ einfach. Wir sind auch gerade daran zu einer, aber das ist immer so eine Frage der Ressourcen. So eine Art Wald-Knigge wieder zu beleben. Das gibt es ja auch. Z.B. über das Zertifizierungssystem PEFC gibt es solche Hinweise. Bleibt auf den Wegen; Leint eure Hunde an, zumindest jetzt in der Setz- und Brutzeit. Und bleibt nachts aus dem Wald raus, gönne sie der Natur die Ruhe. So ein paar ganz wenige Grundregeln. Dann passt das eigentlich auch.
15	**LT:** Und was ist Ihre Meinung zu den Auswirkungen des Holzkartellverfahrens? Also dass jetzt der Staatswald von der Anstalt des öffentlichen Rechts ForstBW übernommen wurde?
16	**A3:** Also, ich sage mal, da hat das Amt sich immer auch dagegen ausgesprochen. Auch im Vorfeld. Die Stadt und auch der Gemeinderat haben sich sehr stark gemacht. Bürgervereine haben sich dafür sehr stark gemacht, auch schriftlich. Wir wollten ja eine andere Lösung für das Stadtgebiet von Karlsruhe. Weil hier der Staatswald ja mit der gleichen Größe wie der Stadtwald natürlich eine sehr enge Beziehung zu der Stadtbevölkerung hat. So ein großer Block der Hardtwald nördlich von Schloss, was Sie vielleicht auch kennen. Ganz zentraler Erholungsraum der Karlsruher. Und wenn Sie sehen im Süden gibt es die Kernstadt, dann links Nordstadt, [29:17.8?], im Osten die Waldstadt. Also ganz bevölkerungsreiche Naherholungsgebiete. Und ich bin nach wie vor der Meinung, dass die jetzige Struktur mehr Nachteile als Vorteile bringt. Aus der Sicht der der Bürgerschaft vor allen Dingen. Man hat immer wieder Anfragen, wer ist denn jetzt für was zuständig? Da beschweren sich die Bürger über den Zustand der Waldweges oder über den Zustand des Trimmdich-Pfads und dem Bürger gegenüber ist es immer die schlechteste Wahl, wenn ich sagen muss: "Ich bin nicht zuständig, geh da und dort hin." Das begreifen man nicht. Und diese Strukturen, die wir jetzt im Forst haben, mit der Doppelzuständigkeit: einerseits hoheitliche Aufgaben, andererseits Betrieb. Das ist natürlich auch ganz, ganz schwer zu vermitteln und das bringt Doppelzuständigkeiten und das ist aus Bürgersicht oder z.B. auch aus Sicht der Bürgervereine auch immer ganz schwierig. Und insofern, sind meine Kolleg*innen und ich nach wie vor der Meinung, für den Stadtbereich, für die Bevölkerung hier, auch für die städtischen Dienste und Ämtern, die wissen auch sehr oft nicht, was ist das denn jetzt für ein Wald? Ist das städtischer Wald, ist das Staatswald? Auch die Zuständigkeiten, ich meine, weit weg. Man hat da auch im Staatswald auf zwei Betriebteile. Ein Betreibsteil sitzt in der Zentrale in Kirrlach, das ist 40km von Karlsruhe weg. Und das andere in Eppingen, auch so weit weg. Und die Kollegen, bei

	allem Bemühen, der Kollegen dort, wir haben da auch in der Weite gut kollegiale Kontakte, aber der Kollege, der in Kirrlach sitzt, bekommt auch einfach politisch nicht mit, was hier in Karlsruhe auch gerade läuft. Wo der Schuh drückt, wo Probleme auftauchen, wo Interessenkonflikte vielleicht auch da sind. Und das ist für die viel schwieriger das aus der Ferne dann zu managen. Also insofern, keine gute Situation aus Sicht der Stadt. Ich bin ein Verfechter, das sage ich auch ganz offen, urbane, stadtnahe Waldbewirtschaftung ist in der Kommunalstruktur sicherlich sehr gut aufgehoben, weil man da einfach diese Netzwerke hat, die man braucht, als, ich sage jetzt mal: "Manager des Waldes, als jemand, der eben nicht nur die Holzproduktion oder jagdliche Dinge im Kopf hat, sondern viele, viele andere Dinge." Und die Strukturveränderung war politisch so gewollt, man muss sie so umsetzten, versucht auch nach Möglichkeiten über die Kommunikation miteinander, was aber auch nicht immer ganz einfach ist, weil jeder seine Schwerpunkte hat, seine Strukturen und seine Orientierungspunkte hat. Wir sind in die Stadtstruktur eingebunden, die Kollegen eben in diese Staatswaldstrukturen. Und das sind jetzt mittlerweile unterschiedliche Welten ein Stück weit geworden. Insofern nicht so ganz einfach.
17	**LT:** Gibt es zum Abschluss noch etwas Wichtiges, was Sie im Zusammenhang vom Interview noch los werden wollen?
18	**A3:** Ich glaube wir haben jetzt ein breites Potpourri durchgemacht. Dann kann man überlegen. Einmal was man, bei allem wir haben sehr stark diesen Schwerpunkt Ökologie, Erholung, aber ich bin auch immer ein Vertreter, auch nach wie vor, der Holznutzung im Wald. Das gehört auch zu einer umfassenden Nachhaltigkeit dazu. Wir leben eben von Rohstoffen aus der Natur, die die Natur uns produziert. Und insofern glaube ich auch, dass wir es den künftigen Generationen ein Stück weit schulden, dass wir auch ihnen Nutzungsoptionen in der Zukunft eröffnen, weil wir wissen nicht, was für Ansprüche die Gesellschaft in 20, in 40 oder in 100 Jahren hat. Wir können es uns heute leisten, sage ich ganz bewusst, dass wir diese ökologische und Erholungsfunktion in den Vordergrund stellen und die Nutzung des Waldes in den Hintergrund schieben, ob das immer so bleibt, das ist eine gesellschaftliche Frage, Aber so ganz sollte man den Rohstoff Holz, der ja auch ökologisch viele, viele Vorzüge hat nicht aus den Augen verlieren. Insoweit gehört auch die Pflege des Waldes mit dem Ziel auch auf der einen Seite vitale und stabile Bäume zu bekommen, die aber auch mal eine gute Holzqualität liefern und nicht nur Brennholz, das gehört sicherlich dazu. Das ist ja auch so ein bisschen die Vorstellung von verschiedenen Waldbaumodellen, die da immer wieder propagiert werden, dass man eben nicht auf Masse setzt, sondern eben auf Qualität setzt. Also diesen Fokus sollte man nicht ganz ausblenden. Deshalb bin ich auch ein Gegner, das kommt ja auch momentan immer wieder sehr stark, lass doch den Wald sich selber machen. Ich sage jetzt mal Stichwort: diese Vorstellung eines bekannten Försters und Autors: der Wald reguliert das alles selber und der Wald macht das alle viel besser als die Forstleute. Das ist, glaube ich, nicht das Mittel der Wahl, dieser sogenannte Prozessschutz. Und es ist auch sehr stark schwarz-weiß gemalt. Und Schwarz-Weiß-Lösungen sind nie gut. Vor allen Dingen im Wald und in der Natur nicht. Für mich ist das so, dass der Prozessschutz, also dieses der Natur sich selbst überlassen, sicherlich ein wichtiger Teil in einem Gesamtwaldschutzkonzept ist von Einzelbäumen, die stehen bleiben, bis hin zu Großschutzgebieten, wie Nationalpark. Aber als Lösung, Prozessschutz, gerade in urbanen Wäldern, wo wir auch noch die Problematik mit den Neophyten haben, Kermesbeere, [35:45.7?], wie sie alle heißen, Robinie, Götterbaum, was sich noch stärker verbreitet. Da konkurrenzschwache Baumarten wie die Eiche z.B., da braucht es aus meiner Sicht eines aktiven Pflegemanagements, das eben aber auch beinhaltet entsprechende Waldnaturschutzkonzepte von biodiversitätssteigende Maßnahmen für Lichtwaldarten, waldbrandfähige z.B. Bis hin auch bis zu Prozessschutzflächen. Wir haben das bei uns auch im Stadtwald. Gott sein Dank eine Mitarbeiterin, die sich speziell um das Thema Waldökologie im Naturschutz kümmert. Da gibt es auch viele Spannungen und Diskussionen, gerade um Prozessschutzflächen. Aber jetzt so Prozessschutz auch um jeden Preis, wäre in diesen urbanen Wäldern, aus meiner Sicht, der falsche Weg. Und ich glaube aber auch, dass da viele Naturschutzverbände der gleichen Ansicht sind. Was klar ist, wenn man den Wald sich selber überlassen würde, der Wald verschwindet nicht. Es wird immer Wald geben. Die Frage ist nur,

	wie sehen diese Wälder aus? Kurzfristig, mittelfristig und langfristig? Gibt es auch sicherlich differenzierte Ansichten dazu, das ist auch sicherlich richtig. Aber wir brauchen, ich sage mal, wenn ich den Leuten in einem Karlsruher Bezirk sage: "Wir lassen jetzt einfach euren Wald so liegen, wie es ist. In 500 Jahren ist da dann wieder ein toller Buchenwald.", dann sagen die: "Spinnt der? Ist der jetzt noch ganz wach? Was in 500 Jahren ist interessiert mich überhaupt nicht, sondern mich interessiert was hier in 5 Jahren, im nächsten Jahr oder in 10 Jahren ist." Also die wollen eher eine kurzfristige Perspektive haben. Also das ist immer so eine Betrachtungsweise und ich bin vollkommen gegen diese Schwarz-Weiß-Bilder und diese mittlerweile haben sich da schon ein paar feindselige Gegenpositionen entwickelt. Und also, wenn der bekannte Förster und Autor zum Tag des Baumes, z.B., ein Post sendet: "Beteiligt euch nicht an Pflanzaktionen von öffentlichen Forstverwaltungen, solange noch Buchen und Eichen gefällt werden!", dann ist das Zeug, das sowas von Ideologie und anscheinend wenig Fachwissen, wobei das glaube ich nicht, also ich unterstelle dem bekannten Förster und Autor, dass der ganz viel Fachwissen hat. Aber ich glaube, da gibt es ganz viel dazwischen und da müssen wir, um den richtigen Weg immer diskutieren, kämpfen. Nachhaltigkeit ist schon ein Diskussionsprozess. Aber auch, wir haben jetzt so ein Beispiel, da kann man sich natürlich auch drüber unterhalten: Verkehrssicherung ist momentan ganz heißes Thema. Man versucht bei der Verkehrssicherung auch baumerhaltend vorzugehen. Sprich nicht jeden Baum gleich zu fällen, sondern auch Kronenrückschnitte zu machen oder wenn da eine Bank unter einem brüchigen Baum steht, dann wird halt die Bank versetzt und an anderer Stelle aufgebaut. Wir haben jetzt auch z.B. zum ersten Mal einen Weg, einen Fußweg, ich weiß nicht, ob Sie es kennen, bei einer Wohnsiedlung, dauerhaft gesperrt. Weil dort ganz viele alte Buchen im Waldrandbereich stehen, die massive Dürreschäden haben und dort kommt man mit dem Hubsteiger nicht hin, da müsste ich, wenn ich den Weg offenlasse, müsste ich eigentlich sagen, da müssen die ganzen alten Bäume gefällt werden. Ich sehe ja auch das die als geschädigte Bäume und als Totholz eine wichtige ökologische Funktion haben, da hat man sich einfach gesagt, wir sperren den Weg, um die alten Bäume erhalten zu können. Man hat da auch mit Hinweisschildern draußen versehen und hoffen da auf ein gewisses Verständnis, weil ich finde auch, die Leute sollten auch merken, dass dieser Waldzustand auch persönlich Auswirkungen für sie haben kann. Z.B. in der Form von Wegsperrungen, aber leider ist es auch so, dass die Schilder immer wieder weggerissen werden. Also die Leute trotzdem durchlaufen oder auch die Radfahrer durchfahren. Aber in dem Moment, in dem wir gesperrt haben, ist erstmal auf die Gefahr hingewiesen. Da muss man eben einen bunten Baukasten verschiedenster Maßnahmen haben und zusammensetzen. Schwarz-Weiß ist da völlig deplatziert.

A4

1	**A4 (16:01.6 Minuten)**
2	**LT:** Welchen Bezug haben Sie denn zum Karlsruher Stadtwald durch Ihre Arbeit?
3	**A4:** Ich bin Mitgliedin eines Ausschusses und da befassen wir uns so gut wie jedes Mal eigentlich damit, also bei jeder Sitzung. Da geht es um verschiedene Themen dann, was Bäume angeht. Also nicht nur die Trockenheit, sondern auch der Befall von diesen Borkenkäfern oder ich habe das andere Tier vergessen, wie das heißt, hat einen längeren Namen. Ja, so Sachen.
4	**LT:** Und was bedeutet für Sie naturnahes Waldmanagement?
5	**A4:** Das bedeutet für mich, dass auch Tothölzer liegen bleiben dürfen und nicht weggeräumt werden. Für mich bedeutet, dass das nicht das Augenmerk auf die Wirtschaftlichkeit des Waldes liegt, sondern dass der Wald so sein darf, wie er ist.
6	**LT:** Und dieses naturnahe Waldmanagement ist ja auch im Konzept von Karlsruhe zur Klimaanpassung, dass das angewandt wird. Wie würden Sie das bewerten, die Anwendung? Oder generell das Klimaanpassungskonzept von Karlsruhe zum Wald.
7	**A4:** Da müsste ich jetzt wissen, was dieses Konzept beinhaltet, das weiß ich aber jetzt leider so aus dem Stehgreif nicht mehr. Also ich glaube, ich habe es mir, ich weiß auf jeden Fall, um was es

	geht. Ich habe die Information nur nicht abgespeichert und soweit ich mich erinnern kann, habe ich das als nicht schlecht empfunden. Also da war jetzt nichts, was ich irgendwie dagegen hätte äußern können oder aufrufen, aufschreien müssen, weil da irgendwas nicht gepasst hat.
8	**LT:** Also, es wird dieses naturnahe Waldmanagement beschrieben und die verschiedenen Funktionen, die gefördert werden sollen und dann werden so Konflikte, wie die Baumartenauswahl für die Zukunft besprochen. Welche Konflikte sehen Sie denn in der Klimaanpassung des Stadtwaldes?
9	**A4:** Also ich kann mir gut, oder was heißt, gut vorstellen. Das Ding ist ja immer, dass Wissenschaftler*innen und Expert*innen quasi immer nur bis zu einem gewissen Punkt Dinge hervor sagen können oder herausberechnen können und im Endeffekt habe ich jetzt in meinen, sagen wir mal, in meinen Arbeitsjahren die Erfahrung gemacht, dass es am Ende doch noch schlimmer ist, als vorhergesagt und da habe ich ein bisschen Angst, dass auch diese Baumarten, die da neu miteinbezogen werden, weil die dann hitzebeständiger sind, Trockenheit gut aushalten können und so weiter. Dass das dann auch nicht reichen wird. Also, dass, wie soll ich sagen? Dass einfach auch diese Baumarten, dann keine Ahnung, dann siehst du irgendwann einen Pilz auch, der die dann befällt und so weiter. Also das weiß man ja alles vorher nicht. Und ich habe einfach die Angst, dass nicht so weit versucht wird in die Zukunft zu schauen, wie es eigentlich nötig wäre.
10	**LT:** Und welche politischen Instrumente sehen Sie für die Zukunft, um den Stadtwald vor dem Klimawandel zu schützen oder anzupassen?
11	**A4:** Also ich denke, dass wir in Karlsruhe gut aufgestellt sind was Experten, Expertinnen angeht und ich denke, am allerwichtigsten wird es sein, dass man die Politik vielleicht hintenanstellt und generell vielleicht mehr auf Expert*innen hört. Egal, ob das jetzt finanziell machbar ist, oder nicht. Wir wissen alle, dass die finanzielle Machbarkeit in Zukunft noch ganz anders ausfallen wird, als heutzutage. Also das ist nicht nur eine Investition für die Zukunft, für den Klimawandel. Das würde uns auch in Zukunft viel Geld sparen. Aber davon auch abgesehen, wie gesagt, ich finde einfach wir sollten mehr auf Expert*innen hören. Und deren, ich sage jetzt mal, Willen auch umsetzen.
12	**LT:** Wo sehen Sie die Konflikte, dass das jetzt noch nicht so sehr passiert?
13	**A4:** Die Konflikte sehe ich tatsächlich in der Wirtschaftlichkeit, also können wir und das leisten? Ab mitterechts, bzw. ab Mitte bis Rechts sind das ja die ganzen Parteien, die dann sagen, “Ja, Klimaschutz schon, aber nur, wenn wir uns das leisten können.” Für mich ein absoluter Bullshit. So, weiß ich nicht, was ich dazu noch sagen soll. Also bin ich absolut das Gegenteil davon.
14	**LT:** Dann würde ich jetzt zum zweiten Aspekt von meiner Masterarbeit kommen, und zwar die Auswirkungen der Covid-19-Pandemie auf den Stadtwald. Und zwar bietet der Stadtwald ja der Bevölkerung von Karlsruhe auch eine Erholungsfunktion und da gab es schon einige Umfragen oder vielleicht haben Sie es ja auch selber bemerkt, an Ihrem Wohnort, dass diese Erholungsfunktion seit Ausbruch der Covid-19-Pandemie vermehrt nachgefragt wurde, also mehr Menschen sind im Wald. Welche Konflikte sehen Sie dort?
15	**A4:** Also das reicht ja alleine schon, wenn man sich draußen aufhält, ob es jetzt kurz einmal zum Mülleimer gehen oder sonst irgendwas: es fliegt überall Müll rum. Überall ist alles zugemüllt, weil Masken fliegen rum, also ich habe noch nie so viele Masken auf dem Boden und irgendwie in Gebüschen und sogar in Baumkronen Tüten rumlümmeln sehen. Ich denke einfach, dass wenn die Menschheit in die Natur rausgeht, dass sie sich dieser auch verantwortlich zeigen müssen, indem Sie mindestens Ihren Müll mitnimmt. Vielleicht wäre da auch hilfreich einfach mehr Mülleimer aufzustellen. Ich glaube in Karlsruhe, also in einer nahegelegenden Gemeinde z.B. stehen bei so, ja wie soll ich sagen, Fahrradwegen, Wanderwegen, sage ich jetzt mal ganz blöd, da wo es eben in die Natur rausgeht, ist eben irgendwie alle 50 Meter steht da ein Mülleimer. Das ist gut, weil man da dann, je nach dem wo man gerade ist, einfach sein Müll in den Mülleimer werfen kann. Hier in meinem Wohnort verschwinden Mülleimer auch, habe ich das Gefühlt. Also bei mir hier auch im Wohnbezirk, wo ich immer auf gewissen Mülleimer angewiesen war. Ja, dass die Leute die Natur einfach nicht unbedingt nicht respektieren, aber einfach nicht *aware* für das

	Thema sind. In welchem Gleichgewicht das alles ist. Also nicht nur was Müll angeht. Dann nehmen die ihre Tiere vielleicht mit, also Hunde. Ich erlebe das auch immer bei einer Freundin, wenn ein Hund jetzt nicht immer stehen bleibt, wenn er sein Geschäft verrichtet und dann sucht man quasi das zweite und dritte Häufchen und dann bleibt das liegen, wenn man es nicht findet und das nächste Kind oder der nächste Mensch findet es dann, indem er da rein tretet. Und auch so Geschichten, wenn man jung und verliebt ist oder auch wenn man älter ist und dann mit seinem Taschenmesser dann die Initialen in die Baumrinde reinritzt. Ja lauter so Sachen, die der Mensch halt verkacken kann. Also ich sehe das schon problematisch an, wenn der Mensch in die Natur rausgeht. Ich finde sie sollten da einfach edukiert werden, wie man sich richtig in der Natur verhält.
16	**LT:** Wäre das dann von der Politik aus zu lenken? Oder wie sollte das angegangen werden?
17	**A4:** Also ich denke, dass die Politik da auf jeden Fall einen Schritt machen könnte, wenn natürlich die Mehrheit dann dafür da ist, weil das würde ja dann auch wieder Geld kosten, bla bla bla. Ich denke aber auch, dass z.B. Firmen oder Wohnungsgenossenschaften, dass die da alle eigentlich mitmachen könnten, weil das ist etwas, das nicht nur die Politik angeht, sondern das ist ein gesamtgesellschaftliches Ding und das ist dann egal, ob Privatwirtschaft oder Politik oder da, wo ich wohne, Privatvermieter*in oder Genossenschaften. Ich finde, das ist eine Aufgabe von uns allen. So *generally speaking*.
18	**LT:** Ziehen Sie Konsequenzen aus der Covid-19-Pandemie bei der Verwaltung des Stadtwaldes für die Zukunft?
19	**A4:** Dazu müsste ich erstmal wissen, was die Pandemie bis jetzt dem Wald angetan hat in Gänsefüßchen. Was ich allerdings noch nicht weiß. Wenn es der Fall ist, dass die Verschmutzung in die Höhe gegangen ist und so weiter, dann finde ich schon, dass das auch Konsequenzen haben muss. Ich weiß nicht, statt einfach nur darauf zu achten, dass die Menschen Abstand zueinander halten vom Ordnungsamt, für was haben wir das Ordnungsamt? Wie die Leute mit Müll z.B. umgehen.
20	**LT:** Dann nochmal etwas allgemeiner: mit welchen Indikatoren würden Sie die Qualität des Stadtwaldes messen?
21	**A4:** Ich denke ich würde das nicht nur, ob die Baumkrone jetzt noch funktioniert oder nicht, daran messen. Ich denke, ich würde auch gucken was die Allgemeine Population von verschiedenen Lebewesen angeht. Ob z.B. irgendwelche Insekten mehr oder weniger werden, Vögel mehr oder weniger. Ich weiß nicht, wie es teilweise mit Amphibien ist. So Geschichten einfach. Also dann ganzheitlich betrachtet gucken, wie sich alles weiterentwickelt und mit Blick in die Vergangenheit, versuchen in die Zukunft zu schauen.
22	**LT:** Jetzt sind der Klimawandel und Pandemien globale Probleme. Lassen die sich am besten auf lokalster Ebene lenken oder sollte sowas national oder sogar international angegangen werden?
23	**A4:** Ich glaube, es ist beides wichtig. Ich glaube, eigentlich müsste es international nicht unbedingt gelenkt werden, weil dann ist auf kommunaler Ebene vielleicht schwierig irgendwie handlungsbedürftigt zu bleiben. Aber ich finde, dass eigentlich international, aber es ist auch schwierig, gerade Polen, Russland, China. Also ich glaube, dass es Aufgabe von allen dreien Ebenen ist, definitiv. Je geschlossener, desto besser natürlich und es muss einfach auch von nationaler, internationaler Ebene mehr Geld zur Verfügung gestellt werden, weil die kommunalsten, die kleinsten, die können nur mit dem was sie zur Verfügung haben handeln und Maßnahmen in die Wege leiten, aber sie sind dann eben doch auf die Landes- und Bundes- und internationale Ebene, je nach dem, auch angewiesen. Aber ich finde, es ist ganz wichtig, dass man auch kommunal alles gibt, mehr als alles gibt, um einfach nicht nur das richtige Signal zu senden, sondern es bringt halt nichts, wenn man sagt: "wenn jeder an sich gedacht hat, ist an Alle gedacht", sondern man muss auch einfach wirklich von sich aus Dinge auch anpacken und so sehe ich das auch auf kommunaler Ebene.
24	**LT:** Jetzt hat es vor einem Jahr eine Reformierung der Forstverwaltung gegeben infolge eines Holzkartellverfahrens in Baden-Württemberg, also der Staatswald ist jetzt in Hand der Anstalt

	des öffentlichen Recht ForstBW und der Körperschaftswald in Karlsruhe vom Forstamt. Was ist da Ihre Meinung dazu?
25	**A4:** Können Sie das vielleicht näher erläutern, was das jetzt, was da die Änderung ist? Also wie kann ich mir das vorstellen?
26	**LT:** Also, zuvor war der Wald in der Gemarkung Karlsruhe in Hand des Forstamtes, also der Gemeinde. Und jetzt gibt es eine Anstalt des öffentlichen Rechts, die mehrere Wälder in ganz Baden-Württemberg bewirtschaftet, verwaltet und lenkt. Und dann haben die auch Teile aus der Gemarkung Karlsruhe.
27	**A4:** Zunächst einmal würde ich mich fragen, was für ein Projekt, das ist. Ob das direkt von einer Regierungspartei ist oder von den beiden Regierungsparteien oder zwei anderen Parteien? Je nachdem.
28	**LT:** Also der Koalitionsvertrag 2016, glaube ich, Grüne/CDU wurde das auch festgelegt.
29	**A4:** Uff. Ja jetzt kommt es halt darauf an, was kann die Kommune noch damit machen. Ich denke, je größer die Institution oder die Instanz, die dann die Federführung hat, desto problematischer ist es, weil die natürlich ganz andere Interessen wahrscheinlich haben, wirtschaftliche Interessen meistens leider. Und ohne jetzt genau Näheres zu wissen, würde ich sagen: “Naja, geht so, geht so.”
30	**LT:** Gut. Dann sind wir auch schon beim Abschluss. Gibt es noch etwas zu dem Thema, dass Sie loswerden wollen oder was Ihnen jetzt bei den Fragen noch eingefallen ist im Laufe des Gesprächs?
31	**A4:** Ich glaube eigentlich nicht. Ich bin froh, dass ich überhaupt zu jedem Thema irgendwie was sagen konnte.

SP1

1	**SP1 (26:07.4 Minuten)**
2	**LT:** Haben Sie denn eigentlich einen Bezug zum Stadtwald von Karlsruhe? Waren Sie da schon mal?
3	**SP1:** Ich war mal da, aber sonst sehr rudimentär und zwar waren es dann sonst immer die Beispiele von einem ehemaligen Arbeitskollegen, den kennen Sie wahrscheinlich nicht, das ist der Vorgänger von einem anderen Arbeitskollegen und der war früher im Forstamt in Karlsruhe und hat da immer Beispiele gebracht. Aus seinem Stadtwald. Insofern hatte ich da noch so ein bisschen noch was im Ohr, aber ich selber ich habe ja in einem Projekt einen Teil da gemacht. Auch mit einer Partner-Institution und da waren wir allerdings nicht im Karlsruher Wald sondern tatsächlich in den Auwäldern. Das heißt, überschwemmte Flächen waren das. Und da weiß ich nicht was ich vor allem vom ehemaligen Kollegen noch im Kopf habe, sind das ja sehr trockene, kiesige Flächen in Karlsruhe, aber da wissen Sie wahrscheinlich mehr als ich.
4	**LT:** Was bedeutet für Sie naturnahes Waldmanagement?
5	**SP1:** Ja gut. Naturnahes Waldmanagement. Das ist ja ein weites Ding. Das wird ja gerade definiert von irgendeinem EU Projekt, was das überhaupt sein soll. Und Sie sagen jetzt naturnah oder naturgemäß?
6	**LT:** Also ich meine in der Klimaschutzanpassungsstrategie von der Stadt Karlsruhe wurde es naturnah genannt.
7	**SP1:** Wurde es naturnah genannt. Ja.
8	**LT:** Oder was wäre jetzt für Sie naturgemäßes Waldmanagement?
9	**SP1:** Ja gut das sind ja mehrere Aspekte, die da dann eine Rolle spielen. Das ist die Baumartenwahl natürlich als Erstes, wo eigentlich überwiegend auf einheimische Baumarten gesetzt wird. Deswegen, ich bin mir da jetzt nicht ganz klar wegen naturnah und naturgemäß, weil wenn beim naturgemäßen ist es auch durchaus möglich glaube ich durchaus einen Fremdländeranteil drin zu haben. Und beim Naturnahen wird, das dann schon problematisch und da wird es dann auch für

	Karlsruhe dann irgendwann mal problematisch in nächster Zeit, wenn die Klimaprognosen stimmen. Ansonsten ist es die Vorgehensweise, das heißt die Dynamik vom Wald eigentlich ausnutzen mit möglichst extensiven Eingriffen und setzen auf Naturverjüngung ist dann natürlich ein großes Ding, wobei man auch in Karlsruhe haben wir da das Problem, da muss man ja letztendlich auf die Eiche setzen und die Problematik mit der Eichenverjüngung, die kennen Sie ja selber. Und wir haben ja hier im Arbeitsort ja ein sehr divers diskutiertes Beispiel mit dem örtlichen Eichenwald und wo der Naturschutz natürlich hingeht und sagt, diese benötigten Kleinkahlflächen für die Eichenverjüngung wollen sie da eigentlich nicht so haben und auch die Zielstolpennutzung, die die Zieldurchmesser deutlich in die Höhe setzen und der dortige, zuständige Forstamtsleiter der sieht das da etwas anders und auch ein Kollege, das weiß ich, der sieht das da etwas anders. Das der Wald doch jetzt zur Verjüngung ansteht und diese Vermittlung von diesem Konflikt vor allem dann auch an die Öffentlichkeit und auch die Forstzelte da ein bisschen zu erklären. Das ist ein ganz großes Ding. Und ich vermute mal, dass in Karlsruhe, das man da wahrscheinlich ähnliche Problematiken hat. Dann ist es natürlich Jagd. Ich glaub nicht, dass das in Karlsruhe eine Rolle spielt. So Stadt nah da weiß ich zum Beispiel überhaupt gar nicht, ob da überhaupt gejagt wird. Gejagt werden darf. Wenn ich mir den Plan von Ihnen angeschaut hab, welche Flächen das sind in Karlsruhe. Das sind ja fast schon Parks. Hätte ich jetzt gesagt. So wie das integriert ist von der Stadt Karlsruhe. Und das weiß ich nicht, ob Jagd möglich ist und wie das gehandhabt wird und dann mit der Problematik Naturverjüngung haben wir dann natürlich wieder so ein Stein den man wegrollen müsste und in Karlsruhe haben wir denke ich auch, was bei Naturnah nicht gern gesehen wird, wo auch gesagt wird, das wollen wir eigentlich nicht haben. Das heißt Einsatz von Insektiziden im Stadtwald. Das heißt, das weiß ich noch vom ehemaligen Kollegen, die Problematik mit dem Maikäfer und jetzt kommt sicher auch der Eichenprozessionsspinner dazu in Karlsruhe, die doch, wo man wahrscheinlich nicht um die Chemie im Wald rumkommt. Allein schon aus Gesundheitsgründen von der Bevölkerung. Ansonsten, naturnaher Waldbau, das heißt einzelstammweise Anbauen wenn es geht, aber das wird in den Eichenwäldern überwiegend gruppenweise gemacht, aber das sehe ich da jetzt nicht als problematisch an und vom anderen her, das übliche: Rückegassen und so weiter einhalten und da sehe ich eigentlich da keine Restriktionen, aber ich vermute mal, die größte Restriktion oder die größten Problematiken, das ist die Baumartenwahl. In Hinblick auf den Klimawandel, was wir dann haben.
10	**LT:** Und jetzt haben Sie schon ganz viele Probleme angesprochen. Haben Sie auch schon Lösungsansätze auf politischer Ebene, wie jetzt zum Beispiel Baumartenauswahl oder die Kommunikation haben Sie ja auch schon angesprochen?
11	**SP1:** Auf politischer Ebene. Also wir bewegen uns ja auf bundespolitischer Ebene, das wird da wahrscheinlich nicht um gebrochen werden können auf die kommunalpolitische Ebene, also auf jeden Fall ist es, dass ständige Kommunikation mit dem Bürger und Aufklären des Bürgers auch im Hinblick auf die Ziele des Gemeinderats und das weiß ich eben nicht oder der Stadtrat in Karlsruhe, wie der aussieht. Also was die Stadt Karlsruhe sich verschrieben hat. Ob sie die Nutzung gleichwertig sieht wie jetzt die Erholungsfunktion und wenn dem so ist, dann muss da massiv Kommunikation betrieben werden mit der Bevölkerung, um auch die Dynamik von der Erneuerung vom Wald, Umgestaltung vom Wald da eigentlich klar zu machen. Dass man da dann möglicherweise einer Nutzung nicht vorbeikommen. Und auch mit dem Einsatz von anderen Baumarten, das heißt in Karlsruhe. Da müssen Sie mich kurz aufklären: sind das diese kiesigen Standorte? Sehr trockenen Standtorte?
12	**LT:** Also der Stadtwald ist, da gehört auch der Rheinauewald dazu tatsächlich. Im Westen der Stadt und darüber der Hardtwald. Wie jetzt die Standortbeschaffenheit ist, habe ich gar nicht so richtig gerade nur eben Laubbaumstandorte auf jeden Fall.
13	**SP1:** Okay ja gut da haben sie natürlich, wenn es dann Richtung Rhein geht die Problematik mit dem Eschentriebsterben, die Esche fällt da aus. Die Naturschutzorientierten Leute wollen da natürlich vermehrt die Eiche drin haben, aber das ist ökonomisch gesehen natürlich sehr problematisch. Sehr kostenintensiv und auch im Hinblick auf die ganze Fraßgesellschaft im Auwald und auch in den höher gelegenen Bereichen ist eine riskante Sache, d.h. da kann schon die Eiche

	möglicherweise großflächig geschädigt werden bis zum Totalausfall. D.h. wir brauchen da irgendwo eine Risikoverteilung mit einer Mischung. Da ist die Frage: was können wir denn noch Reinmischen und dann sind wir wiederum bei der Frage: wollen wir, dass nur heimische Baumarten, da kommen relativ wenige in Frage, die dann auch noch ökonomisch attraktiv sind oder wollen wir doch ein paar Fremdländer. Ich weiß dann, wir waren in Karlsruhe im Nordwesten von Karlsruhe am Rhein und der dortige Revierleiter baut da durchaus auch die Platane an. Das ist eine sehr interessante Baumart, gerade auch auf diesen, weil es da doch auch Überschwemmungen und sehr trockene Verhältnisse erträgt und auch noch vom Holz her doch recht interessant ist. Also vom Holzpreis her. Ansonsten wird es auch auf die Schwarznuss und wenn man sie als einheimische Baumart tituliert die Walnuss rauslaufen zur Verbreiterung der Baumarten Palette oder dann Baumarten, wo man nicht ganz weiß, sind sie wirklich erfolgreich? Also für die Platane konnte man schon zeigen: das geht. Aber bei der zum Beispiel bei der Wildbirne oder was haben wir denn da noch? Bei dem Ahorn weiß man auch nicht wo die Reise hingeht, weil wir haben jetzt doch vehemente Ahornausfälle durch die Rußrindenkrankheit, also da wird man wahrscheinlich , um die Fremdländer mit einem bestimmten Anteil nicht rauskommen und man sagt ja auch man orientiert sich auch mit der naturnahen Waldwirtschaft der FSC Richtlinien da wär das dann auch kein Problem. Da könnte man ja auch 30% könnte man da zumindest mal reinbringen.
14	**LT:** Dann würde ich zum nächsten Themenblock schreiten. Und zwar zu der Covid-19-Pandemie und der steigenden Nachfrage nach dem Stadtwald. Also auch in der Stadt Karlsruhe gab es schon mehrere Umfragen, das die Leute zurzeit mehr in den Wald gehen oder in Freiburg sieht man das ja auch. Und da wollte ich Sie fragen welche Konflikte Sie dort sehen? Vor allem jetzt auch zukünftig, wenn man überlegt, dass solche Pandemien vielleicht jetzt auch öfter auftreten könnten. Wir haben jetzt viel auch über das naturnahe Waldmanagement geredet und dann eben auch gleichzeitig mehr Menschen im Wald.
15	**SP1:** Ja. Ein paar Sachen habe ich ja schon genannt, und zwar dass Eine, wenn ich den naturnahen Wald haben will und auf die natürliche Dynamik setze und Naturverjüngung drin haben will muss ich, brauche ich die Jagd. Und wenn ich dann mehr Besucher im Wald habe und dann zusätzlich noch die Jagd hab, dann ist die Frage: wie geht das? Und das wird dann sicher eine sehr spannende Sache. Ich weiß jetzt nicht wie das da gelöst wird, also in meinem Arbeitsort da sehe ich, da wird ein, zwei Mal im Winterhalbjahr schwerpunktmäßig eine große Treibjagd in diesem Bereich, wo der Erholungsdruck sehr stark ist, wird der abgehalten der wird halt [13:20.8?] und dann geht es da los, dann geht es da zur Sache. Wie die in Karlsruhe das machen wollen? Ich weiß es nicht und dann natürlich auch: in Karlsruhe gibt es deutlich mehr Wildschweine, also das heißt da dann zu jagen, also Wildschweine ist eigentlich nicht negativ, die fressen zumindest mal die Waldkäfer Engerlinge, aber da hat man wieder andere Problematiken: die Schweinepest rückt rein, und dann schaut man auch, dass die Wildschweinbesatzdichte möglicherweise deutlich geringer ist, wie in anderen Bereichen, wo weniger Besucher sind. Das ist das eine, bei den Besuchern. Das andere ist, wir haben dann vermehrt Schäden in den Kronen von den Bäumen, bei älteren Bäumen und dann steigt natürlich auch die Gefahr von herunterfallendem Totholz. Und wo dann doch die Leute immer dahinter sein müssen, also die Leute von der Stadt, das weg zu klopfen und dann auch dicke, ältere Bäume weg zu klopfen. Da sehen Sie ja die Problematik schon wieder. Dicke Bäume weghauen und aus Sicherheitsaspekten, das wird sicher ein spannendes Thema werden. Ob man dann tatsächlich dann diese Hubsteiger einsetzt für ein horrendes Geld oder ob man dann die Bäume tatsächlich entfernt. Dann heißt es auch "Besucherlenkung", das heißt wo ich dann auch bewusst diese alten Bäume lasse, Stichwort Habitatbäume und Altholzgruppen, dass ich dann schaue, dass möglicherweise diese Wege auch wenn die Besucher möglicherweise kreuz und quer durchlaufen, aber das ich doch eine gewisse Besucherlenkung habe. Ansonsten aus waldbaulicher Sicht mit dem Eichenprozessionsspinner, das hatten wir ja schon angesprochen, wo ich möglicherweise um einen Chemieeinsatz nicht rumkomme. Ich weiß jetzt nicht wie die Schwärmspinner in einem anderen Ort in Deutschland sind es ja zusätzlich noch die Schwarmspinner die bekämpft werden müssen, ob das in Karlsruhe noch eine Rolle spielt, aber das bleibt eine spannende Sache, vor allem auch im Hinblick darauf, dass das wahrscheinlichen den Klimaprognosen nach nicht besser wird in den

	nächsten Jahren, sogar dass man da eine neue Schädlinge, oder wenn man Schädlinge so bezeichnen darf, bekommt oder dass man stärkere Mutationen von den bestehenden Schädlingen bekommt. Das sehe ich eigentlich so als die Drei spannendsten Themenfelder im Hinblick auf Erholung und Besucherdruck im Wald.
16	**LT:** Dann nochmal ein bisschen allgemeiner: in welchen Kriterien würden Sie die Qualität des Stadtwaldes messen?
17	**SP1:** Kriterien hinsichtlich welcher Zielsetzung?
18	**LT:** Einfach die Qualität, ja, mein Thema ist dann schon Klimawandelanpassung und die Erholungsfunktion.
19	**SP1:** Ja ich denke schon, da kann man schon einige Kriterien vom naturnahen Waldbau aufgreifen. Das heißt, die Mischung, die Altersstruktur, die Schichtung, ich denke das. Und sicher spannend ist auch die Mortalitätsrate. von einzelnen Baumarten, die in den letzten Jahren eigentlich aufgetreten ist. Oder vor allem in den Trockenjahren jetzt aufgetreten ist. Und die Beurteilung von den einzelnen Baumarten hinsichtlich der Trockenheit Resistenz und was in Karlsruhe sicher noch eine große Rolle spielt, was ein bisschen unter den Tisch gekehrt wird, das ist die Hitze. Da muss man, das wird noch eine sehr spannende Sache. Und gerade in Karlsruhe hat man ja gesehen an diesen Kiefernbeständen, wo zumindest die Revierleiter vermuten, dass die starke Hitze, vielleicht auch Ozon, man weiß es nicht, dass da, dass dies die Bäume zum Einknicken bringt. Aber auf jeden Fall: Risikoverteilung, Mischung, Durchmischung. Das ist wahrscheinlich das A&O wo es wahrscheinlich drauf rauslaufen wird.
20	**LT:** Dann nochmal zum Politischen: und zwar sind ja Klimawandel und die Covid-19-Pandemie globale Probleme und wie denken Sie, lassen diese sich auf unterster oder lokaler Ebene am besten lenken?
21	**SP1:** Ja gut, also. Ich sage mal so bei Covid-19 da lass ich mich jetzt nicht aus, ich bin kein Mediziner und da fühle ich mich jetzt nicht unbedingt sehr kompetent. Klimawandel auf
22	**LT:** Also finden Sie es praktischer oder die Ziele leichter umsetzbar, wenn es sehr lokal und regional geregelt wird oder sollte mehr global agiert werden, größere Ziele?
23	**SP1:** Ich denke, auf der großen Ebene sollte so agiert werden, dass es lokal möglich ist zu agieren, d.h. die Unterstützung von regionalen Lösungen und nicht, also ich spreche jetzt für den Waldbau, und nicht immer mit der großen Gießkanne, das geht natürlich, wenn immer so große Schädigungen haben, wie wir es jetzt gesehen haben 2018/2019, wo man die Gießkanne auspacken sollte oder auch kann, weil dann auch große Flächen vorliegen, aber ansonsten sollte das auf jeden Fall mit Bundesgeldern möglich sein regional zu agieren und letztendlich muss das regional laufen und da muss jede Region und weil in jeder Region ist es ja auch anders. Da muss vor Ort entschieden werden: Wie kann ich den Wald bestmöglich da erhalten? Es geht bei uns vor allem jetzt mal um die Erhaltung der Ökosystemleistungen und nicht mal mehr der Verbesserung der Ökosystemdienstleistungen, sondern dass ich den Wald in einem bestimmten Zustand erhalte. Und gerade in den tieferen Lagen, auch wie der Waldbaureferent von einer Partner-Institution schon sagt, die gehen schon davon aus, dass da möglicherweise schon gar kein Wald mehr existieren kann, d.h. wenn es zu heiß wird und zu trocken, dass wir dann eine Verbuschung haben oder [21:04.4?] wie auf Korsika oder sonst wo, aber das man da lokal natürlich schaut, was sind die Möglichkeiten auf unseren Standorten. Das ist das A&O.
24	**LT:** Und jetzt zum Beispiel in Baden-Württemberg gab es ja ein Holzkartellverfahren, und bei dem ist ja der Staatswald von der Anstalt des öffentlichen Rechts ForstBW übernommen worden, wie sehen Sie da die Auswirkungen, auch im Hinblick dann Klimaziele zu erfüllen?
25	**SP1:** Im Hinblick auf den Karlsruher Wald, oder allgemein?
26	**LT:** Eigentlich schon Karlsruhe, aber wenn Ihnen allgemein mehr einfällt, können Sie auch dazu was sagen.
27	**SP1:** Ja ich meine, die kommunalen Wälder und die Staatswälder wird das nicht so betreffen, die sind vom Personal her eigentlich auch gut abgedeckt. Da sehe ich dann, es geht da vor allem auch um die Betreuung vom klein Privatwald. Und da ist es schon sehr extensiviert worden, ich sage mal

	so. Und das ist halt jetzt die große Diskussion, ob das mit privaten Dienstleistern überhaupt möglich ist, da eine adäquate Beratung reinzubekommen in den Kleinprivatwald, um den so anzupassen, dass er die Dynamik, die wir in den nächsten Jahren haben, dass der die mitmachen kann. Da sehe ich eigentlich das große Problem. Jetzt in diesen großen kommunalen Wäldern und Staatswäldern, also da tummeln sich so viele Fachleute, also da glaube ich da habe ich keine Bedenken, trotz diesem Kartellurteil. Also das berührt das ja letztendlich gar nicht. Und ansonsten die Vermarktung vom Holz vom Kommunalwald, ich glaub da sind die auch fit genug, also ich weiß nicht zum Teil läuft das ja immer noch über ForstBW, das hat man dann halt ausgelagert. Das ist dann halt ein Raum nebendran im Landesratsamt.
28	**LT:** Gut dann kommen wir auch schon zum Abschluss, gibt es noch generell etwas, was Sie sagen möchten zu dem Thema, was Ihnen noch eingefallen ist vielleicht?
29	**SP1:** Erholung. Ja ich denke spannend wird es gerade bei diesen Wäldern in der Rheinebene jetzt nicht unbedingt im Auwald, da wird sicher die Dynamik von den Hochwassern, bis sich das entwickeln wird, das wird sicher eine sehr interessante Sache werden, aber ansonsten und da sind Sie wahrscheinlich in der gleichen Lage wie Freiburg, die Mooswälder in Freiburg dass die Grundwasserentnahmen, von der Kommune, von den Betrieben, dass das noch ein größeres Konfliktpotenzial wird. Also da haben wir ja schon gesehen in Freiburg, wie sich der Grundwasserspiegel in den Mooswäldern abgesenkt hat und ich vermute mal, dass in Karlsruhe, das man da die gleiche Situation hat. Und da dieser Konflikt zwischen, das sehe ich dann eher, zwischen Gewerbetreibenden und Waldbesitzenden, Waldbewirtschaftern, dass man da einen ordentlichen Kompromiss schließen kann, ab wann dann auch keine Grundwasserentnahme mehr erfolgen kann. Und da sehe ich eine ganz große Volatilität eigentlich beim Karlsruher Wald. Ansonsten, dass was wir hier haben in den Hanglagen, diese Bodenprobleme, das wird es in Karlsruhe nicht. Spannend, wie gesagt, spannend ist das, was sich der Gemeinderat oder der Stadtrat letztendlich als Zielsetzung für die Wälder gibt und wie der Waldbau letztendlich drauf reagieren kann, diese Zielsetzung zu erreichen, letztendlich ist der Waldbau ja, ich sage mal, werteneutral, d.h. der muss nicht unbedingt Nutzen. Wenn man Prozessschutz oder Habitatbaumgruppen oder so etwas haben will, muss man halt gucken, wie man das waldbaulich so hinbekommt. Das ist sicherlich eine spannende Sache.

SP2

1	**SP2 (24:47.0 Minuten)**
2	[Dialog von 00:00.0-04:43.4 nicht transkribiert, um Anonymität zu wahren]
3	**LT:** Und was bedeutet für Sie Naturschutz beim Wald?
4	**SP2:** Naturschutz ist erstmal ein richtiges Instrument und was ein sehr gestuftes, gestufte Möglichkeiten vorsieht, Flächen zu schützen. Das Bundesnaturschutzrecht, die Landesnaturschutzgesetze sehen verschiedene Möglichkeiten vor. Was bedeutet das insgesamt? Ist natürlich immer etwas sehr Konkretes. Das kann man nicht abstrakt sagen. Ist hier etwas Notwendiges und ja auch schon etwas sehr Altes. Was schon vorm letzten Weltkrieg geschaffen worden ist als Instrument in einigen Staaten noch älter ist und was ich in der Summe so denke ich so sehr bewährt hat. Ich kenne auch Länder, in denen ein solchen Instrument nicht existiert oder nicht so ernst genommen wird wie in den Ländern des Nordens. Und da kennt man dann den Wert, den das hat. Das hat also tatsächlich einen sehr hohen Wert, weil die Interessen, die auf Flächen oder gegenüber Flächen bestehen, sehr vielfältig sind und dahinter sehr viel Macht steht. Und deshalb braucht man auch starke Instrumente dagegen.
5	**LT:** Welche zum Beispiel?
6	**SP2:** Was meinen Sie? Also "welche Instrumente dagegen" war jetzt das Naturschutzrecht. Aber z.B. auch das Waldgesetzt das sieht ja auch einige Instrumente vor. Also es gibt ja ein ganzes Paket

	von unterschiedlich wirkenden Instrumenten und die Interessen, die dagegen stehen sind natürlich vor allem Dingen ökonomische Interessen.
7	**LT:** Und wie bewerten Sie das aktuelle Klimaschutzkonzept, oder haben Sie sich damit schon mal auseinandergesetzt von der Stadt Karlsruhe zum Wald? Also Naturschutz findet da ja auch statt.
8	**SP2:** Im Wald? Ja natürlich, selbstverständlich und das ist auch notwendig, es gibt im Wald verschiedene Naturschutzgebiete. Der Wald hat selbstverständlich eine große, hohe Funktion im Rahmen von Klimaschutzmaßnahmen, das ist unstrittig. Und deshalb ist meine Bewertung, nach der Sie gefragt haben, dass die Bedeutung des Waldes für den Klimaschutz sehr hoch ist. \`Klimaschutz\` ist natürlich ein sehr schwieriger Begriff, wir müssen uns erstmal unterhalten, was das ist. Klimaschutz heißt eigentlich zunächst einmal: versuchen einen Status quo zu bewahren, das Klima vor Veränderung zu schützen. Das andere heißt, Klimaanpassung, indem ich versuche eine Gemeinde anzupassen an einen vorhandenen, erwarteten und prognostizierten Klimawandel. Und das soll eigentlich das Klimakonzept tun. Und da hat das Innerstädtische Grün insgesamt eine sehr hohe Funktion. Dazu gehört nicht nur der Wald, da gehören innerstädtische Parkflächen dazu, da gehören einzelne Solitärbäume dazu, da gehören Dinge wie Stadtbegrünung, Fassadenbegrünung, Dachbegrünung, und so weiter, sehr, sehr viel dazu. Und die Summe all dessen kann einen Beitrag leisten zur Adaption und es ist sicherlich nicht richtig dann ein Element nur herauszugreifen und das zentral zu betrachten. Die Aufgabe einer Klimaanpassung ist eine sehr integrale Aufgabe, die ja auch deshalb nicht nur vom städtischen Planungsamt oder vom städtischen Grünflächenamt oder vom städtischen Forstamt übernommen werden kann, sondern eine Querschnittsaufgabe darstellt, die von allen städtischen Ämtern, allen Aktiven getragen werden muss.
9	**LT:** Und welche Konflikte sehen Sie da zurzeit?
10	**SP2:** Das stößt selbstverständlich an sehr vielen Fronten an Konflikte. Erstens: an den Konflikt, dass die Stadt ökonomisch auf Zuwachs, auf Wachstum setzt. Wachstum braucht aber zunächst mal in meinem Verständnis -können wir lange drüber diskutieren- Fläche. Und diese Fläche ist also ein knappes Gut. Das führt automatisch dazu, dass Flächen vergleichend bewertet werden und da ist das traditionelle Denken, das halt landwirtschaftlich oder forstwirtschaftliche Nutzfläche eine Reservefläche für höherwertige ökonomische Nutzung darstellt, wie Bebauung, wie Verkehrsflächen oder sonstige Nutzflächen. Und das ist natürlich eine Gefährdung. Ganz allgemein formuliert: und genau diese Gefährdung vollzieht sich auch im Detail überall am Waldrand, am Siedlungsrand, überall. Man spürt das förmlich im Raum, wenn ich einen neugeschaffenen Siedlungsraum eines Bebauungsgebietes mir ansehe, dann ist das schon so angelegt, dass im Prinzip eine Aggression davon ausgeht weiter zu wachsen. Indem dort die entsprechenden Infrastrukturen schon so gelegt werden, zum Beispiel. Und da sieht man natürlich, dass Freiflächen generell sehr stark unter Druck stehen. Das sieht man randstädtisch, das sieht man aber auch innerstädtisch. Durch Dinge, die an sich sinnvoll erschienen und auch sinnvoll sind, wie die Nachverdichtung
11	**LT:** Ist das ein Lösungsansatz in Ihren Augen? Wäre die Nachverdichtung ein Lösungsansatz?
12	**SP2:** Nein, pauschal ist sie das mit Sicherheit nicht, sondern sie ist im Einzelfall, und das ist das Entscheidende, ein Ansatz. Aber pauschal zu sagen, die Nachverdichtung ist ein Lösungsansatz stimmt deshalb nicht, weil es viele Fälle gibt, wo bei einer Nachverdichtung die negativen Folgen überwiegen. Und diese Fälle gibt es eben, aber sicher ist es richtig, dass die Nachhaltigkeit als eine Idee miteingebracht worden ist. Weil die vorhergehende Planungs- -ich sag mal etwas abschätzig- Ideologie sehr stark darauf ausgerichtet war, das Wachstum nach außen zu fördern, die Suburbanisierung zu fördern. Und deshalb war es richtig irgendwo mit flächensparendem Planen und Bauen anzufangen, und dann anschließend mit Nachverdichtung, wobei Nachverdichtung viele Probleme schafft, auch zum Beispiel, gegen wechselseitige Störungen von Nutzung und so weiter, der Verlust von Freiflächen, der Verlust von wohnungsnahmen Erholungs- oder Wohnergänzungsraum und so weiter und so fort. Und trotzdem ist es gut, aber deshalb kann es nicht pauschal gut sein.
13	**LT:** Oder sehen Sie noch andere Lösungsansätze?

14	**SP2:** Die muss es geben. Ich denke, wir müssen grundsätzlich mal die Frage stellen, ob wir unsere Verdichtungsgebiete immer weiter wachsen lassen können. Dieses quantitative Wachstum, immer mehr Zuwachs, stößt natürlich irgendwann an seine Grenzen. Und dann überwiegen die negativen Effekte, während gleichzeitig Räume entleert werden. D.h. diesen landesplanerischen Ausgleich zwischen Wachstums- und Schrumpfungsräumen, den haben wir noch nicht im Griff. Da ist tatsächlich noch viel zu machen. Denn wir haben ja Flächen! Wir haben Baugebiete, die nicht genutzt werden; wir haben Wohnungen, die leer stehen in Deutschland in gar nicht unerheblichem Umfang. Und ich denke, da braucht man die Entwicklung nicht nur aus der Sicht einer Gemeinde in einem Ballungsraum, wie etwa Karlsruhe sehen, sondern, da muss man tatsächlich landesplanerisch das gesamte Land im Blick haben.
15	**LT:** Dann würde ich jetzt an der Stelle zum nächsten Thema meiner Masterarbeit gehen, und zwar, das war jetzt eher der Klimawandel und dann guck ich mir noch die Auswirkungen der Covid-19-Pandemie an. Und zwar sind ja jetzt immer mehr Leute, vor allem im letzten Frühjahr in den Wald gegangen und der Waldbesuch -haben Sie ja auch schon angesprochen- eine sehr große Erholungsfunktion bietet und ich wollte fragen, ob Sie da Konflikte sehen, die jetzt z.B. auch in Karlsruhe auftreten können, wenn jetzt immer mehr Leute den Wald besuchen wollen?
16	**SP2:** Da ist mir im Moment nichts geläufig, wo es da Konflikte geben könnte. Auf jeden Fall ist die Nutzungsintensität des Waldes in Karlsruhe nicht so, dass es dadurch durch diese Nutzung Konflikte geben müsste. Ich denke etwa an den südlichen Hardtwald da ist die Nutzung relativ intensiv, also vom Schlossgarten ausgehend. Konflikte gibt es dort nicht. Natürlich gibt es Ansätze von Verschmutzung, natürlich gibt es Probleme, die einige sehen von Banalitäten wie Hundekot und so etwas. Alles Mögliche, aber da würde ich nicht von Konflikten sprechen. Durch die Nutzung des Waldes nicht. Also wenn es als geregelte Nutzung erfolgt und wir reden hier nicht von Konflikten, die etwa zwischen Mountainbikern und Fußgängern oder Wanderern bestehen, sondern Konflikte durch die Zahl der Nutzer. Und das zeigt mir sehr gut, dass tatsächlich die Kapazität des Waldes für Erholungsnutzende sehr groß ist und das ist etwas sehr Erfreuliches, das man so etwas hat, die Bevölkerung in Karlsruhe hat, das ist nämlich nicht überall vorhanden. Der Waldanteil ist vor allem durch seine Lagebedingungen immer noch relativ groß.
17	**LT:** Und welche, wie kann sowas politisch noch gefördert werden? Oder was meinen Sie, welche Strukturen auch noch im politischen Rahmen gegeben werden, dass Karlsruhe eben eigentlich das so gut anbietet?
18	**SP2:** Nun gut. Das ist ja nicht das Verdienst der gegenwärtigen Generation, sondern etwas, das aus der historischen Entwicklung entstanden ist, indem Karlsruhe gegründet worden ist auf einer Fläche, die extrem schwierig landwirtschaftlich nutzbar war, deshalb war da wüster Wald und deshalb ist die Stadt dort gegründet worden. Und das unterscheidet die Stadt ganz wesentlich von anderen Städten, die inmitten landwirtschaftlicher Gunstlagen gegründet worden sind. Und dann wachsen konnten oder gewachsen sind sehr stark. Denken Sie an Städte wie Hannover oder so etwas, das ist ja überhaupt nicht vergleichbar. Und diese besondere Situation gibt natürlich ein Potenzial. Auch dadurch, dass in der Mitte ein Schloss ist und sich an das Schloss ein Schlosspark und ein Hardtwald als Jagdgebiet anschließt, was eigentlich eine unbegrenzte Erholung eben Stadt nah eröffnet. Das ist eine Gunst, die haben wenige Städte in Deutschland und darüber hinaus. Und das ist, glaube ich, auch nicht strittig, dass das erhalten wird. Allgemein. Also, niemand möchte das, aber es gibt natürlich immer wieder die Versuche, aufgrund der Flächenknappheit, die besteht dort ranzugehen und zu sagen: noch eine Straße, noch eine Häuserzeile, da brauchen wir noch eine Fläche und das passiert täglich. Jeder sieht das immer noch als ein Potenzial auch meine eigene Institution. "Können wir nicht hier auf der Fläche noch was bauen?" Eigentlich schon ökologisch im Hardtwald drin. Und dann sagt man, "Ja diese drei Bäume, die da gefällt werden, die machen ja auch den Wald nicht kaputt." Das ist richtig. Erstens bleibt es nicht bei den drei Bäumen und Zweitens haben wir natürlich auch drei Bäume eine Funktion. Aber so wird immer wieder argumentiert, wird auch argumentier, wenn es um eine Straße geht, auch wenn das mittlerweile schwer geworden ist sowas dann durchzusetzen, aber tatsächlich hat man Straßen aufgrund ihrer diversen Effekte, die sie haben eine negative Wirkung auf den Wald. Das ist der Zerschneidungseffekt, das ist der

	Verlärmungseffekt und, und, und, ich brauche Ihnen das nicht zu erzählen, aber aus diesem Grunde ist es natürlich erforderlich auch immer wieder den Wald in Schutz zu nehmen von der Planungsseite her, denn die anderen Nutzungen haben eine gute Lobby: ein Straßenbauamt, ein privater Investor, der Flächen nutzen möchte, die haben schon eine starke Lobby und gute Instrumente, um ihre Interessen durchzusetzen. Und deshalb ist es notwendig, dass man von der Verwaltungsseite her oder von der Gutachtenseite her oder von der Sachverständigenseite her, sich für den Erhalt diesen Waldes und seiner Funktionen, also funktionsbezogene Erhaltung nenne ich das, einsetzt.
19	**LT:** Sind Sie denn also eher dafür, dass so globale Probleme, wie Klimawandel schon lokal, also auf kleinerer Ebene geregelt werden sollte? Also jetzt auch solche Konflikte wie Ökonomie und Ökologie gegeneinander, oder sollte das mehr auf die globale Ebene herauf gehen?
20	**SP2:** Nein, es muss auf der lokalen Ebene geregelt werden, es kann gar nicht auf der globalen Ebene geregelt werden. Was sollen wir denn da regeln?
21	**LT:** Aber international?
22	**SP2:** Da geht wenig. Das kann man eigentlich gar nicht. Man muss die Nutzungsentscheidungen muss ich doch vor Ort treffen. Ich will doch nicht, dass die Flächennutzungsentscheidungen am Rande von Karlsruhe Hardtwald etwa irgendwo zentralisiert in Berlin oder in Brüssel oder vielleicht international in New York getroffen werden. Das ist doch Unsinn. Die Entscheidung letztlich, muss lokal erfolgen. Und alles was auf der internationalen Ebene getroffen wird, kann, allgemeine Regeln sind vielleicht Richtwerte, sind vielleicht Grundsätze, wie mit dem Freiraum umgegangen wird, aber mehr auch nicht. Das Ausführen, das konkrete Gestalten kann nur auf der lokalen Ebene erfolgen. Alles andere führt zu großem Unsinn. Es gibt Beispiele auf der Welt davon: also jedes ägyptische Dorf lässt sein Flächennutzungsplan bei einer zentralen Behörde in Kairo machen, das ist verantwortlich dafür, dass dort die Raumentwicklung so chaotisch ist, das ist im Iran genauso. Jede iranische Stadt bekommt ihren Flächennutzungsplan in Teheran gemacht. Das sind Dinge, die sind sowas von unsinnig, was dann rauskommt, da können wir nur froh sein, dass wir ein System haben, in dem das nicht so ist.
23	**LT:** Und jetzt zum Beispiel die Gewaltenumverteilung in Baden-Württemberg, also dass jetzt 2020 erst der Staatswald von der Anstalt des öffentlichen Rechts ForstBw übernommen wird und der Körperschaftswald jetzt nur noch beim Forstamt der Stadt ist, was ist da Ihre Meinung dazu?
24	**SP2:** Also ich sehe da noch nicht, dass das Auswirkungen auf den Stutzstatus des Waldes hat. Das ist aus meiner Sicht eine Verwaltungs- /Organisationsentscheidung, die kann man so treffen, die kann man nicht so treffen, da gibt es verschiedene Modelle, aber das bedeutet aus meiner Perspektive noch lange nicht, dass der Wald in der Planung einen anderen Stellenwert bekommt.
25	**LT:** Und wie würden Sie die Qualität des Stadtwaldes messen? An welchen Kriterien zum Beispiel?
26	**SP2:** Na gut. Wald, das habe ich einleitend gesagt, das ist was polyfunktionales. Wald hat eine wirtschaftliche Funktion, Wald hat eine Naturschutzfunktion, Wald hat eine Erholungsfunktion, Wald hat eine Wasserschutzfunktion und so weiter und so fort, bis hin zur Klimaadaption. Also Wald hat sehr viele Funktionen, die Waldfunktionspläne in ihren vielfältigen Funktionen beschreiben das ja auch. Und aus diesen Funktionsplänen werden dann ja gerne die Wirtschaftspläne abgeleitet. Also, der Wald hat sehr viele Funktionen und diesen jeweiligen Funktionen angemessen, muss er natürlich aufgebaut werden. Ein Erosionsschutzwald in Hanglage hat natürlich einen ganz anderen Aufbau, als ein stadtnaher Erholungswald. Endsprechend sind die Wälder angepasst und sie sind nicht nur an eine Funktion angepasst, sondern meist an sehr viele Funktionen, die sich ergänzen. Das mach andererseits auch die Vielfalt der Wälder aus. Und das ist etwas, was ich sehr positiv bewerte, wiederum im internationalen Vergleich. Gucken Sie sich den nordamerikanischen-, kanadischen Wald an oder noch viel extremer, gucken Sie sich einen chilenischen Wald an. Das chilenische Forstmodell sagt, der Waldwirt ist Privatbesitz und der Wirt verkauft an eine Firma, Forstfirma, das sind meist große internationale Konzerne, [23:48.5?] und die legen den dann an als Waldplantage. Was Grausameres kann man sich gar nicht vorstellen, als eine Waldplantage. Das nennen die genauso Wald, wie bei uns. Und auf der Karte sieht das genauso

	aus, das ist was grundsätzlich Anderes. Und in diesem Vergleich ist unser Modell der Waldnutzung ein sehr gutes. Und entsprechend gefällt mir auch der Wald, entsprechend führe ich auch z.B. lateinamerikanische Kollegen sehr gerne durch den deutschen Wald, den Schwarzwald.
27	**LT:** Gibt es zum Abschluss noch etwas, was Sie noch sagen möchten oder was Ihnen noch zwischendrin eingefallen ist, was Ihnen wichtig ist?
28	**SP2:** Nein, eigentlich nicht

SP3

1	**SP3 (16:43.9 Minuten)**
2	**LT:** Haben Sie denn eigentlich überhaupt einen Bezug zum Karlsruher Stadtwald?
3	**SP3:** Ja. Ich habe dort einmal ein Projekt gemacht, also im Rahmen von meiner Arbeit
4	**LT:** Aber selbst dort waren Sie auch noch nicht?
5	**SP3:** Doch ich war ein paar Mal dort. Also ich habe da gearbeitet und habe mich dort auch mit dem Forstamtsleiter getroffen und habe dort ein paar Begänge, Treffen, Meetings gehabt, ja. Jetzt aktuell läuft im gleichen Projekt, das eine Fortsetzung bekommen hat, wieder etwas nach dem gleichen oder nach einem ähnlichen Prinzip.
6	**LT:** Was bedeutet für Sie Naturschutz? Besonders im Wald?
7	**SP3:** Ja, also naturschutzfachlich bin ich ja gar nicht, kenne ich mich gar nicht aus. Das habe ich gar nicht gelernt oder studiert. Mir begegnet Naturschutz immer wieder einfach im Zusammenhang mit dem Dreiklang, also dass bei der Waldbewirtschaftung sowohl wirtschaftliche Aspekte, als auch Artenschutz und Nachhaltigkeit so, wie der Erholungswert berücksichtig werden muss. Mit dem Naturschutz an sich kenne ich mich nicht aus. Also, dass es schützenswerte Arten gibt und so, das weiß ich, aber da weiß ich gar nicht was ich dazu sagen kann.
8	**LT:** In meiner Masterarbeit arbeite ich zweigleisig, also ich schau mir einmal die Klimaanpassungsstrategie an und dann wie es im Hinblick zu der Situation von der Covid-19-Pandemie, dann vielleicht auch in der Zukunft, wenn sowas nochmal auftreten sollte, wie die Erholungsfunktion des Waldes gefördert werden kann politisch. Und Sie sind ja jetzt eher bei der Ökosystemdienstleistung?
9	**SP3:** Ja Ökosystemdienstleistungen, das sind, da habe ich mich mit den kulturellen Ökosystemdienstleistungen beschäftigt. Also mit diesen Werten im Wald wie Erholung, Schönheit, Ästhetik. Solche Dinge. Also quasi der Erholungsfunktion, dem sozialen Wert.
10	**LT:** Und welche Konflikte sehen Sie dort, bei der Erholungsfunktion?
11	**SP3:** Zum Naturschutz?
12	**LT:** Zum Beispiel, ja.
13	**SP3:** Also zunächst fällt mir ein bei der Erholungsfunktion, da gibt es viele Konflikte innerhalb dieser Kategorie. Also, viele Konflikte zwischen verschiedenen Waldbesuchenden. Die Waldbesuchenden stören sich gegenseitig beim Waldbesuch, die mögen sich nicht oder bei meinen Projekten ist immer herausgekommen: der größte Störfaktor beim Waldbesuch sind die anderen Menschen, die dort sind. Die dort irgendwie vielleicht anderen Aktivitäten nachgehen, also die Wanderer beschweren sich über die Fahrradfahrer, über die Mountainbiker und umgekehrt. Also Konflikte zwischen den Nutzergruppen und ansonsten. Naturschutzfachlich: ich denke, dass manche Leute es vielleicht nicht mögen, wenn manche Flächen vielleicht gesperrt sind oder geschützt sind, wenn da der Zugang eingeschränkt wird. Und dass da viel Öffentlichkeitsarbeit gemacht werden muss. Also auch bei Fällungen, also auch wenn man jetzt diese wirtschaftliche Funktion betrachtet. Muss viel Kommunikation, Öffentlichkeitsarbeit gemacht werden, D.h. wenn jetzt naturschutzmäßig oder forstwirtschaftlich eine Fläche genutzt wird, dann müssen die Leute darauf aufmerksam gemacht werden und hingewiesen werden. Und

	manche wollen auch genau erklärt bekommen: Was passiert denn da? Und nicht einfach nur eine Absperrung und das war es. Sondern schon eine Erklärung dazu. Natürlich interessiert sich nicht jeder Waldbesuchende dafür oder es ist eine Minderheit, die sich für diese Details interessiert, aber es gibt welche, die sich damit sehr beschäftigen und die sich daran sehr stören, wenn sie nicht aufgeklärt werden, wenn ihnen nichts erklärt wird.
14	**LT:** Könnten Sie jetzt auch schon Konsequenzen aus der Situation von Covid-19 z.B. ziehen, also dass jetzt vermehrt Leute in den Wald gehen?
15	**SP3:** Ja. Also es ist so, dass Kollegen von mir ein Projekt gemacht haben, speziell zur Covid-19. Ob der Wald mehr genutzt wird und inwiefern mehr als vorher genutzt wird. Ich glaube, die haben herausbekommen, dass der Wald mehr genutzt wird durch Covid-19. Das ist einfach ein Raum, den man noch besuchen kann, wo man sich noch treffen kann, wo ausreichend Platz noch vorhanden ist. Also ich glaube der Wald hat schon Zulauf bekommen. Und die Kollegen haben dort eine Umfrage gemacht und die Details von den Ergebnissen, die kann ich Ihnen jetzt gar nicht so genau sagen. Aber ich habe Ihre E-Mail weitergeleitet. Also vielleicht hat einer von den Kollegen auch noch Lust sich interviewen zu lassen.
16	**LT:** Okay, danke! Ich habe mich auch schon bei anderen Leuten gemeldet.
17	**SP3:** Also da gibt es [06:10.9 - 06:20.6 nicht transkribiert, wegen Anonymität]. Der eine hat sich viel mit der Covid-19-Pandemie beschäftigt. [06:23.4 - 06:30.6 nicht transkribiert, wegen Anonymität]
18	**LT:** Okay, danke. Und sonst so allgemein die Erholungsfunktion: wie könnte die auf politischer Ebene noch gefördert werden für den Wald?
19	**SP3:** Es gibt ein Interesse an Mitsprache. Grundsätzlich die Erholungssuchenden, die wollen, oder darunter gibt es einige, die gehört werden wollen oder die das Gefühl haben, sie möchten miteinbezogen werden bei der Planung und sie möchten auch gehört werden. Also so der Partizipationswille ist irgendwie da. Und da muss man, denke ich, diesen Waldbesuchenden irgendwie eine Plattform dafür geben. Dass die ihre Anliegen irgendwie kundtun können. Das die irgendwie sagen können "das und das stört mich" oder "das finde ich gut" oder "hier könnte noch dieses und jene gefördert werden", also es braucht irgendwie ein Sprachrohr oder eine Plattform. Und natürlich ist es schön, wenn es im Wald ein vielfältiges Angebot geben kann, also für die verschiedenen Nutzergruppen, dass die sich nicht so sehr bei ihren verschiedenen Aktivitäten stören. Wenn das allerdings so räumlich entzerrt wird und die einen Nutzergruppen mehr dort sind und die anderen Nutzergruppen mehr dort, das tritt natürlich in Konflikt mit dem Wald, mit der Holznutzung. Mit ökonomischen Aspekten, weil die, die im Wald Holz machen, die stört das natürlich, wenn alle Flächen von Erholungssuchenden belegt sind. Die freuen sich, wenn es solche Erholungs-Hotspots oder auch nicht so viel los ist. Weil dann wissen sie, okay, hier kann ich Holz machen und dort guck ich, dass es schön ist.
20	**LT:** Vielleich noch zum Abschluss: wie würden Sie die Qualität eines Waldes messen?
21	**SP3:** Da gibt es ja sämtliche Hinsichten. Also ich denke, die Qualität eines Waldes ist dann hoch, wenn er eben diesen verschiedenen Interessen irgendwie möglichst gut in Einklang bringt. Also sowohl von den Waldbesuchenden, dass die zufrieden sind, als auch das seltene Arten geschützt werden können, aber auch Geld verdient werden kann. Also wenn das irgendwie alles zusammenklappt. Das ist jetzt aber eine sehr oberflächliche, abstrakte Antwort, oder? Da ist jetzt schon mehr Detail gefragt, oder?
22	**LT:** Nein, das ist so auch okay.
23	**SP3:** Also ich weiß nicht, der Wald muss einfach. Dass einfach möglichst viele ihre Freude am Wald haben und möglichst viele Menschen von Wald profitieren können.
24	**LT:** Und jetzt zum Beispiel auch zu der Kommunikation oder zur Lösung der Probleme bei der Förderung von der Erholungsfunktion, was wir angesprochen hatten, sind das mehr die Lösungslenkungen auf lokaler Ebene oder auf internationaler Ebene?
25	**SP3:** Das sehe ich eher lokal. Ich denke, diese ganzen Wälder und Städte und die Kommunen, die an den Wäldern liegen, die sind immer so einzigartig, ich finde das ist schwer die alle über einen

	Kamm zu scheren. Und natürlich kann man Forschung machen und dann vielleicht ein bisschen allgemeiner herausfinden, dass irgendwie die Zugänglichkeit zu Seen und Gewässern gut ist für die Erholungssuchenden und er Zugang zu, ich weiß nicht, Beständen mit viel Laubbaumanteil oder solche Dinge, ja solche ganz groben Sachen kann man vielleicht allgemein sagen, aber ich glaube, das hilft den einzelnen Fällen lokal, vor Ort nicht. Lokal, vor Ort ist es so, dass die Bevölkerung gerne gehört werden möchte, oder dass es einen Teil der Bevölkerung gibt, die gehört werden wollen und da muss man auf die ortsspezifischen Eigenschaften eingehen und kann nicht so allgemein, allgemeingültige oder internationalgültige Sachen finden oder durchsetzen.
26	**LT:** Und dann gab es ja gerade in Baden-Württemberg eine Gewaltenumverteilung, also dass jetzt der Staatswald von der Anstalt des öffentlichen Rechts ForstBW übernommen wurde und der Körperschaftswald im Beispiel von Karlsruhe nur noch vom Forstamt gelenkt wird. Was ist da Ihre Meinung zu?
27	**SP3:** Ich denke, dass da die Sachen komplizierter werden. Aber mit Forstpolitik kenne ich mich nicht gut aus. Aber ich denke, also ich habe in meiner Arbeit die Erfahrung gemacht, dass es mehr Aufwand macht, man muss mit mehr Akteuren sprechen und mediieren auch. Also die haben unterschiedliche Bedürfnisse, Anliegen und Interessen und man muss diesen gerecht werden. Vorher hat man vielleicht mit einem Akteur gesprochen oder mit ForstBW nur und jetzt müssen die eben gleichermaßen angesprochen und berücksichtigt werden und es muss ein Konsens gefunden werden. Und so Zuständigkeiten sind noch nicht geklärt oder das dauert alles bis dieses System so etabliert ist. Aber es gibt auch Chancen. Also, ja es ging doch darum, dass auch andere Waldbesitzende in Konkurrenz treten können?
28	**LT:** Genau dieses Holzkartellverfahren war das.
29	**SP3:** Ansonsten abgesehen davon, dass es natürlich ein Vorteil gibt, denke ich schon auch, dass es Sachen komplizierter macht, der Verwaltungsaufwand, Kommunikations-, Abstimmungsaufwand höher wird.
30	**LT:** Dankeschön. Gibt es noch irgendetwas, was Sie noch loswerden möchten oder ist Ihnen noch Irgendetwas eingefallen im Laufe des Gesprächs?
31	**SP3:** Nein so allgemein fällt mir da jetzt nichts ein. [14:16.1 - 14:55.5 nicht transkribiert, wegen Anonymität]
32	**LT:** Aber ich habe jetzt auch schon gemerkt, dass es nur in eine Richtung von meiner Arbeit geht und der Klimawandelaspekt ist gar nicht so in Ihrer Arbeit drin?
33	**SP3:** Ja da bin ich einfach keine Expertin drin. Da kann ich nicht so viel sagen, also das ist in meinem Feld etwas, das mitberücksichtigt wird, aber wo ich mich nicht zu auskenne und ich weiß, das infolge des Klimawandels oder der Erwärmung neue Arten in Betracht gezogen werden und die Rolle der Douglasie, die jetzt die Fichte ersetzen kann und was ihre Vor- und Nachteile sind und solche Dinge, aber da kann ich nicht so hilfreich sein glaube ich, aber das könnte vielleicht eine Kollegin. Okay also es ging jetzt ja darum irgendwie Erholung und, also die Erholungsnutzung in der Pandemie und Artenschutz, oder im Zusammenhang mit dem Klimawandel? Da konnte ich nicht so hilfreich sein.

SP4

1	**SP4 (27:39.0 Minuten)**
2	**LT:** Wie ist denn Ihr Bezug zum Karlsruher Stadtwald?
3	**SP4**: Ich bin selber kein Erholungs-Suchender im Karlsruher Stadtwald, ich bin eigentlich nur sporadisch da, ganz viel eher im Bereich Hardtwald. Aber in meiner Position hat man immer so ein bisschen den Blick auf den gesamten Wald in Baden-Württemberg und da sind wir natürlich insgesamt beim Stadtwald Karlsruhe im Bereich vom Wuchsgebiet 1, sind also tiefe Lagen, mit

	warmen Temperaturen und natürlich in einem urbanen Umfeld. Ganz klar ist die Dominanz einfach die Erholungsfunktion und die Schutzfunktion. Mein Bezug ist so, dass ich häufiger an einer Institution in Karlsruhe bin oder gelegentlich auch mal beim - wie heißt es nochmal - einer anderen Institution oder so. Da bin ich manchmal für Fortbildungsveranstaltungen und im Wald selber bin ich gelegentlich unterwegs, weil wir da Versuchsflächen von der Arbeitsstelle aus haben. Das sind dann aber eher punktuelle Sachen, ansonsten sind natürlich die Revierleitenden und die Forstamtsleitenden vor Ort zuständig. Ich bin ja Gast.
4	**LT:** Was bedeutet für Sie naturnahes Waldmanagement?
5	**SP4:** Der Begriff der Naturnähe ist relativ klar definiert. Dass wir Waldzusammensetzungen anstreben, die der potenziellen natürlichen Vegetation sehr nahekommen, das ist eine Definition einfach so die festgelegt wurde. Und diese potenzielle natürliche Vegetation spiegelt einen bestimmten Vegetationszustand aus der Geschichte unserer Landbedeckung wieder. Derzeit ist das die neunte Stufe nach [02:13.0?] eine wissenschaftliche Einsortierung, die überwiegend auf Pollenanalytik beruht. Und das bedeutet, Naturnähe für mich bedeutet erstmal eine große Übereinstimmung der Baumartenzusammensetzung des heutigen Zustands mit diesem damaligen Referenzzustand. Ein sehr, sehr wichtiges Element ist das, und es ist auch ein sehr, sehr wichtiges Element was wir unter Klimawandel revidieren müssen, weil wir die Statik dieses Bezugssystems so nicht einfach blind in die Zukunft schreiben können. Neben der Naturnähe der Baumartenzusammensetzung sind auch andere Elemente wichtig für naturnahe Waldbewirtschaftung. Das ist, dass wir Bewirtschaftungsmaßnahmen natürlich sanft und Ökosystem-verträglich ausführen. Da gehört alles mögliche dazu: Da gehört dazu, dass wir die Erschließung von Wäldern für Holzerntemaschinen pfleglich durchführen, dass es da nicht zu Gleisbildung und großen Fahrspuren kommt, natürlich dass keine Biotope zerstört werden, dass wir Waldnaturschutz als integralen Bestandteil betreiben - also nicht Totalreservate von der Bewirtschaftung grundsätzlich ausnehmen, das möglicherweise punktuell schon auch, aber dass wir auch im Wirtschaftswald integrale Naturschutzelemente weiterverfolgen. Das ist ein wichtiges Kennzeichen der naturnahen Waldwirtschaft. Und natürlich dann auch, dass was wir mit der Holzernte zerstören, das heißt alte und auch tote Bäume, dass wir die punktuell erhalten in bewirtschafteten Wäldern. Das machen wir im Landeswald üblicherweise mit Hilfe des alten Totholzkonzeptes so, wo wir ganz bewusst auch kleinere Baumgruppen (10-12 Individuen oder ähnliches) markieren und die für die Altersphase oder die Zerfallsphase erhalten, damit eben die xylobionten Arten und alle Arten in Lebensgemeinschaften auch die angewiesen sind auf Tothölzer, damit die auch systematisch im gesamten bewirtschafteten Wald vorkommen. Ein weiteres wesentliches Element der naturnahen Waldwirtschaft. Und so könnte ich glaube ich noch sehr sehr lange weiterreden.
6	**LT:** Dann kommen wir mehr zum Politischen: Und zwar hat sich die Forstverwaltung von Karlsruhe dem naturnahen Waldmanagement unterlegen sozusagen und hat auch das Konzept zur Klimaanpassung für den Stadtwald festgelegt, was auch diese Regeln und Ziele des naturnahen Waldmanagements - was Sie jetzt auch viel genannt haben - verfolgt. Da wollte ich fragen, wie Sie, wenn Sie sich da jetzt auskennen, die Ausführungen zurzeit bewerten würden?
7	**SP4:** Also die Ausführungen wie das jetzt umgesetzt wird, dieses Konzept, das kann ich überhaupt nicht beurteilen. Dazu würden mir die Kriterien und Datenhintergründe völlig fehlen. Ich habe aber interessanter Weise mich mit diesem Konzept vor wenigen Wochen mal auseinandergesetzt als es mit einer Politikerin oder so, oder den Namen habe ich nicht mehr ganz parat, einer Bürgermeisterin, die auch zuständig ist für das Forstamt und zusammen mit einem Forstamtsleiter auch auseinandergesetzt in der Diskussionsrunde und da habe ich dieses Konzept eingesehen. Und dieses Konzept finde ich aus fachwissenschaftlicher Sicht hervorragend erarbeitet, was also in meinen Augen auch den Spagat zwischen einer zukunftsfähigen Nutzbarkeit der Wälder und der Befriedigung auch der menschlichen Bedürfnisse nach erneuerbaren Ressourcen sehr schön abwägt mit verschiedenen Aspekten der Konservierung von

	Ökosystemen, auch diese Gratwanderung von Baumartenveränderungen im Klimawandel adäquat anspricht und verantwortungsvoll in meinen Augen damit umgeht. Sodass man also nicht in irgendwelche operative Hektik verfällt und jetzt riesige Flächenszenarien mit irgendwelchen abstrusen Baumarten anpflanzt, sondern dass man also abwägt, viele Aspekte hört und eine differenzierte Zukunftsperspektive aufzeigt. Das fand ich sehr ansprechend bei den Elementen, die ich da zumindest damals mir erschlossen hatte.
8	**LT:** Welche Konflikte sehen Sie zurzeit, wenn es die Klimawandelfolgen immer stärker werden und der gleichzeitigen Erhaltung der Qualität des Stadtwaldes in Karlsruhe?
9	**SP4:** Also ich glaube, da kann ich nicht ganz speziell für Karlsruhe sprechen, es sind eigentlich übergelagerte Konflikte, die so ziemlich alle Wälder angehen dürften. Einen kleinen Teilaspekt gibt es auch spezifisch für Karlsruhe sicherlich. Aber so die übergelagerten Konflikte sehe ich eher im Bereich Gesellschaft und gesellschaftlicher Diskurs, weil man sich so ein bisschen in der öffentlichen Diskussion die Frage stellen muss, was sind hier die prioritären Waldfunktionen? Und wir wissen nicht so genau, ob wir die sogenannte multifunktionale Waldbewirtschaftung wie wir sie heute eigentlich kennen, in der Zukunft auch noch so weiter fortschreiben können. Denn ein Bestandteil der multifunktionalen Waldbewirtschaftung ist definitiv auch, dass hier eine gewisse Ertragsfunktion wahrgenommen wird. Und diese Ertragsfunktion ist bestimmt in anderen Regionen Baden-Württembergs viel stärker ausgeprägt als im Karlsruher Stadtwald, aber an solchen Orten ist definitiv auch die Ertragsfunktion mit einem deutlichen Warnhinweis gekennzeichnet, wenn man an die Anpassungen an den Klimawandel denkt. Also unsere ertragsstarken Nadelbaumarten, ich will nicht sagen "sind angezählt", aber es ist zu befürchten, dass diese ertragsstarken Nadelbaumarten in Zukunft an Bedeutung verlieren könnten. Und das ist eine Diskussion wo man dann sagen muss, vielleicht ist dann gesamtgesellschaftlich mit der Fernsichtbrille auf das Ende des Jahrhunderts gerichtet, vielleicht auch eine Neujustierung in der Priorisierung und der Abwägung der verschiedenen Waldfunktionen angezeigt. Das wäre die allgemeine Aussage von mir und im speziellen Hinblick auf Karlsruhe ist natürlich die Dramaturgie noch eine deutlich verschärfte. Weil wir in Großteilen des Karlsruher Stadtwaldes natürlich einfach in einer klimatischen Region sind, die schon heute sehr, sehr stark zu kämpfen hat mit den Folgen des Klimawandels, weil wir einfach von der Meereshöhe schon in der Temperatursituation sind, die sehr, sehr angespannt ist. Und da verschärft sich natürlich einfach auch nochmal das Thema Mortalitätsrisiken von Waldbäumen auf der Individuenbasis aber auch von Waldbeständen auf der Landschaftsbasis, da ist natürlich auch der Waldverlust so ganz generell zu befürchten. In einigen Regionen hat das in den letzten Jahren ja auch schon begonnen oder in einigen Distrikten des Stadtwaldes, dass Auflöseerscheinungen von Waldbeständen da sind. Das heißt also ich glaube, dass einfach die Ertragsfunktion von Wäldern noch viel stärker marginalisiert werden muss, im Stadtwald, und dass die Erholungsfunktion, die Naturräume generell, die Erholungsfunktion auch für die Stadtbevölkerung viel, viel gewichtiger wird und dass auch die Schutzfunktion mit langer Sicht an Bedeutung gewinnen wird. Denn wir müssen ja auch einfach die Verpflichtung zur Biodiversitätssicherung und Biodiversitätserhaltung im Auge behalten, weil der Schutz der Biodiversität ja auch eine Maßnahme der allgemeinen Zukunftsvorsorge ist. Das wäre so das biotische Potenzial unserer belebten Umwelt erhalten müssen und darin sehe ich auch eine besondere Bedeutung, also Priorisierung, Veränderung, der Gewichtung der Waldfunktionen für Karlsruhe glaube ich.
10	**LT:** Dann legen wir mal den Fokus mehr auf diese Erholungsfunktion. Und zwar gibt es ja ganz viele Forschungen dazu, dass der Waldbesuch sehr gut für die psychische und physische Gesundheit der Menschen ist, gerade der Stadtbevölkerung und auch dass jetzt seit einem Jahr nach dem Ausbruch der Covid-19-Pandemie immer mehr Menschen in den Städten in den Wald gehen. Da wollte ich fragen, ob Sie da Konflikte sehen?
11	**SP4:** Würde ich mal so ganz pauschal verneinen, als Einstieg in die Antwort. Es ist nichts Neues, dass die Bevölkerung prinzipiell den Wald nutzt als Erholungsraum und da haben sich immer

	schon Veränderungen auch ergeben. Vor der Pandemie war es vielleicht der Singletrail vom Mountainbiken, das war die letzten 10-15 Jahren so ein bisschen ein Hype, weil es eine Veränderung der Erholungsnutzung war in den Wäldern. Und solche Veränderungen ergeben natürlich immer erstmal zunächst Anpassungsbedarf und man muss vielleicht auch Besucherlenkungssysteme verändern und anpassen an die sich verändernden Ansprüche. Insofern glaube ich also, ist es nicht im Konflikt, sondern ich glaube, dass das einfach ein Handlungsbedarf an eine Anpassung ausdrückt. Grundsätzlich ist es natürlich so, dass die Erholungssuchenden mit ernsteren Formen der Waldbewirtschaftung grundsätzlich einen Zielkonflikt haben. Und je mehr Menschen sich natürlich einer friedvollen, ruhigen, idyllischen Naturgenuss hingeben wollen, dann steigt natürlich auch das Konfliktpotenzial erstmal grundsätzlich mit wirtschaftlichen Aktivitäten im Wald, das ist ja ganz klar. Aber man kennt ja auch schon aus dieser Konfliktforschung, die auch nicht neu ist, die ganz einfachen Handlungsanweisungen. Das ist Kommunikation - und zwar nicht die Feuer-austretende-Kommunikation, sondern die proaktive, wohlwollende Kommunikation mit den entsprechenden nutzenden Gruppen. Das bedeutet, wenn man jetzt also eine Veränderung in der Erholungsnutzung durch Pandemie beobachtet, dann kann man eventuell durch Schilder, durch Lenkungen, vielleicht auch einfach durch Personen, die im Wald da sind und Ansprechpersonen sind, kann man da die allermeisten Konflikte auch entschärfen. Und das muss man dann so anpassen. Wenn eine Priorität auf der Erholungsnutzung da ist, gibt es ja auch schon lange, dann kann man mit *Rangern* und mit verschiedenen Freizeitangeboten, mit Waldpädagogik, kann man da natürlich auch lenkend drauf reagieren.
12	**LT:** In welchen Kriterien würden Sie die Qualität des Stadtwaldes messen?
13	**SP4:** Ich glaube da muss ich ausweichend mit einer Rückfrage antworten. Ob das jetzt so pauschal den Stadtwald als solches betreffend soll, oder ob es etwas zielgerichteter sein soll?
14	**LT:** Eigentlich schon für Karlsruhe, aber sonst die Zielsetzung könnten Sie sich auch selber überlegen.
15	**SP4:** Ja ich weiß nicht ob man es an der altbackenen Funktionentriologie von Wäldern aufrollen sollte, also Nutzfunktion, Schutzfunktion und Erholungsfunktion. Das wäre eine Gliederungsmöglichkeit, um die Qualität des Stadtwaldes zu ermitteln. Also wir haben ja in Wäldern schon seit langer Zeit verschiedene Zertifizierungssysteme, die halt die Güte der Waldbewirtschaftung, aber auch die Wertigkeit vor Naturschutzfachlichem Hintergrund ermitteln. Also die Punktung von Kriteriensystemen, da kann ich vielleicht einfach nur auf die Nachhaltigkeitszertifizierung verweisen, verfolgen. Und man könnte dann natürlich genauso auch bei der Erholungsfunktion, da gibt es ja hier [15:57.5?], Nachhaltigkeitskriterien, da sind natürlich auch soziale Nachhaltigkeitsaspekte mit inbegriffen, verweisen. Das wäre ein adäquates Verfahren um die Qualität ganz umfassend der Wälder und der Funktionserfüllung der Wälder zu quantifizieren. Und wenn es um neuerliche Änderungen geht, dann müsste man die Bereiche 'Anpassung an den Klimawandel' anhand von Kriterien überprüfen - also inwiefern waldbauliche Maßnahmen ergriffen werden, die die Wälder resilienter und angepasster und anpassungsfähiger in Hinblick auf den Klimawandel umsetzen. Dazu gibt es rudimentäre Ansätze aus der deutschen Anpassungsstrategie, die auf Landesebene konkretisiert wurde, durch das Klimaschutzgesetz in Baden-Württemberg, da gibt es also auch einige Kriterien und Indikatoren die dann Fortschritte der Anpassung an den Klimawandel anzeigen. Und im Bereich der Erholungsnutzung, da könnte man sicherlich noch weiter ausbauen um dann die soziale Nachhaltigkeit auch noch besser auszudrücken. Vor dem Hintergrund der Pandemie glaube ich haben sich die nutzenden Gruppen noch ein bisschen verändert und auch die nutzenden Ansprüche. Denn man kann ja eben nicht mehr mit der Schulklasse und in großen Wandergruppen in den Wald gehen, sondern das individuelle nutzende Verhalten ist ja aufgrund der AHA-Regeln auch im Wald zu verfolgen. Also da wäre glaube ich ein bisschen Bedarf fürs *Redesign* um da die Qualität des Stadtwaldes im Hinblick auf die Erholungsfunktion zu überprüfen.

16	**LT:** Und welche politischen Instrumente wären das dann genau? Oder auch bei der Klimaanpassung, für die Anwendung?
17	**SP4:** Also politische Instrumente wären natürlich, dass man sich im politischen Raum einigt darauf, welches diese Kriterienlisten sein sollen, welche für einen prioritär sind. Und dann müsste man sich dann dazu durchringen, dass man so eine Art Monitoringsystem, also mit periodischer Evaluierung umsetzt, wo die Daten natürlich auch verfügbar sein müssen, also da muss man dann natürlich auch mit Befragungen in Hinblick auf die Erholungsfunktion arbeiten können, mit repräsentativen Bevölkerungsumfragen, also wenn es um die Zufriedenheit der Erholungssuchenden mit der Qualität der Wälder geht, da muss man dann jedes Jahr, oder alle drei Jahre oder fünf Jahre, welche Touren man sich in der Politik dann eben aussucht, müsste man sich darauf einigen und dann müssten da die Finanzmittel zur Verfügung gestellt werden um solche Studien auch adäquat begleiten zu können, um zu gucken ob es da erfolgreich umgesetzt wurde. Wäre das ausreichend in Hinblick auf politische Instrumente?
18	**LT:** Ja. Eigentlich sind ja Klimawandelfolgen und so Situationen wie die Covid-19-Pandemie sehr globale Problematiken. Wäre es dann angebrachter, sowas in größerem Rahmen als auf der lokalen Ebene zu klären? Also auf EU- oder sogar globaler Ebene, oder wie würden Sie das sehen?
19	**SP4:** Ich glaube das ist wie mit den meisten Fragen, genauer zu gucken. Bei der Pandemie ist es so wie mit dem Klimawandel, wie Sie sagen, da gibt es gewisse Grundtendenzen natürlich immer auf den übergeordneten Raumschaften, die sich abzeichnen. Also es ist in Taiwanesischen und Indonesischen Wäldern in der direkten Umgebung größerer urbaner Räume nichts anderes, weil das eine sehr, sehr Individuen zentrierte Nutzungsform bei der Erholungsnutzung in Wäldern dann halt einfach ausschlaggebend sein wird. Insofern sind Fragestellungen oder auch politische Instrumente dann sehr sinnvoll, wenn sie das so auch in der globalen Dimension mitdenken. Allerdings gibt es dann natürlich immer die lokalen und regionalen Gegebenheiten: Erstens mal möchten sich natürlich Städte und Gemeinden damit schmücken, dass sie jeweils einen lokal gültiges, individuelles Konzept haben. Und da sollte man sich dann nur eben gut überlegen, was muss ich denn wirklich mit lokaler Tönung individuell ausformen? Sowohl in politischen Instrumenten, als auch bei den Anpassungsmaßnahmen. Was unterscheidet unseren Naturraum oder auch unseren gesellschaftlichen Raum hier vor Ort eindeutig von anderen vergleichbaren Kommunen? Also das müsste man sich aus handelnder Sicht natürlich sehr, sehr gut überlegen. Wenn man natürlich in Geld schwimmt, dann ist der Druck zu dieser Differenzierung etwas geringer und man kann es sich leisten, auch ein umfassendes Konzept zu erstellen, was natürlich auch die globalen Aspekte mit beinhaltet. Aber es gibt natürlich auch sehr viele klamme Kommunen, die dann sehr gut filtern müssen, was können wir denn überhaupt stemmen? Und das gilt glaub ich für den Klimawandel genauso. Beim Klimawandel hat man noch ein kleines bisschen den Unterschied, dass der Naturraum noch stärker mit reinkommt. Also wir wissen tatsächlich aus den Anspruchsanalysen der Erholungsnutzenden, dass die recht zufrieden sind, wenn es grüne Landschaften sind und wenn vielleicht auch mal interessante Landschaftsbestandteile wie ein Stein oder ein Felsen oder vielleicht auch mal ein See oder ein offener Ausblick vorhanden sind. Was stark stört in Wäldern für Erholungssuchende, das sind halt Einflüsse der Technosphäre, also wenn da ein *Harvester* steht, wenn tiefe Fahrspuren sind, wenn riesige Holzpolter da sind die eigentlich eher stören und nicht inspirierend sind. Solche Sachen, also abrupte Veränderungen, Einflüsse der Technosphäre - das stört. Und beim Klimawandel ist es ein bisschen anders, denn beim Klimawandel haben wir einen viel, viel stärkeren Bezug auch zur tatsächlichen Waldbestockung. Je nachdem welche Arten vorhanden sind und wie stark sich welche klimatischen Veränderungen in meiner Region wirklich auswirken, haben wir glaube ich ein bisschen stärkeren Bezug dazu auch lokale und regionale politische Instrumente und Anpassungsstrategien aufzusetzen.
20	**LT:** Was ist Ihre Meinung dazu, dass jetzt in Baden-Württemberg eine Reformierung gegeben hat? Also dass der Staatswald von der Anstalt des öffentlichen Rechts Forst BW verwaltet wird, jetzt

	seit 2020, und das Forstamt in Karlsruhe noch die Körperschaftswälder verwaltet?
21	**SP4:** In Hinblick auf welchen Aspekt?
22	**LT:** Z.B. die Ausführung und Anwendung von Klimaanpassungsstrategien?
23	**SP4:** Ja ich denke mal, dass es zu einer viel, viel größeren Heterogenität kommt, bei der Anwendung von Anpassungsstrategien. Und wir hatten natürlich die Situation vorher auch schon, dass wir ja eine Kommunalisierung hatten, der Forstverwaltung, 2005 oder sowas glaube ich war da der Stichtag. Die sogenannte "Teufereform", wo wir schon auf die Landkreisebene gegangen sind und wo wir so eine Zwitterstellung hatten. Es gab ja noch die Einheitsforstverwaltung, das heißt Gemeinschaftswälder und Staatswälder wurden von der gleichen Forstverwaltung betreut. Aber viele Mitglieder im Revierdienst waren ja kommunalisiert und damit natürlich auch schon der jeweiligen Kreisverwaltung unterstellt. Das heißt dadurch hatte sich schon eine größere Diversifizierung auch in den, ich sag mal "in den Befehlsstrukturen" ergeben und damit auch die Umsetzungsmechanismen. Aber das ist jetzt noch deutlicher vorgenommen worden, weil jetzt auch die Personalaspekte halt eindeutig getrennt sind. Weil jetzt eben die Revierleitenden nur noch Staatswald oder nur noch Gemeinschaftswald betreuen. Und das heißt es wird sich weiter fortsetzen, dass also die Umsetzungskonzepte viel stärker variieren werden zwischen den verschiedenen Kommunen, den verschiedenen Gemeinschaftswäldern. Davon würde ich persönlich ausgehen und die Anpassungen in den Staatswäldern, die Sie ja wahrscheinlich gar nicht bei Ihrem Interview primär verfolgen, die dürfte tendenziell natürlich durch eine straffere Organisationsform tendenziell einheitlicher erfolgen, wobei nach wie vor man davon ausgehen wird, dass auch dort die unterschiedliche Gewichtung der Waldfunktionen bei den unterschiedlichen Revieren auch bestehen bleiben dürfte. Also das auch Staatswaldreviere im urbanen Umfeld eine andere Funktionenpriorisierung erfahren als sehr ländlich gelegene Staatswaldreviere mit Fichtenforsten, sage ich jetzt mal, wo die Ertragsfunktion ganz weit oben steht.
24	**LT:** Okay, das war es eigentlich schon. Ich würde zum Schluss nochmal fragen ob Ihnen noch irgendetwas einfällt oder ob Sie zu irgendeinem Thema noch was sagen wollen, was wir angesprochen hatten?
25	**SP4:** Nö ich habe eigentlich das Gefühl, dass ich so das zum Besten geben konnte was in meinem Kopf aktuell, ohne dass ich mich länger vorbereitet habe, herumspukt. Ich glaube, dass wir über ein Projekt-Verbund ja wahrscheinlich auch die Möglichkeit haben, dann von den Ergebnissen Ihrer Masterarbeit zumindest im stenographischen Stil zu erfahren, eventuell, ich weiß nicht ob das bei einer Kollegin auftauchen wird oder wo Sie zugeordnet sind? Sie sind an einem Institut glaube ich, oder?
26	**LT:** Genau. Also die ist ja auch im Projekt.
27	**SP4:** Also oder anders gesagt, mich würde es dann interessieren, vielleicht ein Ergebnis oder eine Kurzzusammenfassung Ihrer Arbeit auch Informationshalber zu erhalten. Sie werden ja mehrere Interviews führen und was Sie dann im Rahmen Ihrer Synthese halt auch herausdestillieren können aus den verschiedenen Meinungen, die Sie jetzt hören. Weil es bestimmt auch für mich interessant und auch relevant ist, wir betrachten ja tatsächlich in unserem Teilvorhaben eher die Stadtbäume und nicht den Stadtwald, insofern wäre das für mich dann interessehalber eine Ergänzung.

SP5

1	**SP5 (30:17.6 Minuten)**
2	**LT:** Inwiefern haben Sie denn durch Ihre Arbeit einen Bezug zum Stadtwald?

3	**SP5:** Also zum Stadtwald haben wir in meiner Arbeitsstelle insofern Bezug, dass wir uns eigentlich so permanent mit der Frage beschäftigen, wie muss der urbane Raum gestaltet sein? Oder erstmal auf der Analyseebene vielleicht auch erstmal in der Bestandsaufnahme, wie ist der urbane Raum gestaltet? Und ist das eigentlich gesundheitsförderlich? Ja, also trägt das zur Gesundheitsförderung bei oder eben, wenn nicht, wie könnte urbaner Raum gestaltet sein? Und da spielt Stadtnatur und damit natürlich auch der Stadtwald natürlich eine erhebliche Rolle.
4	**LT:** Und im Bezug zum Klimawandel, sehen Sie da einen Zusammenhang?
5	**SP5:** Zum Stadtwald oder zur Gesundheit? Oder beides?
6	**LT:** Beides.
7	**SP5:** Ja auf jeden Fall. Also für die Klimaproblematik ist es natürlich. Also ich werde das vielleicht so -als kurzen Exkurs- bevor ich an meiner jetzigen Arbeitsstelle angefangen habe, war ich an einer anderen Arbeitsstellt. Ich weiß nicht, ob Ihnen das was sagt, die sich vor allen Dingen auch viel, also ja weniger mit dem Gesundheitsaspekt, sondern viel stärker mit diesem Klimaaspekt auseinandersetzten, so aus der Nachhatligkeitsrichtung, aus der und der ökologischen Richtung. Und dieser Wechsel an die jetzige Arbeitsstelle hat dann natürlich für mich so quasi so eine neue Perspektive eröffnet, die auch meine vorherige Argumentationsstruktur, also wenn man sich vorher versucht hat irgendwie für einen klimawandelgerechten Umbau der Gesellschaft und der Stadt einzusetzen, dann bietet natürlich diese Gesundheitsperspektive auch einen neuen Argumentationspunkt, an den man ansetzen kann, weil dann natürlich unglaublich viele *Co-Benefits* sind, die man da abrufen kann, weil man aus Gesundheitsperspektive natürlich weiß, dass Stadtgrün und vor allen Dingen auch Bäume und dadurch auch Stadtwald, aber auch jenseits des Stadtwaldes auch Stadtbäume in einer Wohnstraße z.B. unglaubliche Gesundheitsvorteile mit sich bringen. Das fängt an mit der verbesserten Luft, das fängt an, dass Stadtwald Bewegung fördert und natürlich dadurch eine Aktivität irgendwie auch nach vorne bringt. Das sind aber auch Dinge wie mentale Gesundheit, Stressabbau. Der Wald hat unglaubliche Wirkung auf unsere mentale Gesundheit und wirkt sich da positiv aus. Aber auch Herz-Kreislauf-Dinge z.B. Es ist relativ erwiesen, dass Leute mit Herz-Kreislauf-Problemen, dass sich das sehr positiv auswirkt, wenn die sich recht regelmäßig im Wald aufhalten oder wenn die sich regelmäßig zumindest in Gegenden aufhalten, wo Bäume sind. Spielt vielleicht auch in gewisser Weise die mentale Gesundheit eine Rolle, so, das sage ich jetzt mal so ein bisschen thesenhaft, weil ich bin auch kein Arzt, bis zuletzt ist da der Zusammenhang noch nicht belegt, aber ich halte das für sehr wahrscheinlich. Die Japaner z.B. haben sehr sich intensiv auseinandergesetzt mit den psychologischen Effekten und da diesen Begriff des *forest bathing,* also dieses im Wald baden, was das für psychologische Vorteile mit sich bringt. Und insofern ist das natürlich, gibt es da ganz viele Schnittmengen in der Argumentation zu sagen, lasst uns die Städte begrünen, um da auch aus Klimaperspektive den Temperaturanstieg runterzubringen, den CO_2-Anstieg runterzubringen, weil Bäume und Pflanzen das natürlich unglaublich kompensieren können und so. Und aus der Gesundheitsperspektive kann man dann natürlich auch sehr, auch mit den urbanen Hitzeinseln, das ist jetzt weniger Ihr Stadtwaldthema, sondern aber das urbane Thema, die urbanen Hitzeinseln, die natürlich durch diese Stadtbegrünung aufgehoben werden können. Um dann wieder beim Stadtwald zu sein, wenn der vernünftig in der Stadtstruktur angelegt ist, also da muss man dann auch sehr genau gucken mit den Gebäuden und sowas, wie man das so anordnet mit dem Stadtbild, dass der dadurch dann natürlich auch Kaltluftschneisen durch die Stadt befördern kann, was dann wieder die Hitze runterbringt. Dadurch haben wir dann natürlich auch weniger Probleme mit, das sind vor allem dann auch alte Leute, die davon betroffen sind, von diesen sommerlichen Hitzetemperaturen und deren Kreislaufproblemen, die da alle herrühren oder Dehydrierung und was da so alles dazugehört. So also da gibt es schon massive Schnittmengen natürlich.
8	**LT:** Wo sehen Sie jetzt also, der Klimawandel bedroht ja diese Funktionen. Wo sehen Sie da Lösungsansätze auf politischer Ebene dem entgegen zu wirken?
9	**SP5:** Es müsste politische eine Entscheidung dafür getroffen werden mehr Stadtgrün zu etablieren, also das wäre der Punkt, denke ich. Und da ist natürlich immer, also das ist natürlich immer ein vielschichtiges Problem ist jetzt, ich möchte das Wort "Problem" gerne vermieden, weil sonst wird

	das immer gleich so aufgebauscht, aber da sind natürlich, da hängen natürlich Dinge dran, die man sich vorher dann fragen muss. Das fängt an mit, welche Pflanzen nehmen wir überhaupt? Das betrifft auch den Stadtwald, wenn wir davon ausgehen, dass es wohlmöglich wirklich klimatische Veränderungen bereit, dass sie bereits einsetzten oder dass wir sie nicht mehr vermeiden können. Und wie es im Moment aussieht ist das sehr wahrscheinlich, weil wir sind von unserem 1,5 Grad Ziel auch noch Lichtjahre entfernt, der allgemeine Trend ist eher bei 3 bis 4 Grad globaler Erwärmung. Und da muss man sich natürlich Fragen stellen, welche Bäume pflanze ich in so einem Stadtwald überhaupt? Welche sind an diese neuen Bedingungen angepasst oder können damit umgehen? Aus Gesundheitsperspektive dann natürlich auch wieder in der Abwägung. Ich halte die Konflikte dafür sehr handhabbar, aber natürlich gibt es da Konflikte, das ist das Stichwort: Pollenallergiker. Solche Dinge natürlich, wo man, wenn ich einen halben, einen ganzen Stadtwald mit Haselnusssträuchern vollpflanze, dann wird es z.B. das ist ja so ein klassisches Allergiker-Gewächs, das könnte wohlmöglich ein Problem werden, aber das kann man, denke ich, lösen. Das ist keine Magie, da muss man dann einfach auch ein bisschen miteinander sprechen und dann auch die Botaniker zurate ziehen, was gibt es da für Pflanzen, was gibt es da für Möglichkeiten? Dass die Fichte vielleicht auch einfach ausgedient hat, jetzt wenn wir noch die Borkenkäferproblematik haben. Ist dann auch noch so eine Sache. Die ist auch trockenresistent, nicht wahnsinnig gut, wenn wir uns die letzten zwei, drei heißen Sommer anschauen. Also das sind natürlich Dinge, an die man sich anpassen muss, über die man sprechen muss, aber die aus meiner Sicht lösbar sind. Zweiter Punkt ist natürlich dann immer das Todschlagargument der Finanzierung. So was so oft von politischer Seite kommt, wer soll den ganzen Kram bezahlen? Ja natürlich muss man sich darüber Gedanken machen, aber ich würde jetzt auch mal sagen, da ist die Politik eben auch für zuständig, ich meine dafür haben die diesen Posten, um sich darüber Gedanken zu machen. Es gibt einen gewissen Haushalt, den kann man vielleicht auch mal etwas umstrukturieren. Vielleicht muss man auch über Dinge sprechen, wo viel Geld ausgegeben wird, sich zu fragen, muss das sein? Da kann man auch sich dann mal mit der Verkehrsperspektive auseinandersetzten. Also aus Gesundheitssicht würde man natürlich auch dafür plädieren, weniger Autoverkehr in den Innenstädten. Die die Schadstoffbelastung in der Luft runterbringen würde, was mehr Platz in den Innenstädten schaffen würde, wodurch die Leute mehr Platz hätten sich da anderweitig zu bewegen. Wir bräuchten mehr alternative Fortbewegungsmittel, wie z.B. das Fahrrad oder eben auch mehr zu Fuß gehen, also Stichwort fußgängerfreundliche und fahrradfreundliche Städte. Die haben den Klimaeffekt, die haben aber auch natürlich den Gesundheitseffekt. Wenn wir und anschauen, dass die Zahl derjenigen, die immer mehr unter, zum einen unter Übergewicht leiden, aber eben auch unter diesen Herz-Kreislauf-Erkrankungen diese klassischen. Was oft einfach ein Produkt von mangelnder Bewegung ist. So und da kann man dann natürlich auch wieder Synergien schöpfen und da wäre dann auch z.B. dann auch erneuter Platz für mehr Stadtgrün. Also solche Konzepte kann man natürlich denken. Und dieses Argument von, das ist alles nicht zu bezahlen, finde ich bisweilen auch manchmal etwas, andere Sachen sind auch zu bezahlen. Das muss man klug überlegen und das muss man Klug rechnen, aber da sehe ich eine Politik auch in der Pflicht, weil das ist ihr Job.
10	**LT:** Jetzt haben wir schon über die gesundheitliche Wirkung von Stadtwäldern geredet. Und diese Funktion oder auch die Erholungsfunktion wurde jetzt, seit dem Ausbruch der Covid-19-Pandemie, immer mehr genutzt und viel mehr Menschen, also gerade die Stadtbevölkerung, geht in den Wald. Sehen Sie da Konflikte?
11	**SP5:** Konflikte zwischen Gesundheit und Klima? Also den zwei Fraktionen? Oder zur Politik?
12	**LT:** Zur Politik, aber auch einfach, dass jetzt mehr Menschen im Wald sind.
13	**SP5:** Also aus Corona-Perspektive. Man könnte natürlich auf Naturschutzperspektive argumentieren, wenn die Wälder so voll sind, gibt es natürlich vielleicht Konflikte mit der Flora und Fauna vor Ort. Je mehr Leute, um es jetzt mal so flappsig zu sagen, je mehr Leute dadurch trampeln, geht das an der Vegetation dann natürlich nicht spurlos vorüber. Sollte es wirklich auch eine solide Zahl an Wildtieren in dem Wald geben, gerade jetzt der Frühling kommt in der Frühjahrssaison: dann sind da Rehe und Kidse, wobei die sind einfach eher in den Wiesen, weniger in den Wäldern.

	Aber auch Wildschweinrotten, die jetzt anfangen Frischlinge zu kriegen und sowas, die sich dann von vielen Leuten oder einer erhöhten Anzahl an Menschen bedroht fühlen können sicherlich. Und jeder, der mal gesehen hat. wie so eine Wildsau reagieren kann, wenn sie ihre Jungen bedroht sich, weiß, dass das auch für den Menschen bedrohlich werden kann. Da gibt es, ja, diese Konflikte gibt es da sicherlich. Aber die Frage ist jetzt, was ist die Alternative? Also den Wald absperren, dass da keiner mehr rein kann, halte ich jetzt für keine gute Alternative. Sondern da muss man vielleicht auch eher gucken, haben wir auch ausreichend Ausweichmöglichkeiten? Man muss auch so eine Stadt mal analysieren und schauen, wo haben eigentlich die Leute Zugang zum Wald? Und ist das eigentlich gleichmäßig verteilt, ist das eigentlich auch gerecht verteilt? Weil wenn man sich das manchmal detailliert anschaut, dann stellt man fest, dass viele Städte oder einige Städte zumindest auf ihre Stadtfläche gerechnet natürlich damit auch punkten, dass sie argumentieren, wir haben unheimlich viel Waldfläche auf unserem Stadtgebiet und auf viele Städte trifft das auch zu. Und dann das auch runterrechnen auf das betrifft dann auf die Bevölkerung gerechnet hat die so und so viel, hat jeder einzelne so und so viel Platz im Stadtwald, wenn man sich das dann aber auch mal auf Stadtviertel- oder Quatiersebene anschaut, stellt man oftmals fest: es gibt gewisse Stadtteile, die haben sehr leichten Zugang zum Wald, so quasi um die Ecke und dann gibt es Stadtteile: bis die im nächsten Wald sind, sind die eine dreiviertel Stunde unterwegs mit Bus und Bahn am besten noch. So das hat dann auch aus gesundheits-, also Gesundheitsgerechtigkeit ist das schwierig. Und das sehen wir in ganz vielen Dingen, was die Gesundheitsperspektive betrifft. Das es da natürlich unglaubliche Ungerechtigkeit gibt, weil das einfach räumlich ungerecht verteilt ist.
14	**LT:** Wie könnte diese, ich habe es unter Umweltgerechtigkeit oder -ungerechtigkeit, jetzt auch bei steigender Urbanisierung z.B. gelöst werden?
15	**SP5:** Man muss natürlich, es gibt da auch noch gewisse offene Fragen, die muss man dann vielleicht auch vorher, gut in Gänze kann man sowas selten lösen, aber dass wir mal eine Vorstellung davon kriegen, was sind auch Anforderungen an Stadtgrün? Also wenn wir uns jetzt wirklich mal Stadtviertel angucken, die wenig Grün haben. Oder vielleicht auch das Grün, was sie haben ist dann vielleicht auch in einem schlechten Zustand oder es wird auch einfach nicht genutzt, vielleicht ist es in einem guten Zustand. Vielleicht haben wir in einem Stadtteil, da ist auch eine Parkanlage, die ist auch eigentlich super gepflegt und alles, aber sie wird nicht genutzt. Da muss man sich die Frage stellen, woran liegt das? Und dann muss man auch mal gucken, welche Anforderungen muss so ein Stadtgrün vielleicht auch an die örtliche Bevölkerung erfüllen? Sind es da vielleicht, ist der vielleicht nicht barrierefrei zugänglich und leben in dem Stadtteil vielleicht vor allen Dingen alte Leute, die viel mit Rollatoren und anderen Gehhilfen unterwegs sind? Da muss man dann vielleicht anpassen. Oder ist der irgendwie so angelegt, dass Leute abends im Dunkeln da vielleicht auch Angst haben dadurch zu gehen? Ist da vielleicht ein Unsicherheitsaspekt, muss man da vielleicht eine Beleuchtung installieren? Oder sind da in dem Stadtteil vielleicht irgendwie verschieden Kulturen untergebracht, die auch unterschiedliche Vorstellungen haben, wie so etwas auszusehen hat oder wie das aussehen muss, dass man das nutzt und so. Also solche Fragen muss man dann im Vorhinein und da ist Forschungslage sehr schlecht muss man sagen. Das könnte man dann einfach mal, das muss man dann wohl noch ein bisschen beforschen, um das rauszufinden, aber ja. Ansonsten besteht wirklich die Lösung in dem -dass meine ich, man muss da schauen die Umweltgerechtigkeit, das betrifft oft Stadtteile, die sind nicht nur in Sachen Umweltgerechtigkeit schlechter gestellt, sondern in vielen anderen Gerechtigkeitsaspekten auch schlechter gestellt. Und da muss man dann vielleicht auch manchmal etwas grundsätzlich dran gehen. Und das sind natürlich Sachen, da macht man dann teilweise ein so großes Fass auf, dass die Politik da zurückschreckt. Also wir haben jetzt z.B. über in meinem Wohnort bei und vor Ort ist im Moment eine Problematik, dass im einen Stadtteil meines Wohnortes wurden, also es hat sich entzündet, dass zwei Krankenhäuser geschlossen worden sind und wenn man sich das jetzt anschaut, was die Bettenverfügbarkeit im Stadtraum meines Wohnortes betrifft und auch die Entfernung mit, man ist innerhalb von 10 Minuten mit dem Krankenwagen im nächsten Krankenhaus und sowas: war es völlig legitim diese Krankenhäuser zu schließen und die waren auch nicht ausreichend ausgelastet. Aber natürlich hat das für wahnsinnige Empörung gesorgt, weil die eben in einem Stadtteil meines Wohnortes

	angesiedelt sind und der eine Stadtteil meines Wohnortes gilt sowieso als benachteiligter Stadtteil. Es gibt sogar viele Stadtteile. Aber in meinem Wohnort hat man ein sehr starkes soziales Gefälle zwischen der dem einen und dem anderen Stadtteil. Und da hat das natürlich sehr große Empörung hervorgerufen, weil die sowieso in vielen anderen Dingen auch immer schon irgendwie da ein Ungerechtigkeitsgefühl haben, berechtigter Weise. Und da muss man jetzt vielleicht auch Lösungen finden, die da grundsätzlich auch mal ein bisschen rangehen. Vielleicht auch mal stadtplanerisch da mal ein bisschen das versuchen aufzubrechen.
16	**LT:** Und ziehen Sie aus der Covid-19-Pandemie jetzt Konsequenzen für die Verwaltung des Stadtwaldes für die Zukunft?
17	**SP5:** Für die Verwaltung des Stadtwaldes? Eigentlich eher für die Verwaltung der allgemeinen Stadtplanung. Wenn ich das mal so sagen darf. Weil, wie Sie ja schon richtig gesagt haben in der Corona-Pandemie fingen auf einmal an die Stadtwälder und die Parks zu füllen, während die Innenstädte völlig verwaist waren. So kann man, und das war für mich eigentlich eher ein Indikator, dass man dann auf einmal sehr plastisch gesehen hat, dass diese Innenstädte, in denen sehr viele unserer Städte zu nichts anderes dienen als zum Konsum. Sobald die Konsummöglichkeit nicht mehr gegeben ist, sprich dann in der Corona-Pandemie: die Geschäfte mussten schließen, die Gastronomie musste schließen, haben diese Innenstädte offensichtlich keinerlei Aufenthaltsqualität. Niemand möchte sich dort aufhalten, weil die offensichtlich auch dermaßen uneinladend gestaltet sind, dass man sich dann auch eben in den Wald und in den Park flüchtet. Und da wäre für mich eher die Konsequenz, dass die Stadtplanung und eine allgemeine Stadtverwaltung sich mit diesen Innenstädten auseinandersetzen muss. Um da vielleicht auch Alternativen zu schaffen. Das würde auch die Gefahr, darauf wollten Sie vielleicht auch vorhin hinaus, die Gefahr des übervölkerten Waldes etwas entgegenwirken, dass es sich etwas entzerrt.
18	**LT:** Dann habe ich noch eine etwas allgemeinere Frage. Und zwar in welche Qualitäten würden Sie den Stadtwald messen?
19	**SP5:** So als Messindikatoren quasi? Der hätte natürlich die Aufenthaltsqualität, die gesundheitlichen Faktoren natürlich im Sinne von Luftverbesserung und aber eben auf der anderen Seiten gleichzeitig auch mit biologische Vielfalt, Artenvielfalt, Pflanzenvielfalt. Und dahin gehend natürlich dann auch die Frage, wie groß ist der Klimaeffekt? Quasi den er leisten kann. Ich meine, da sind ja dann auch nicht alle Pflanzen gleich, ich bin kein Botaniker auch ich muss da mit den entsprechenden Experten sprechen, aber die Gesundheitsleistungen sind davon ja auch abhängig. Und auf Stadtwald dann auch bezogen zu gucken, wie ist der geographisch angesiedelt? Das sind dann einmal die Umweltgerechtigkeitsfragen, aber eben auch die Klimafrage im Sinne von, fügt der sich in das gesamte Stadtbild ein, um diese z.B. die Kaltluftschneisen und sowas zu fördern? Das sind, denke ich so, die Indikatoren. Lädt er ein zu einem, ja die Aufenthaltsqualität hatte ich ja gesagt und dann eben in Kombination mit einem aktiven Leben dort auch, also quasi fördert das körperliche Aktivität? Aus Gesundheitsperspektive, für die mentale Gesundheit. Diese Aspekte. Ist es vielleicht auch eine Kombination mit Stadtgrün und Stadtblau? Gibt es in dem Wald vielleicht noch einen schönen See oder einen Teich? Was auch gesundheitlich Aspekte hat und Klimaaspekte.
20	**LT:** Und jetzt sind ja der Klimawandel und die Covid-19-Pandemie sehr globale Probleme. Und finden Sie, dass die am besten auf lokalster Ebene lösbar sind, also jetzt auch im Hinblick auf die Stadtwaldverwaltung, oder sollte es eher auf nationaler, internationaler Ebene geregelt sein?
21	**SP5:** Das kann man eigentlich nicht weder dem einen, noch dem andern zuschieben, wenn man mal ehrlich ist. Also, es wird gerne versucht, dass irgendwie. Ja die einen sind von der Fraktion, das muss global gelöst werden, die anderen sind von der Fraktion, das muss alles auf die lokale Ebene. Ich denke, dass ist, die müssen da sich schon gegenseitig, die müssen da schon zusammenarbeiten. Das kann man nicht dem einen, dem anderen, den Schwarzen Peter zuschieben, um das jetzt mal so zu sagen. Natürlich sind Aspekte des Klimaschutzes eigentlich eher, kann man fast besser auf einer globalen oder zumindest auf einer größeren Ebene als der rein lokalen regeln, weil da geht es ja im Prinzip in Anführungszeichen, nur um die Reduzierung der CO_2-Emission. Wo ich die reduziere ist erstmal unerheblich, weil die Durchmischung in der Atmosphäre um den Planeten, also das muss einfach insgesamt runter. Wo ich das mache ist egal. So vereinfacht gesagt. Wenn wir

	jetzt über die Aspekte der Klimaanpassung sprechen. Das ist natürlich eine sehr lokale Frage. Weil sehr lokal unterschiedliche Betroffenheit vorherrschen. Und das muss man dann natürlich auch sehr lokal, also einmal was die klimatischen Veränderungen betreffen ist das lokal sehr unterschiedlich, aber eben auch ein Starkregenereignis z.B. kann sich an der einen Stelle ganz anders auswirken, als an der anderen Stelle, weil es vielleicht auch stadtplanerisch ganz anderes angelegt ist und sowas. Und eben die angesprochenen Gerechtigkeitsaspekte sind auch überall vielleicht manchmal auch etwas sehr spezifisch, dass man sich die speziell anschauen muss. So, die müssen natürlich dann vielleicht auch lokal angegangen werden. Eine allgemeine globale oder nationale Strategie dazu verabschieden, die vielleicht auf den lokalen Fall nicht passt oder nur sehr schwer anwendbar ist, ist nicht sinnvoll. Aber da müsste eine Verknüpfung der verschiedenen Ebenen stattfinden. Wäre meine Meinung, dass das viel stärker miteinander Verknüpft werden muss und dann wirklich klug überlegt werden muss, was mache ich lokal und was mache ich auf der höheren Ebene? Also das viel gelobte Subsidiaritätsprinzip und sowas, wie es auch auf der Europäischen Union immer proklamiert wird, halte ich durchaus für sinnvoll, Probleme da zu lösen, wo sie auftreten, allerdings muss man aufpassen, dass man nicht Gefahr läuft einfach immer alles von Oben nach Unten durchzureichen und dann stehen die Kommunen am Ende da und sollen es irgendwie lösen und deren Problem ist oft, dass sie weder die, also dass sich eigentlich schon keine finanziellen Ressourcen haben, um das zu machen. Und natürlich der Effekt manchmal fragwürdig ist, wenn sie sagen, wir machen hier vor Ort jetzt irgendwas, aber was der Effekt ist so minimal, weil es auf einer so kleinen Skala ist oder auf einem so kleinen Maßstab, dass es natürlich verschwindet. Und da müsste es eigentlich auf die höhere Ebene gebracht werden. So deswegen würde ich mich da ungern für eine Seite entscheiden.
22	**LT:** Alles klar. Letzte Jahr hat es in Baden-Württemberg nach einem Holzkartellverfahren eine Reformierung der Forstverwaltung gegeben. Und zwar wird jetzt der Staatswald von der Anstalt des öffentlichen Rechts ForstBW verwaltet und der Stadtwald, der Körperschaftswald in Karlsruhe vom Forstamt. Und so ganz allgemein, was ist Ihre Meinung zu solchen Reformierungen?
23	**SP5:** Da sind Sie jetzt recht tief drin im Forstrecht quasi, wo ich jetzt ganz offen zugeben muss, da habe ich mich bis jetzt noch nicht wahnsinnig viel mit beschäftigt.
24	**LT:** Ja das war jetzt ein sehr spezifisches Beispiel, aber vielleicht können Sie es auf die Stadtverwaltung generell projizieren?
25	**SP5:** Also eher so die Frage: sollte das in den Händen öffentlicher Verwaltung liegen oder sollte man das Privatisieren den Wald? Da, also in die Richtung denken Sie da oder was?
26	**LT:** Z.B. oder dass solche Veränderungen einfach auch die politischen Handlungen so aufhalten oder Prozesse wie Lösungen finden für den Klimawandel dadurch verlangsamt werden.
27	**SP5:** Also das kann mit Sicherheit passieren. Ich meine, jede Art von Verwaltungsakt hat ein Potenzial Prozesse einfach zu verlangsamen. Ich meine, gerade Deutschland ist bekannt für sein riesigen Verwaltungsapparat und so. Was auf der anderen Seite auch gut ist, also es kann passieren was will, die Verwaltung funktioniert immer irgendwie, aber sie funktioniert natürlich bisweilen sehr langsam und manchmal muss man sich auch fragen, sind das sinnvolle Prozesse, die da stattfinden? Forstrecht oder Forstverwaltung ist jetzt wirklich alles andere als mein Steckenpferd. Insofern würde ich mich jetzt ein bisschen auf eine allgemeine Antwort berufen im Sinne von, ja, natürlich kann das alles ein Problem sein und das kann auch nicht sinnvoll sein. Muss es aber jetzt auch nicht zwangsläufig. Bzw. im Zweifel kann alles immer ein Problem sein. Also wenn man es schlecht macht, kann alles zum Problem werden. Aber um jetzt z.B. die Frage, sollte man Wald stärker privatisieren? oder sollte es ein öffentliches. Nein, ich finde schon, dass es ein öffentliches Gut, ja wobei Privatisieren es ist ja relativ. Da müssten Sie vielleicht auch einen Juristen noch zu befragen, weil z.B. auch landwirtschaftliche Flächen sind natürlich irgendwie Privatbesitz im Sinne, dass es dem Landwirt gehört, aber natürlich hat der da auch eine Pflicht der öffentlichen Zugänglichkeit. Also kein Landwirt kann ja seine gesamten Ländereien hermetisch abschließen und sagen, ich lasse da die Öffentlichkeit nicht mehr rein. Sondern der muss da ja immer ein allgemeines Nutzungsrecht auch irgendwie gewährleisten. Deswegen wäre jetzt vielleicht eine Privatisierung nicht zwingend ein Problem. Es kann Möglichkeiten eröffnen, wenn da jemand ein Investor

	reingeht, der richtig auch die finanziellen Ressourcen hat, um auch das Nötige einfließen zu lassen, kann das vielleicht auch gut sein. Andererseits haben die letzten zwei bis drei Jahrzehnte der allgemeinen Liberalisierung von allen möglichen Dingen in unserem Wirtschaftssystem sich meistens nicht gerade als so richtig gut herausgestellt. Also wenn man es jetzt mal so ein bisschen Kapitalismus kritisch sieht. Muss man halt sehr vorsichtig sein. Insofern würde ich wahrscheinlich im Zweifel immer dazu tendieren, zu sagen, nein, das bleibt in öffentlicher Hand. Aber ja, die Stadtverwaltung und auch die Forstverwaltung sollte sich da jetzt nicht in irgendeinem Bürokratismus ergehen. Es ist ein schmaler Grat irgendwo dazwischen.
28	**LT:** Dankeschön. Dann sind wir auch schon beim Abschluss. Gibt es noch irgendetwas, was Ihnen im Zusammenhang zum Thema eingefallen ist, was Sie noch loswerden wollten?
29	**SP5:** Also nichts, was ich nicht wahrscheinlich schon gesagt habe. Ich plädieren vehement dafür, lasst diese Städte mehr begrünen und gerade Corona hat gezeigt, die Gestaltung unserer Innenstädte ist einfach eine Katastrophe, weil wenn es nichts anderes kann außer Konsum, dann ist das für mich unmöglich. Und da wäre für mich, grüne Alternativen, Stichwort Bäume, Stadtwald, und so was, immer das Mittel der Wahl und ansonsten allgemein durchaus auch die Einladung an diejenigen, die sich da aus Klimasicht sich für den Stadtwald einsetzen, die *Co-Benefits* für die Gesundheit miteinzubeziehen, weil das einfach auch eine starkes Argument bring, im Sinne von ich glaube, man kann viele Leute auch darüber erreichen, weil es sie sehr persönlich betrifft. Also wenn man den Leuten sagt, es betrifft deine persönliche Gesundheit. Oder eben auch auf der politischen Ebene, wenn man eben argumentiert mit es betrifft die konkrete Gesundheit der Bevölkerung, ist das bei manchen ein stärkeres Argument, als nur das Argument von hier sterben Bienen oder hier wird es einfach immer heißer, weil da oft dann das Gegenargument kommt, da werden wir uns schon irgendeine technische Lösung für einfallen lassen. Wir haben und bisher in den letzten 200 Jahren immer eine technische Lösung für alles einfallen lassen. Und das wird uns auch in diesem Fall gelingen. Bei der Gesundheit hört für viele quasi der, hört für viele der Spaß auf, um das mal so zu sagen.

SP6

1	**SP6 (21:48.3)**
2	[Dialog von 00:00.0-01:22.3 nicht transkribiert, um Anonymität zu wahren]
3	**LT:** Was bedeutet für Sie Naturschutz?
4	**SP6:** Naturschutz, das ist eine große Frage. Naturschutz.
5	**LT:** Bezogen auf den Wald.
6	**SP6:** Bezogen auf den Wald. Ja es ist deshalb eine große Frage, finde ich, weil sich Naturschutz auch verändert hat. Also vom Artenschutz, von etwas sehr, was ich, ja da habe ich auch dran Anteil gehabt, als Privatperson, so Orchideenschutz, Wiesen mähen und so weiter und man will dann genau diese Art da und da erhalten in so einem Prozessschutz. Und für mich ist Naturschutz eigentlich beides. Dass man Verantwortung übernimmt für gewisse Arten, die in unseren Regionen typisch sind, die diese Region als Verbreitungsgebiet brauchen und den auch diesen Raum zu lassen und aber auch so eine Prozesshaftigkeit zuzulassen. Und ich kann z.B. mit dem Naturschutz, der komplett Neophyten abwehrt, kann ich nicht durchgängig mitvertreten. Weil ich eben denke, Natur wandelt sich und diese Naturwandlung zuzulassen ist für mich auch ein aktueller Naturschutz.
7	**LT:** Die Stadt Karlsruhe hat ein Konzept zur Klimaanpassung für den Stadtwald veröffentlich letztes Jahr und auch wenn Sie das jetzt vielleicht nicht kennen, können Sie sich da Konflikte vorstellen?
8	**SP6:** Ja schon. Also Konflikte, die in diesem Konzept auftreten oder die in der Bewirtschaftung vorkommen. Also bei Stadtwäldern finde ich, generell, also ich meine, dass eine ist die Bewirtschaftungsfrage, wie richtet man den Wald aus? Bei Stadtwäldern erlebe ich das so, dass da schon ein Bewusstsein dafür da ist, dass der vielleicht weniger für die Holzproduktion da ist als andere Wälder. Und mehr für Erholungsnutzung, also da finde ich gehen Städte auch schon

	bewusster damit um, und versuchen das dann auch entsprechend zu schaffen. Natürlich sehe ich, was ich an Konflikten sehe ist generell, dass -ich nehme jetzt meinen Arbeitsort, wo ich das miterlebt habe- wo es erstmal eine Bürgerinitiative gibt, die eine ganz andere Art von Bewirtschaftung sich vorstellt, als der entsprechende vom Gemeinderat beauftragte Förster. Also wo dann die Nutzung gegen Schutz und Erholung letztlich stehen. Und ich glaube, dass das also neben diesen ganzen praktischen Fragen, welche Baumarten wollen wir fördern? Dass es ein Mischwald ist? Dass das Arten sind, die dem Klimawandel angemessen sind? Das ist für eine Stadt glaube ich, sehr wichtig die eigene Bevölkerung mitzunehmen und deren Erholungsbedürfnisse mitzudenken auch mal mit zu kartieren, wo gehen denn die Leute eigentlich am meisten hin? Auch Konflikte, Sie haben nach Konflikten gefragt, wird natürlich diese unterschiedlichen Erholungsnutzungen. Also ich glaube, dass sich das in den letzten Jahrzehnten immer mehr diversifiziert hat. Also man geht nicht mehr nur spazieren in den Wald, oder nur mal ein Stück wandern, sondern kommt mit dem Mountainbike, macht Geocaching und da entsteht natürlich zwischen diesen Nutzergruppen auch entsprechende Reibungen, die geregelt werden muss. Und die die Stadt sicherlich auch aktiv angehen sollte mit einem eigenen Konfliktmanagement. Und in Sachen Konfliktmanagement auch bewusst den Kontakt zur Bürgerschaft, also vertreten, warum nutzen wir das jetzt noch? Das Holz, den Wald? Warum lassen wir das nicht? Forstwirtschaft ist das gut oder böse? Also das hat ja oft die Tendenz, dass Leute gar keinen Bezug mehr dazu haben, dass ein Baum geschlagen oder verkauft wird, gerade im städtischen Umfeld. Es ist, ich glaube, das braucht es ganz, ganz viel Austausch.
9	**LT:** Mein zweites Thema sind die Auswirkungen der Covid-19-Pandemie auf die Nutzung des Stadtwaldes. Also, das hat man auch in Freiburg gemerkt, dass immer mehr Leute seit einem Jahr in den Wald gehen aus der Stadt. Und sehen Sie dort Konflikte?
10	**SP6:** Das sehe ich erstmal recht positiv. Ich weiß nicht, kennen Sie wahrscheinlich diese Studie aus einer deutschen Stadt, dem Wald dort, da war eine Zählung und man festgestellt, dass viel, viel mehr Menschen den Wald besucht haben und vor allen Dingen auch Gruppen, die sonst nicht so unbedingt waldbegeistert waren. Einfach weil es die einzige Möglichkeit war rauszugehen oder was anderes zu erleben und das sehe ich eigentlich, also das sehe ich eher positiv. Da wird die Bedeutung der Wälder nochmal anders. Es erfordert sicher eine Sensibilisierung, wenn viele Menschen in den Wald strömen, dass dann vielleicht Picknick und so weiter, dass dann auch eine entsprechende Rücksichtnahme erfolgt im Umgang mit dem Wald. Grundsätzlich denke ich aber auch, also ich denke, dass da auch eine Parallele da ist zwischen Covid-19 und Klimawandel. Es gibt wohl zwar einen Trend, sagt man, dass die Leute sich jetzt auch schon wieder Richtung Land orientieren, aber an sich war ja bisher immer die Annahme, die Leute ziehen in die Stadt. Und wenn da die Hitzewellen größer werden, dann geht es mehr, sucht man mehr Kühle im Umfeld und genau wie bei Covid-19, man sucht dann das auf, was man in unmittelbarer Umgebung hat und die Bedeutung wird entsprechend höher. Und ich könnte mir auch vorstellen für einer Stadt wie Karlsruhe ist dann die Frage Konfliktmanagement eine größere. Oder auch für, Sie meinten Konflikte, es ist ja auch immer die Frage, wie kommen die Menschen in den Wald? Und es geht dann vielleicht auch darum älteren Menschen barrierefrei einen Zugang zu gewährleisten, also das ist auch etwas, wo ich denke, dass da eine Stadt, ich weiß nicht wie es in dem Konzept von Karlsruhe mit drin ist, dann auch zu überlegen, welche Flächen biete ich denn in solchen Ausnahmesituationen wie bei Covid, wenn ich weiß, da kommen vielleicht mehr, wollen mehr Menschen ins Grüne, die muss ich dann wider verteilen. Oder in Zeiten des Klimawandels, die suchen Abkühlung. Wo schaffe ich das? Und nehme vielleicht andere Nutzungen da eher zurück.
11	**LT:** Und welche weiteren Lösungen auf politischer Ebene sehen Sie, um die Erholungsfunktion zu ermöglichen?
12	**SP6:** Also ohne, dass ich da im Detail weiß, wie die Abläufe jetzt sind bei der Erholungsplanung. Ich denke, wenn die Stadt selber Eigentümerin der Wälder drum herum ist, ist das sicher leicht möglich. Aber eine Verzahnung zwischen Erholungsplanung in der Stadt und im unmittelbaren Umland. Das fände ich wichtig. Also dass man, quasi wenn, dass man das miteinander kombiniert. Und ähnliche Kriterien dran legt und dass der Revierleiter da mitinvolviert ist und man auch schaut,

	wir haben Erholungsräume in der Stadt und das sind dann die Passagen zum Wald. Das fände ich wichtig. Und ansonsten, die anderen Geschichten sind natürlich, also meine Arbeitsstelle z.B. arbeitet gerade an der Frage Honorierung von Ökosystemleistungen. Das ist natürlich eine politische Entscheidung, die nicht Karlsruhe treffen kann, die natürlich weiter oben angesetzt ist, aber dass das was auch ein Forstbetreib eben investiert oder vielleicht ja auch verzichtet auf Einkommen, um eine Erholungsleistung zu schaffen, dass das anderweitig honoriert wird. Da gibt es ja auch Erhebungen, wie viel wären Menschen bereit zu zahlen, um in den Wald zu gehen. Das ist natürlich ein bisschen krass. Also das ist ja gerade in Deutschland schön, dass man in den Wald gehen kann, ohne dass das da abgesperrt ist. Aber das sind für mich so gerade politische Debatten, die auch in die Erholungsnutzung mitreinspielen, weil man letztendlich auch Eigentum mit nutzt, wo andere eine Verpflichtung haben das zu pflegen und wovon ich dann als Besucher profitiere. Also das wäre so eine politische Geschichte. Ansonsten ist es so, die, es gibt so, das würde ich aber sagen, das sind so Randthemen, die diskutiert werden. So die Frage, wie verändert sich die Natur? Was sind auch an Gefährdungen z.B. durch den Klimawandel zu erwarten im Wald? Was jetzt losgeht mit Allergenen durch bestimmte Pflanzen oder Baumarten, Birke, die vielleicht zunehmen, Das wäre vielleicht was, was eher so auf Stadtebene, wo man überlegen kann, was hat man da für Baumarten? Oder auch Verkehrssicherungspflicht wird schwieriger, wenn man mehr Dürreäste hat, abgestorbene Bäume. Das ist auch wieder was, was auch auf der kommunalen Ebene sicher. Aber da könnte ich mir vorstellen, dass da politisch anspruchsvoll ist, weil es eine Verzahnung braucht. Dass das Wissen da ist, wie verändert sich der Wald? Womit muss ich rechnen? Dann diese ganzen Debatten über *Vector-Borne Deseases*, mir fällt es gerade nicht ein, also Krankheitsüberträger, die eventuell profitieren könnten wiederum vom Klimawandel. Das ist was, da muss man zwar vor Ort reagieren, aber da kann man glaube ich, studienmäßig nicht im Blick haben, wie entwickelt sich da in Deutschland die Situation? Also dass man da durch Gesundheitsämter, durch die entsprechenden Debatten und die Verzahnung von Diskussionsprozessen, dass man da als Kommune auch eine Hilfestellung bekommt aus der politischen Richtung.
13	**LT:** Also eigentlich beantworten Sie gerade auch schon die nächste Frage von mir. Und zwar dass der Klimawandel und die Covid-19- Pandemie sehr globale, also sehr große Themen sind und Sie sehen jetzt aber schon, dass auch viele Handlungen auf unterster Ebene getätigt werden müssen?
14	**SP6:** Auf jeden Fall. Auf jeden Fall, also man sieht es auch an, wahrscheinlich ist das im Karlsruher Wald noch nicht Thema, aber hier in meinem Arbeitsort gibt es Gemeinden, die massiv betroffen sind vom Borkenkäferbefall, wo dann so ein großes Areal wegbricht und ich nehme an, dass der Stadtwald von Karlsruhe anders gebaut ist, dass da wahrscheinlich der Laubholzanteil groß ist, also dass der vielleicht mehr geschützt ist, aber in solchen Fällen, wenn der bestimmte, entsprechende Käferpopulationen oder Erkrankungen da sind, dann muss eine Kommune reagieren und gesteuert wird das aber von übergeordneten, globaleren Prozessen. Das stelle ich mir, wenn ich jetzt eine Kommune wäre auch schwierig vor, da immer den Blick zu haben, was könnte da in nächster Zeit, also auf der einen Seite sind sie natürlich nah dran, an ihrem Wald und sehen das einfacher und auch besser als Leute auf einer fachlich abgehobenen oder politischen Ebene und aber dieses Dranbleiben an Entwicklungen, oder Eichenprozessionsspinner. Ich kenne das aus einer anderen deutschen Stadt, da wurden wirklich mal einzelne Areale im Wald abgesperrt, und die Leute sind da eben, wegen des Eichenprozessionsspinners und der Gefährdung für allergische Reaktionen eben nicht in diesen Wald gegangen. In einer anderen mir bekannten Stadt wurde im Buchenwald, der ein beliebtes Gebiet war für die Erholung, ich weiß nicht, ob es letzten Sommer war, oder der davor, wurde abgesperrt, weil man gesagt hat, man kann die Verkehrssicherungspflicht nicht mehr gewährleisten, deshalb sehe ich das schon ein großes Thema auf kommunaler Ebene und ich finde auch, was mir dazu noch in den Sinn kommt ist, dass die Kommune ist ja letztlich, also wenn sie Waldeigentümerin ist gleichzeitig hat sie ja auch eine Chance zu steuern und da ist die einfach die richtige, also ich finde, da finde ich Kommunen auch prädestiniert, weil sie, ich meine, die haben den Kontakt zu ihrer Bürgerschaft, die können ein langfristiges Konzept machen und sagen, der Klimawandel kommt, wir müssen unsere Wälder ein Stück weit darauf vorbereiten, wir haben die

	und die Strategien, meinetwegen aktiv bestimmte Baumarten bevorzugen oder wir lassen da was passiv entwickeln. Und die können da ihre Bürgerschaft auch einbinden. Ich glaube, dass das entsprechend mehr passieren muss, weil die Leute recht anspruchsvoll geworden sind, was ihren Wald angeht. Also in meinem Arbeitsort habe ich direkt miterlebt, die wollen das nicht einfach vorgesetzt bekommen, das ist eine emotionale Bindung da und da war eine große Auseinandersetzung mit Fachbegriffen und Bewirtschaftungsthemen und das kann eine Kommune sehr gut leisten oder sollte es auch leisten. Es gibt erste Überlegungen die Forsteinrichtungen mit Dialogprozessen zu verknüpfen, also das ist ein Fachprozess, der sonst von wenigen besucht wurde, dass da auch Menschen dazu kommen können und ich glaube, dass das auch in der Stadt ein wichtiger Beitrag ist, auch ein bisschen zur Deeskalation, wenn unterschiedliche Ansprüche da sind.
15	**LT:** In Baden-Württemberg hat es jetzt nach einem Holzkartellverfahren eine Reformierung der Gewaltenteilung gegeben, also der Staatswald ist jetzt von der Anstalt der öffentlichen Rechts ForstBW verwaltet und der Körperschaftswald vom Forstamt und in Karlsruhe teilen sich jetzt die Besitzrechte des Stadtwaldes dadurch auf, weil ein Teil Körperschaftswald und aber auch ein Teil Staatswald ist in der Gemarkung Karlsruhe. Was ist da Ihre Meinung dazu?
16	**SP6:** In dem Thema bin ich nicht ganz so firm. Ich habe es nur mitbekommen, ich war mal eine Weile in einem anderen Arbeitsort unterwegs, dass das Thema bei den Forstleuten vor Ort sehr heiß diskutiert wurde und eher als eine Erschwerung ihrer Arbeit gesehen wurde, diese Aufteilung in, wer ist für was zuständig? Da bin ich zu wenig drin. Ich würde versuchen, wenn da eben unterschiedliche Eigentumsanteile sind, da trotzdem entsprechend verzahnt zu arbeiten. Aber es gab natürlich die Auflage, dass die Forstleute, dann eben nicht aktiv werden dürfen auf bestimmten Teilen. Also da kann ich leider wenig zu sagen, da bin ich nicht so richtig firm drin.
17	**LT:** Sie hatten gerade schon den Eichenprozessionsspinner angesprochen und zwar in diesem Konzept zur Klimaanpassung des Stadtwaldes von Karlsruhe wird auch zukünftig auf die Roteiche gesetzt, weil die ja sonst recht Trocken-, Hitzestress resistent ist. Wie würden Sie das bewerten?
18	**SP6:** Kann ich schwer bewerten ehrlich gesagt. Sie meinen auch mit dem Hintergrund, der Anfälligkeit für Eichenprozessionsspinner? Also aus Erwägung vom Klimawandel kann ich das gut nachvollziehen und ich glaube, meine Arbeitsstelle vertritt generell die These, in jedem Waldgebiet eben nicht nur auf eine Art setzten sollte, sondern dass man immer durchmischen sollte. Also so zu sagen, kein Wald ohne drei, vier Baumarten. Und damit hat man natürlich durch die Diversität dann schon einen gewissen Schutz erzeugt. Das ist auch, da gibt es leider noch wenig dazu, es gibt so ein Projekt *Doctor Forest* nennt sich das. Ich weiß nicht, ob Sie darüber gestolpert sind, das ist europaweit, aber an meiner Arbeitsstelle wird das koordiniert, die beschäftigen sich so mit der Frage, in wie weit Biodiversität schützen kann. Da wird sowohl eben bestimmte Krankheiten, Schädlinge werden betrachtet und da ist bisher, also sie haben noch wenige eigenen Daten, sie sind jetzt dabei, aber die These ist schon, dass sie davon ausgehen, dass eine ausreichende Biodiversität auch schützt. Also wenn man eben nicht Roteiche, man wird ja vermutlich keine Roteichenhaine anlegen, dass sich das dann untereinander auch ein Stück weit reguliert. Und diese These ist auch eben möglichst auch verschiedene Sachen auszuprobieren. Also wenn man merkt, das läuft nicht so gut, noch andere Flächen hat, auf denen man vielleicht auf was anderes gesetzt hat. Aber so richtig vorhersagen habe ich das Gefühl, da bin ich selber immer wieder erstaunt, also ich komme nicht aus der Waldforschung, aber habe mit vielen Leuten zu tun, die da Experten sind und die so oft sagen: “Das wissen wir nicht. Da haben wir keine Daten” oder “Das ist unsicher”. Also das was man überall liest, Unsicherheit, Komplexität, das finde ich da extrem, dass eben Menschen, die da schon seit Jahrzehnten arbeiten, vor dieser neuen Situation jetzt nicht die genauere Antworten haben. Ich antworte etwas weit auf Ihre Fragen, aber vielleicht nützt es ja was.
19	**LT:** Ja, wir sind auch schon beim Abschluss. Gibt es noch etwas, was Ihnen in dem Zusammenhang eingefallen ist, oder was Sie noch loswerden wollen?
20	**SP6:** Müsste ich kurz überlegen. Was ich einen Punkt finde, der in Teilen schon gemacht wird in Baden-Württemberg, das ist so eine Art auch soziales Monitoring zu machen, also wirklich auch als Stadt das kontinuierlich mitzudenken, nicht nur, wie entwickelt sich mein Wald im Hinblick auf

	Holzertrag auf bestimmte Baumarten, sondern mitzuschauen alle paar Jahre mal zu schauen, wie entwickelt sich die Erholungsnutzung, was fällt da auf, wo müssen wir umsteuern? Also auch da eine Art Monitoring aufzubauen. Das wäre noch. Muss man natürlich gucken wie das finanziell geht, was es da für Kapazitäten gibt, aber da gibt es ja auch Möglichkeiten über Forstliche Versuchsanstalt sich da einzuklinken. Ja das wäre noch ein Punkt.

SP7&8

1	**SP7 und SP8 (24:26.6 Minuten)**
2	**LT:** Was bedeutet für Sie naturnahes Waldmanagement?
3	**SP7:** Naturnahes Waldmanagement ist, wenn die Ökosystemleistungen des Waldes und zwar die vielen, die der Wald so zu bieten hat, berücksichtigt werden. Und dementsprechend auch die dann gefördert werden. Und jetzt ganz gezielt in der Bewirtschaftung ist es ja eigentlich so, dass man idealerweise einen Dauerwald sieht als idealen Waldtyp und versucht in der Bewirtschaftung dorthin zu gehen. Also weg von den früheren Monokulturen, wo mehr mit einer Baumart gearbeitet wurde, hin zu einem Waldtypen, der ganz viele verschiedene Ökosysteme innehat aber auch ganz viele verschiedene Funktionen erfüllen kann. Wo ganz verschiedene Arten von Bäumen drinstehen, die sowohl den Tieren, als auch dem Waldmikroklima sind ideale Voraussetzungen gibt.
4	**LT:** Die Stadt Karlsruhe hat in einem Konzept zur Klimaanpassung veröffentlicht, wo auch gesagt wird, dass sie das naturnahe Waldmanagement verfolgen. Wie würden Sie das bewerten?
5	**SP7:** Na erstmal gut.
6	**LT:** Welche Konflikte sehen Sie bei der Anwendung?
7	**SP7:** Bei der Anwendung sehe ich Konflikte zum einen -da muss ich kurz überlegen- zum einen darin, dass bestehende Waldbestände umgebaut werden müssen teilweise. Also in Karlsruhe ist ja auch viel, das ist im Oberrheingraben, sprich da ist viel Kiefernbestände mit Verjüngung darunter, die oft nicht standorttypisch ist. In Karlsruhe ist das viel Traubenkirsche oft mit drin. D.h. da muss, also auch Neophyten, die sich einfach mit reindrängen. Ich weiß, dass da viel die Kermesbeere auch ein großes Problem ist. Dagegen muss vorgegangen werden. Es muss geguckt werden, was zum Standort ideal passt. Also auf diesen trockenen Sanden wäre das, wenn man das jetzt und dem Klimaaspekt sieht, ist es vor allem Richtung Eiche oder Richtung Hainbuche. Da wird man mitarbeiten müssen und da versuchen müssen wie man diese Wälder ideal standortstypisch und standortstabil erhalten kann. Und das große Problem ist auch, das die Buche da ausfällt an diesen Standorten, weil es da sehr, sehr trocken ist, weil die sehr, sehr große Probleme hat mit dem Wasserhaushalt und da einfach gucken, wie kann man das, wie kann man mit den Gegebenheiten umgehen. Wie kann man die weiter fördern und halten auch.
8	**LT:** Und welche politischen Instrumente sehen Sie da als Lösung?
9	**SP7:** Politische Instrumente? Vom forstlichen her hat man erstmal die Forsteinrichtung, wo eigentlich festgeschrieben wird wie der Wald bewirtschaftet wird und die Forsteinrichtung wird zusammen mit dem Waldeigentümer, also das ist der Förster, der diese Bestände aufnimmt und schaut was drin steht, aber dann wird dieses Bewirtschaftungskonzept mit dem Waldeigentümer zusammen gemacht, d.h. da muss klar festgelegt werden, wie ich meinen Wald weiter bewirtschaften. Oder erstmal kurzfristige und mittelfristige Planung, aber dann fortgeschrieben die langfristige Planung über zehn Jahre oder darüber hinaus. Also welche Konzepte will ich reinkriegen. Was ist an bestimmten Orten zu fördern oder eben nicht zu fördern? Welche Schwerpunkte habe ich auch, wenn man jetzt in Karlsruhe guckt, da ist ja auch viel Erholungswaldnutzung. Ist der Erholungswald, setzte ich da einen starken Schwerpunkt drauf oder eher auf die forstwirtschaftliche, also die klassische Holznutzung? Ich habe Naturschutzaspekte, die berücksichtigt werden müssen. Es gibt auch Richtlinien, die übergreifend sind, z.B. FFH-

	Richtlinien oder Vogelschutzrichtlinien, die da reinspielen. Also diese Integration von diesen ganzen Konzepten in einem großen. Das sind so die Möglichkeiten, die man hat. Und natürlich eben auch die Relevanz von unseren Wäldern klar zu definieren. Das einfach auch gerade Stadtwälder eine ganz große Bedeutung haben für den Menschen und für die Umwelt und für alle die davon leben oder die davon leben möchten.
10	**LT:** Können Sie die Rolle des Stadtwaldes im Klimawandel noch genauer beschreiben?
11	**SP7:** Also die Rolle von Wäldern allgemein ist ja so, dass sie Luftfilter sind und Wasserfilter sind, also sehr viele Leistungen mitbringen, die wahnsinnig wichtig sind, auch für den Menschen. Und die Rolle von Stadtwäldern ist nochmal der besondere Fokus oder die Besonderheit von Stadtwäldern ist eben, dass der für einen ganz großen Teil der Bevölkerung, die nämlich in diesen Städten lebt, ganz schnell erreichbar ist und einen ganz großen Erholungswert für die Bevölkerung auch hat. Und in diesem Erholungswert, das kann jetzt sein über "ich verbringe da einfach Zeit" oder "ich übe da eine sportliche Tätigkeit aus" oder "ich bin mit meinen Kindern da", also der ist ganz, ganz vielfältig. So vielfältig die Bedürfnisse der Gesellschaft eben sind. Und der ist nicht zu verachten. Und das tolle am Stadtwald ist, dass er sehr, sehr nah ist und für alle erreichbar ist. Also egal aus welcher Schicht ich komme, egal wie alt oder jung ich bin, besteht die Möglichkeit diesen Wald aufzusuchen. Er ist umsonst, das ist ein ganz großer, ganz wichtiger Faktor auch. Dass es keine Beschränkung gibt, also nicht diverse Gruppen ausgeschlossen sind bei der Benutzung von diesen Wäldern. Dass Wald eben an sich eine ganz große Bedeutung für den Menschen hat, weil nachgewiesen wurde, oder weil bekannt ist, dass diese Erholungsleistung im grünen Umfeld, draußen sein, sowohl psychisch also auch physisch eine ganz hohe Bedeutung hat. Da gibt es ganz verschiedene Studien zu, ob es mit Kindern ist, die sich draußen im Wald bewegen und ihre Motorik mehr schulen oder Achtsamkeitstrainings, die gemacht werden können oder einfach nur das Sein im Wald. Deswegen ist es wahnsinnig wichtig Stadtwälder zu haben und das Zweite ist eben diese, ich nenne es jetzt mal, klimatische Komponente, dass Städte heizen sich ja wahnsinnig auf und man geht davon aus, dass im nächsten Jahr und Jahrzehnte Städte durch Fortschreiten des Klimawandels es einfach immer, immer heißer werden wird. Da haben Wälder eine wahnsinnig große Bedeutung als kühle Oasen in diesen Städten, weil eben dieses Aufheizen dadurch vermindert wird. Und das sind nicht nur die Wälder, sondern auch die Bäume, also auch der Alleebaum oder der Einzelbaum in der Stadt hat eine sehr, sehr große Bedeutung. Zusätzlich als Luftfilter, oder als Schadstofffilter, als Geräuschsenker. So diese Aspekte. Und eben für die Tiere, die auch im Stadtwald leben dürfen, können und wollen.
12	**LT:** Und wie finanziert sich diese Schutz- und Erholungsfunktion, wenn die Nutzfunktion in den Hintergrund rückt?
13	**SP7:** Ja nur indem wir dem einen Wert geben. Indem gesagt wird, dass das wichtig ist. Dass die Anerkennung von diesen Ökosystemleistungen, die jetzt nicht nur die reine, also als rein, ich hole Rohstoff aus dem Wald raus und habe dadurch was, was ich verkaufen kann, sondern eben Wertschätzung von Ökosystemleistungen, die sowohl naturschutzfachlich, also klimatisch sein können, als auch soziale Leistungen in Form von dieser Anerkennung der Gesundheitsleistung der Wälder und da muss oder müssten Werte definiert werden. Das wäre jetzt eine Aufgabe an die Politik, dass man diesen Ökosystemleistungen Wert zuschreibt und sagt, das ist uns so und so viel Euro oder Ausgaben einfach wert. Und in vielen Wäldern, in sehr vielen stadtnahen Wäldern ist es eben so, dass z.B. in meinem Arbeitsort kommen ja jetzt alle raus oder sind in meinem Arbeitsort viel unterwegs. Da ist es einfach so, dass die Stadt schon ein sehr, sehr großes Budget an die Erholungsleistung im Wald jedes Jahr stellt. Also sagt, uns ist in unserem Wald, wir machen da auch Forstwirtschaft, Forstwirtschaft ist super wichtig, aber die Erholungsleistung vom Wald hat auch ein sehr großen Wert und aus dem städtischen Haushalt gehen jedes Jahr -ich weiß nicht wie viel- aber mehrere Millionen Euro raus, um die Erholungsleistung des Waldes oder die Erholungsfunktion des Waldes zu fördern und die Menschen eben diesen Wald zugänglich zu machen. Und das kann sein in Form von Einrichtungen, die eben gestellt werden oder Waldwegen, die geöffnet werden, die gepflegt werden. In Form von gewissen Freizeitangeboten, die präsentiert werden. Also Mountainbiketrails oder sonstigen sportlichen Aktivitäten. Oder Mülleimer, die

	geleert werden, also es gibt ganz verschiedene Möglichkeiten, wo einfach der Waldbesitzer sagen kann, das ist mir was wert. Ich förder ja auch den Sportverein, so fördere ich auch meinen Wald als Ort des sozialen Ausgleichs, Gesundheitsförderung in meiner Stadt.
14	**LT:** Danke. Dann würde ich jetzt zum zweiten Themenblock schon kommen. Und zwar ist ja der Waldbesuch generell -da gibt es ja schon ganz viel Forschung dazu- wichtig für die physische und psychische Gesundheit und jetzt hat man gemerkt, auch in Karlsruhe, dass seit dem Ausbruch der Covid-19-Pandemie immer mehr Menschen aus der Stadt in den Stadtwald gehen. Welche Konflikte sehen Sie dort?
15	**SP8:** Wir haben uns das in meinem Arbeitsort angeguckt und haben auch verschiedene Arbeitsergebnisse durchgearbeitet aus anderen Städten. Und bei uns ist eigentlich in der Gesamtschau rausgekommen, dass die Konflikte jetzt nicht unbedingt zugenommen haben, quantitativ, also jetzt ist jetzt nicht unbedingt, dass es mehr ist. Es gibt andere Studien, die sich allerdings eher mit Förster*innen oder mit Naturschützer*innen beschäftigen und deren Perspektive auf die Zunahme, die nehmen sehr wohl Konflikte wahr. Z.B. dass Wege verletzt werden oder dass Leute einfach im Naturschutzgebiet rumlaufen, aber das konnten wir jetzt nicht zeigen. Dass es mehr geworden ist, aber Konfliktfelder gibt es eben neue. Also diese Problematik von *social distancing*, die jetzt durch die Pandemie aufgekommen ist, die gab es ja vorher in dem Ausmaß nicht und im Wald ist es so, dass viele Wege relativ schmal sind und dass man nicht unbedingt diesen Abstand so leicht einhalten kann. Das war für viele schon eine Herausforderung beim Waldbesuch, wenn sie sich erholen wollten, diese sozialen Abstandsregelgen einzuhalten. Das konnten wir schon sehen, dass das ein Konfliktfeld war, das wichtig war. Und was noch dazukommt ist, es gibt eine relativ große Gruppe von Leuten, die diese ruhige Walderholung bevorzugen, also gerne alleine im Wald sind, gerne ihre Ruhe haben, spazieren gehen, vielleicht auch meditieren im Wald und die sind natürlich eine relativ vulnerable Gruppe für diejenigen, die im Wald laut sind, die da Musik hören, die in großen Gruppen unterwegs sind. Für die ist dann natürlich ihre Erholung eingeschränkt. Das ist aber immer so, also das war vor Corona auch schon so und ist während Corona genauso geblieben. Was man sehen konnte ist, es gibt eine Möglichkeit dem zu entgehen indem man einfach weiter in den Wald läuft, also dass viele Leute einfach weitere Strecken zurückgelegt haben, um alleine zu sein, um ihre Ruhe zu haben.
16	**LT:** Und bei diesen neuen Konfliktfeldern, sehen Sie da Konsequenzen, die man jetzt ziehen kann für die Verwaltung der Stadtwälder?
17	**SP8:** Also konkret würde ich sagen, gibt es eigentlich zwei ganz konkrete Sachen, die man auf jeden Fall angehen könnte. Das eine ist diese *social distancing* Problem hat sich besonders gezeigt an Waldeingängen, weil da die Leute sich ballen, weil sie rauskommen und reingehen. Da könnte man, falls in der Zukunft sowas wieder passiert oder falls allgemein die Erholungsleistung, der Erholungsdruck auf den Wald zunimmt, könnte man natürlich gucken, dass man diese Nadelöhre so ein bisschen entzerrt, einfach durch räumliche Neugestaltung. Das wäre eine Möglichkeit und das gilt allgemein auch für Kreuzungen im Wald, wo sich viele Waldwege kreuzen, dass man da so ein bisschen guckt, dass die Übersichtlichkeit da so gegeben ist. Und allgemeiner ist es so, dieses Thema von Besucherlenkung im Wald, was ja relativ wichtig ist. Das ist ein zweischneidiges Schwert. Also auf der einen Seite ist das total wichtig, die Besucherströme zu lenken, damit die Leute auf den Wegen bleiben und so weiter. Andererseits haben wir auch gesehen, dass gerade die Möglichkeit, dass man sich freibewegt und dass man sich sozusagen nicht geführt fühlt für viele auch total wichtig ist. Also so dieses Gefühl zu haben, dass man sich frei bewegen darf. Deswegen dürfte man diese Besucherlenkung auch nicht zu strikt handhaben, damit dieses Gefühl nicht verloren geht.
18	**SP7:** Kann ich noch was ergänzen? Jetzt nicht bezogen, oder nicht nur bezogen auf Corona aber allgemein auf Besucher im Wald. Das ist so, ich weiß nicht, ob Sie den Aktivisten kennen, oder sagt Ihnen der Name von einem Projekt etwas beziehungsweise das Projekt von meiner Arbeitsstelle? Das ist so, dass aktuell und zwar gerade wirklich super aktuell in den Wäldern, also da im Landkreis des Bundeslandes, wo ich arbeite ein Projekt durchgeführt wird von meiner Arbeitstelle. Wo die Bevölkerung den Wald nutzt, selber ihre Waldwege einzeichnen kann und auch sagen kann, wo

	sind sie häufig, was gefällt ihr sehr gut und was gefällt ihr gar nicht im Wald. Also so *likes* und *dislikes* Punkte, was sie artikulieren kann. Auch Punkte, wo es Störungen aufgetreten sind, verschieden Arten, oder Sachen, die die Menschen gestört haben beim Waldbesuch. Und darauf oder auf diesen Grundlagen werden Karten erstellt und diese Karten geht in die Waldbewirtschaftung mit ein. Sprich die, also das ist einmal die Forsteinrichtung, es wird aber auch den Försterinnen und Förstern zur Verfügung gestellt, dass die einfach gucken können, wo sind Hotspot Bereiche, wo sind besonders viele Menschen? Wo muss ich Besucherlenkung machen? Wo sind vielleicht, wo treffen Fahrradfahrende und Spazierende aufeinander? Wo kann es zu Konflikten kommen? Wo habe ich auch vielleicht Konflikte im Naturschutz, also ich habe einen Habitatbaum und da sind immer besonders viele Menschen, jetzt kommt der Naturschutz und sagt, dieser Habitatbaum muss irgendwie geschützt werden. Wie gehe ich dann mit den Menschen um? Also es ist ein sehr gutes Tool eigentlich um dieses Waldmanagement und dem Wald an diese verschieden Interessensgruppen an den Wald zu vereinen. Also das ist, wie gesagt gerade aktuell auf der Homepage zu finden, das läuft gerade. Und das ist ein Projekt, das in dem Bundesland meines Arbeitsortes in den nächsten Jahren für die Staatswälder, oder für alle urbanen Staatswälder durchgeführt werden soll und im Nachgang wahrscheinlich auch für den Kommunalwald. Also das sind so Möglichkeiten, weil vorher war in der Forsteinrichtung nicht direkt der Besucher oder es gibt zwar ähnliche Projekte aber man hat nicht eine eins zu eins Aufnahme gehabt. Man wusste zwar wo viele Menschen sind, aber man wusste nicht genau, was die im Wald machen und wo die sich in dieser großen diffusen Fläche bewegen, wo auch Probleme vielleicht entstehen können. Und durch diese Verfeinerung durch dieses Projekts, durch diese Verfeinerung des Projektes ist es möglich und kann eins zu eins in diese Maßnahmenplanung mit übernommen werden. Und das birgt auch oder kann auch ganz viele Konflikte, die entstehen können einfach schon vorweggreifen, weil ich z.B. als Förster weiß, ich habe eine Holzentnahme und das da sind aber jetzt besonders viele Menschen, d.h. ich muss da besonders vorsichtig umgehen, muss da vielleicht besonders stark Öffentlichkeitsarbeit machen, muss besonders stark absperren, wohin gegen ich auch Bereiche plane, die dabei rauskommen, wo ich dann sehe, da sind überhaupt gar keine Personen, da kann ich dann anderes agieren, weil einfach der Konflikt eine anderer oder das Konfliktfeld ein anderes ist. Da kann ich Ihnen den Link mal schicken.
19	**LT:** Ja gerne. Also so soziales Monitoring sozusagen als politisches Instrument?
20	**SP7:** Genau also ein partizipativer Prozess ist das wo sowohl, also da kann jeder dran teilnehmen, der in den Waldgebieten unterwegs ist. Es werden gezielt Vereine, die viel im Wald sind darauf angesprochen und eben angeregt da mitzumachen.
21	**LT:** Interessant, danke. Ich habe noch ein paar allgemeine Fragen, auch an Sie beide dann einfach gerichtet. Und zwar erstmal in welchen Kategorien würden Sie die Qualität des Stadtwaldes messen?
22	**SP7:** Inwiefern? Ich muss erstmal definieren was für Kategorien ich.
23	**LT:** Nach welchen Kriterien könnte man auch sagen.
24	**SP7:** Letztendlich als Waldeigentümer auch was möchte. Möchte ich einen Erholungsschwerpunkt setzen oder eher einen Nutzungsschwerpunkt oder habe ich vielleicht auch Bereiche, wo Naturschutzschwerpunkte sind. Aber die Kriterien. SP8 verstehst du die Frage?
25	**SP8:** Nee, ich komme auch nicht so ganz dahinter. Es ist glaube ich sozusagen, es ist ein Aushandlungsprozess, also wie SP7 schon sagt, man muss diese Kriterien, die fallen ja nicht vom Himmel. Also es gibt da bestimmte Sachen, die man naturwissenschaftlich nachweisen kann, z.B. wie der Wald das Wasser reinigt und dann kann man das, glaube ich auch gut messen, ob der das hinkriegt oder nicht. Aber es gibt auch Sachen, die sich nicht messen lassen. Also wie gut es den Leuten gefällt oder wie erholt sie danach sind oder wie viel einem das wert ist, dass da ein bestimmter Vogel nistet. Das kann man jetzt schlecht in Zahlen erfassen. Das ist dann letztlich eine politische Entscheidung, die man treffen muss und da ist das Wichtige, dass sie getroffen wird.
26	**SP7:** Ja ich denke auch, es gibt so gewisse *Hardfacts*, das ist durch die Waldfunktionskartierung, wo einfach dann definiert ist, hier ist ein Wasserschutzgebiet oder hier ist auch ein

	Naturschutzbereich, der besonders wichtig oder schützenswert ist. Oder ein Bodenschtzwald, da hat der Wald schon eine besondere Bedeutung und da gibt es eben diese weichen Kriterien, das wären dann in dieser Kartierung Punkte wo Menschen dann Gefallen oder eben nicht gefallen, also wo die sagen, das ist ein Ort besondere kulturelle Bedeutung für mich hat oder besondere spirituelle Bedeutung für mich hat oder besondere Erholungsbedeutung für mich hat. Oder das ist ein Ort, der mir gar nicht gefällt, weil da jetzt ein wirtschaftlicher Eingriff war, mit dem ich überhaupt nicht zurechtkomme oder weil da Bäume stehen. Oder letzten Endes auch irgendwelche Pflanzen, die Allergien auslösen. Ich habe kein Problem mit Haselnusssträuchern, aber vielleicht mein Kollege und dann sagt der: "Nein, hier kann ich nicht hingehen." Und ich sage dann: "Nee, das ist hier doch alles ganz in Ordnung." Also es ist sehr schwer zu messen sowas. Aber über das letzte Projekt, die 2018, das in dem Bundesland, wo ich arbeite abgeschlossen wurde, hat man so versucht, dass man geguckt hat, wo sind Waldeingänge, also wo sind definitiv sehr viele Menschen, wo sind Parkplätze, wo ist ein besonderer Bereich und hat darüber versucht dieses Projekt auszubauen. Sowas wäre auch eine Möglichkeit. Oder wenn man die Distanz vom Zentrum, man sagt, im Durchschnitt braucht der -SP8 korrigiere mich, wenn ich jetzt falsch liege- aber ich glaube, der Erholungssuchende maximal eine viertel Stunde von seinem Wohnort in den Wald, also ist der Bereich vom Stadtende bis in den Wald viertel Stunde Distanz und sind so Erholungs-Hotspots so gesehen, weil da einfach sehr, sehr viele Personen sind, alles was danach ist, ist deutlich schwächer. Also so wären deren Bereiche.
27	**LT:** Jetzt sind ja der Klimawandel und die Covid-19-Pandemie sehr globale Angelegenheiten oder auch Probleme. Und finden Sie, dass die sich am besten auf lokaler, kommunaler Ebene angehen lassen oder sollten da nationale und internationale Lenkungen passieren?
28	**SP8:** Ich würde mal sagen, das ist ein ganz klassisches Sowohl-Als-Auch. Also es gibt ja jetzt beim Klimaschutz z.B. das Paris-Ziel, was das größte internationale Abkommen ist und das immer noch gefeiert wird und das klappt natürlich, aber nur, wenn die Sachen, die da beschlossen worden sind bis auf die kommunale Eben heruntergebrochen werden. Und das sehen wir aktuell schon, dass das in vielen Staaten schon auch auf der staatlichen Ebene scheitert. Und falls das der Fall ist, kann man z.B. auf kommunaler Ebene trotzdem was machen. Ich glaube, das ist ganz wichtig, dass sich da keiner aus der Verantwortung zieht. Das ist letztlich so ein Henne-Ei Problem. Wenn kein einzelner anfängt, dann ändert sich nichts. Aber nur weil ich jetzt nicht mehr in den Urlaub fliege, ändert sich nichts am Klimawandel. Da muss man sozusagen, da müssen sich alle an die Nase fassen und man darf trotzdem nicht vergessen, dass das ein strukturelle Problem ist.
29	**SP7:** Denke ich auch, das kann man auch ganz gut vergleichen mit Covid. Das bringt nichts, wenn die Regierung große Vorschriften macht und die Kommune, die einzelnen sich nicht dranhalten. Es muss wirklich auf beiden Ebenen stattfinden. Es muss von oben natürlich irgendwelche Richtungen geben, die gewisse Sachen klar definieren. Aber es muss auch eine Umsetzung stattfinden und die kann nur auf regionaler Ebene stattfinden. Und das ist dann auch in der Verantwortung von jedem einzelnen bzw. in den Städten oder in den Kommunen. Wo eben spezielle auch auf die örtliche Situation angepasste Konzepte umgesetzt werden müssen, also sowohl bei Corona, als auch beim Klimawandel. Ich brauche, kann nicht irgendwelche Baumarten fördern, wenn ich die Stelle nicht dazu habe und ich brauche keine Kinder zuhause lassen, wenn ich die Zahlen. Also man muss irgendwie gucken, was sind die Gegebenheiten des Örtlichen. Passt das, oder ist das so schräg dann? Aber natürlich muss es von oben die Regularien geben, weil sonst passiert gar nichts. Auf den Einzelnen zu vertrauen, dass der einzelne zukünftig nicht mehr in den Urlaub fliegt oder eine Maske aufhat. Wenn es da nichts von oben oder nicht irgendeine Weisung gibt, dann werden das einige tun, aber eben nicht alle.
30	**LT:** Es hat in Baden-Württemberg ein Holzkartellverfahren gegeben und daraus resultierte eine Reformierung der Gewaltenteilung und jetzt sind die Staatswälder verwaltet von der Anstalt des öffentlichen Rechts ForstBW und Körperschaftswälder vom jetzt z.B. in Karlsruhe immer noch von dem Forstamt. Was ist da Ihre Meinung dazu?
31	**SP7:** [23:39.5 – 23:49.4 nicht transkribiert, wegen Anonymität] Ja, es ist ein struktureller Prozess, hat einen Hintergrund, es gibt für alles Vor- und Nachteile. Es gibt Vorteile für das Einheitsforstamt,

	es gibt Vorteile für einen geteilten Betrieb. Möchte ich jetzt eigentlich nicht, kann ich jetzt nicht beantworten.
32	**SP8:** Ich habe mal vor ein paar Jahren so Projekte gemacht mit Forstwirten, also mit den Leuten, die im Wald arbeiten, da habe ich sie auch zu dieser Reform befragt, die da noch bevorstand. Da meinten sie auch, ja sehen wir mal schon, in zehn Jahren gibt es dann die nächste Reform und dann gucken wir mal weiter wie es dann so ist. Also ich glaube, das einzige, das uns weiterhin begleiten wird, ist dass es weiterhin Reformen geben wird.
33	**SP7:** Der Wandel.
34	**SP8:** Kann man natürlich auch als Qualitätsmerkmal der Reform betrachten, aber kann natürlich auch eine Anpassung an sich verändernde Gegebenheiten sein.
35	**LT:** Dann sind wir schon beim Abschluss. Gibt es noch irgendetwas, was Sie zu dem Thema sagen möchten? Generell, was Ihnen noch eingefallen ist während des Interviews?
36	**SP7:** Die Bedeutung. Die wahnsinnige Bedeutung von Stadtwäldern oder von Wäldern allgemein. Also sowohl für den Menschen, in Bezug auf das einzelne Individuum, die Erholung des Menschen draußen im Wald, als auch für den Klimawandel. Ist glaube ich, ja es steht mehr im Fokus als jemals zuvor und es sollte auch noch stärker in den Fokus rücken. Bei vielen, weil es einfach dermaßen wichtig ist.
37	**SP8:** Ich finde, das war ein sehr schönes Schlusswort.
